# Quality assurance of the SCOPE 1 trial in oesophageal radiotherapy

**DOI:** 10.1186/s13014-017-0916-7

**Published:** 2017-11-15

**Authors:** Lucy Wills, Rhydian Maggs, Geraint Lewis, Gareth Jones, Lisette Nixon, John Staffurth, Tom Crosby, on behalf of the SCOPE 1 trial management group and collaborators

**Affiliations:** 1 Department of Medical Physics, Velindre Cancer Centre, Cardiff, CF14 2TL UK; 2 Department of Clinical Oncology, Velindre Cancer Centre, Cardiff, CF14 2TL UK; 3 Wales Cancer Trials Unit, Centre for Trials Research, Cardiff University, Cardiff, CF14 1YS UK; 4 School of Medicine, Cardiff University, University Hospital Wales, Cardiff, CF14 4XN UK; 5 National Radiotherapy Trials QA (RTTQA) Group, Velindre Cancer Centre, Cardiff, CF14 2TL UK

**Keywords:** SCOPE 1 clinical trial, Radiotherapy, Quality assurance, Oesophageal cancer, Radiotherapy planning variation

## Abstract

**Background:**

SCOPE 1 was the first UK based multi-centre trial involving radiotherapy of the oesophagus. A comprehensive radiotherapy trials quality assurance programme was launched with two main aims:

1. To assist centres, where needed, to adapt their radiotherapy techniques in order to achieve protocol compliance and thereby enable their participation in the trial.
2. To support the trial’s clinical outcomes by ensuring the consistent planning and delivery of radiotherapy across all participating centres.

**Methods:**

A detailed information package was provided and centres were required to complete a benchmark case in which the delineated target volumes and organs at risk, dose distribution and completion of a plan assessment form were assessed prior to recruiting patients into the trial. Upon recruiting, the quality assurance (QA) programme continued to monitor the outlining and planning of radiotherapy treatments. Completion of a questionnaire was requested in order to gather information about each centre’s equipment and techniques relating to their trial participation and to assess the impact of the trial nationally on standard practice for radiotherapy of the oesophagus. During the trial, advice was available for individual planning issues, and was circulated amongst the SCOPE 1 community in response to common areas of concern using bulletins.

**Results:**

36 centres were supported through QA processes to enable their participation in SCOPE1. We discuss the issues which have arisen throughout this process and present details of the benchmark case solutions, centre questionnaires and on-trial protocol compliance. The range of submitted benchmark case GTV volumes was 29.8–67.8cm^3^; and PTV volumes 221.9–513.3 cm^3^. For the dose distributions associated with these volumes, the percentage volume of the lungs receiving 20Gy (V20Gy) ranged from 20.4 to 33.5%. Similarly, heart V40Gy ranged from 16.1 to 33.0%. Incidence of incorrect outlining of OAR volumes increased from 50% of centres at benchmark case, to 64% on trial. Sixty-five percent of centres, who returned the trial questionnaire, stated that their standard practice had changed as a result of their participation in the SCOPE1 trial.

**Conclusions:**

The SCOPE 1 QA programme outcomes lend support to the trial’s clinical conclusions. The range of patient planning outcomes for the benchmark case indicated, at the outset of the trial, the significant degree of variation present in UK oesophageal radiotherapy planning outcomes, despite the presence of a protocol. This supports the case for increasingly detailed definition of practice by means of consensus protocols, training and peer review. The incidence of minor inconsistencies of technique highlights the potential for improved QA systems and the need for sufficient resource for this to be addressed within future trials. As indicated in questionnaire responses, the QA exercise as a whole has contributed to greater consistency of oesophageal radiotherapy in the UK via the adoption into standard practice of elements of the protocol.

**Trial registration:**

The SCOPE1 trial is an International Standard Randomized Controlled Trial, ISRCTN47718479.

**Electronic supplementary material:**

The online version of this article (10.1186/s13014-017-0916-7) contains supplementary material, which is available to authorized users.

## Background

Definitive chemo-radiotherapy (dCRT) has an increasingly recognised role in the primary management of oesophageal cancer. SCOPE 1 was the largest multicentre trial of dCRT in localised oesophageal cancer in the UK, recruiting 258 patients from 36 centres. The trial investigated the addition of Cetuximab to standard radiotherapy (RT) plus cisplatin/fluoropyrimidine treatment [1], yet the phase II primary endpoint of 24 week treatment failure-free rate was not met and the trial closed on the basis of futility. Toxicity rates were higher in the dCRT + Cetuximab arm. Importantly however, survival rate in the standard dCRT arm is to our knowledge superior to any previous published multi-centre study [2]. More specifically, loco-regional recurrence was low in comparison to other studies and compromise in ability to give full RT dose associated with poor outcome, thereby suggesting the importance of RT [2]. It has been further proposed that the SCOPE QA programme was a crucial component to the successful outcomes seen in the cRT only arm [2].

**Fig. 1:**
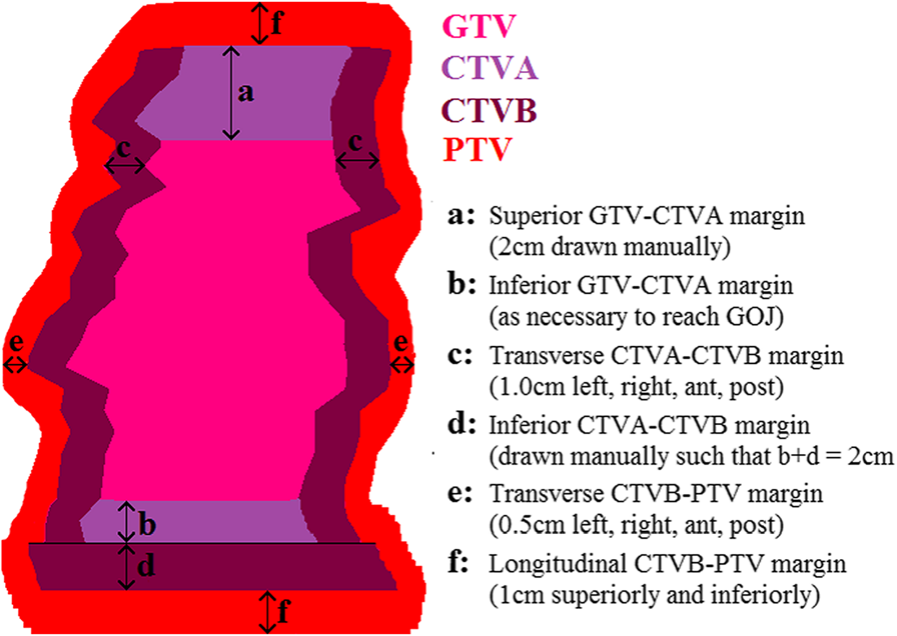
Target generation stages for SCOPE1

The radiotherapy schedule was identical in both arms of the trial (50Gy in 25 fractions over 5 weeks). The minimum requirement was for a 3D–conformal plan using contrast enhanced computed tomography (CT) with a minimum slice thickness of 3 mm to achieve the dose volume criteria in Table 2. The GTV was to be outlined with contrast enhanced diagnostic CT and endoscopic ultrasound, PET-CT was optional. Generation of the PTV from the GTV was to be done in accordance with the protocol (described in Table 1 and illustrated in Fig. 1). Verification of isocentre position and external patient contour was a requirement that could be managed according to the participating centre’s local protocols.

In order to support a trial’s clinical outcomes a Radiotherapy Trials Quality Assurance (RTQA) programme should provide evidence for adherence to the protocol thereby reducing the variation in RT planning and delivery to each recruited patient [3–5]. It requires good communication with participating centres, suitable infrastructure and software tools for analysis and sufficient man-power and expertise. Because of known variations in technique in oesophageal RT planning prior to SCOPE1 [6], the QA exercise aimed to ensure treatment delivery consistent with the SCOPE1 trial protocol [see Additional file 1] and evidence based best practice whilst assisting centres to adapt their RT techniques to enable their participation in the trial, and to provide ongoing support to RT centres throughout the UK.

The RTQA programme was implemented by the National RTTQA (Cardiff group) [7] which includes physicists, clinicians and radiographers at Velindre Cancer Centre in collaboration with a trial management team at the Wales Cancer Trials Unit (WCTU) based at Cardiff University. The methodology and results of the QA programme are described in this paper.

## Method

### Preparation of information for participating centres

Early collaboration between lead clinicians and QA physicists during the protocol development provided a basis for effective QA later in the trial. Areas of development which took place at this stage are summarised in Table 1. Detailed information concerning these topics were included as part of a pre-trial information package (CD-ROM) aimed at assisting centre’s acceptance into the trial. This contained the SCOPE1 protocol, a ‘RT planning and delivery’ document [see Additional file 2], examples of three compliant case studies (upper, mid and lower third) and software to enable their review (‘GUINESS’ (MSS Medical Software Solutions)) Two trial launch meetings organised by the trial co-ordinators (WCTU) were held in 2007. Presentations on the RT planning technique and the QA processes were made by members of the team.

**Table 1:** Summarised QA Team input to the development of trial documentation

Topic	Items included in the pre-trial CR-ROM
Patient preparation	Descriptions of ‘best practice’ for patient immobilisation and acquisition of the planning scan, administration of intravenous (IV) contrast and subsequent handling of contrasted images in the treatment planning system.
Structure delineation	Written and pictorial descriptions of the method for planning target volume (PTV) generation from the gross tumour volume (GTV) via the consecutive stages of clinical target volume (CTV), (‘CTVA’ and ‘CTVB’). Margin sizes were adopted from local practice [28], with multiple CTV stages formalised to correctly account for sub-clinical spread of disease along the line of the oesophagus, radially and below the level of the gastro-oesophageal junction (GOJ). In addition, a method for discretionary posterior modification of CTVB where close to the spinal cord was included.
Dose-volume criteria	Dose volume histogram (DVH) requirements (Table 2, column 1) for use with different types of dose calculation algorithms, namely ‘type a’ and ‘type b’ [11]. For ‘type b’, this required an investigative planning study [12], summarised later. A plan assessment form (PAF) [see Additional file 3] to maximise correct data return. Deviation levels for target coverage and OAR doses
Single phase planning	An illustrated planning guide including four problem examples. Use of a single phase plan, which had been shown to deliver lower heart doses than a widely used two phase approach using a ‘lung sparing’ anterior-posterior pair followed by a ‘cord sparing’ three field arrangement [29, 30] was mandated. Since this was not exclusively used in the UK at the time of the trial launch [6] the planning guide sought to assist with this transition, where required.
Treatment verification	An illustrated description of suitable pre-treatment and/or on-treatment verification processes aimed at ensuring accurate reproduction of the planned isocentre position and to manage significant changes to the patient’s external anatomy subsequent to planning.

### Pre-trial quality assurance

The ‘GUINESS’ visualisation platform, and subsequent versions (packaged as ‘VODCA’^1^ – Visualisation and Organisation of Data for Cancer Analysis) was used for analysis of submitted benchmark cases.

### Benchmark case requirements, analysis and feedback

For the benchmark case: an anonymised CT series was provided by the QA team which was common to all centres. The selected case included two elements which it was hoped would be challenging and thereby provide a suitable test of trial procedures and communication channels. Firstly the GTV length (8 cm) approached the upper allowable limit of the SCOPE patient entry criteria (10 cm): it was expected that plan dose volume objectives might not be achieved for all cases, prompting dialogue and checking procedures for data reporting. Secondly the GTV was close to the spinal cord, requiring application of a discretionary modification to the posterior part of the CTV as described in the ‘RT planning and delivery’ document.

### Outlining review

Outlining review was performed in part by an experienced treatment planning dosimetrist (LW) who assessed the OARs, the superior and inferior target extents with respect to the reference standard, the applied margins and the suitability of water density structures applied to correct for IV contrast (if used). The GTV was assessed qualitatively by the Chief Investigator (TC) by comparison to the reference standard. Images and/or description of any areas of outlining disagreement were fed back to each centre individually in the form of a written report.

### Planning review

Plan quality was assessed with respect to the SCOPE1 protocol requirements (Table 2, columns 1–3) and the International Commission on Radiological Units and Measurements (ICRU) reports [8, 9]. The dose volume objectives for the trial were based on a review of existing literature [10] and our own centre’s experience (at that time) of using a ‘pencil beam’ / ‘type a’ [11] dose calculation algorithm. During the early months of the trial, the need for greater clarity of plan criteria for centres using ‘type b’ algorithms was identified. A retrospective planning study was performed which concluded that the same OAR dose volume objectives should apply for both ‘type a’ and ‘type b’ algorithms [12]. The study also proposed a measure for individualised PTV coverage with accounted for the proportion of the PTV overlapping lung tissue (readily calculable in treatment planning systems) according to

1 $$ V95\%\ge 99-\left[0.4\times \% PTVoverlap\right] $$

Where ‘V95%’ is the volume of the PTV receiving 95% of the prescribed dose and ‘%*PTVoverlap*’ is the percentage of the volume of the PTV in lung tissue. Following this piece of work, the plan assessment form was updated and an E-mail bulletin was sent to participating and prospective centres which summarised the findings.

**Table 2:** Dose volume objectives, deviations, and benchmark case results (data for 36 cases)

Structure & Dose Volume Objective	Minor Deviation	Major Deviation	Range Achieved	Number of Deviations
Type a algorithm: PTV V95% > 99.0%	PTV deviations were not pre-classified but reviewed individually		98.6–100.0%	4 deviations (98.6, 98.8, 98.9, 98.9%)
Type a algorithm: PTV point minimum dose >93.0%			86.5–96.5%	2 deviations (86.5, 92.7%)
Type b algorithm: PTV V95% > 99.0% - [0.4 x % PTVoverlap]			92.4–99.3%	no deviations
ICRU maximum dose <107%	120% > max > 107%	max >120%	103–108%	2 minor deviations (108, 108%)
Heart V40Gy < 30.0%	50% > V40Gy > 30%	V40Gy > 50%	16.1–33.0%	1 minor deviation (33.0)
Liver V30Gy < 60.0%	70% > V30Gy > 60%	V30Gy > 70%	0.0–4.2%	no deviations
Combined lung V20Gy < 25.0%	35% > V20Gy > 25%	V20Gy > 35%	20.4–33.5%	18 minor deviations (6 of which >30%)
Spinal cord PRV D1cc < 40.0Gy	N/A	D1cc >40Gy	34.2–41.5Gy	2 major deviations (40.1, 41.5)

### QA of plan reporting

Benchmark case PAFs were checked to ensure that the requirements for reporting had been correctly interpreted and the recorded values agreed with an independent evaluation using VODCA. Having passed this aspect of protocol compliance, the centre’s PAFs were then used for real time checking of on-trial plan suitability by the QA team prior to treatment.

### QA of centre processes

A facility questionnaire was distributed in order to confirm compliance with aspects of the RT protocol which could not be determined from the benchmark case analysis, including immobilisation, pre-treatment checks and on-treatment verification. The questionnaire also sought to establish the extent to which centres were changing their practice in order to comply with trial requirements so that help could be offered if needed, and if participation in the trial had led to changes to local practice outside the trial.

### On-trial QA of clinical cases

Centres were required to send both a completed PAF and the anonymised full DICOM data, consisting of the planning CT images, structure set, plan and calculated dose for every patient. The upfront QA aims were firstly for every PAF to be checked as soon as received with respect to the dose volume objectives by trained trial management staff and for a QA physicist to be informed of any deviations from protocol. Secondly, for full retrospective individual case reviews to be performed by a QA physicist for the first clinical case from each centre, and a 10% sample from each centre thereafter. These were done using VODCA and / or CERR [13]. CERR (Computational Environment for Radiotherapy Research) is an open source application for viewing and analyzing RT data, written in MATLAB (MATLAB and Statistics Toolbox, The MathWorks, Inc., Natick, Massachusetts, United States). Reference was made to the centre’s benchmark case report in order to check for the successful resolution of any deviations noted and where issues were raised for on-trial cases, the RTQA team fed back to the centre and endeavored to assess a further case from the same centre to confirm resolution of the relevant aspects.

### Feedback and ongoing QA support

Written reports were provided detailing benchmark and subsequent patient case outcomes. Moreover dialogue was frequently engaged in between physics and/or clinical personnel enabling centres to access support throughout the trial.

## Selected results

Issues arising from both the benchmark case and the on-trial QA are summarised in Table 3 by topic and frequency.

**Table 3:** Summary of QA feedback

Topic	Percentage of benchmark cases requiring feedback	Percentage of on-trial cases requiring feedback
Outlining of GTV	72%	N/A
Target margins	39%	16%
Outlining of OARs	50%	64%
Cord PRV margin	33%	36%
Treatment Plan	22%	10%
Completion of the PAF	69%	42%

### Benchmark case results and feedback

The results here include data from 36 RT centres in the UK who submitted full benchmark cases. Nine of these centres did not recruit to the SCOPE l trial, yet their results are included in the benchmark case analysis. Where the results relate to target outlining, 54 investigators are included owing to submissions of outlines from additional clinicians from the 36 centres.

### Target generation

**Fig. 2:**
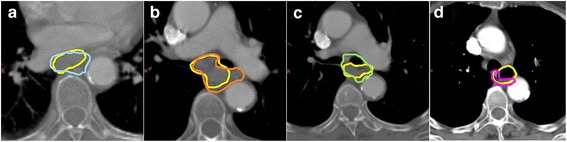
Examples of differences in interpretation of the GTV from the reference standard. In each image the reference standard is shown in yellow. **a** shows the unnecessary inclusion of the azygos vein; **b** is an example of the unnecessary inclusion of tissues surrounding the tumour; **c** shows the incorrect inclusion of the whole bronchus; and **d** shows a case where the anterior part of the oesophageal wall had not been included in the GTV

The average GTV was 47.1cm^3^ (SD 8.6cm^3^, range 29.8–67.8cm^3^). The reference standard GTV was 37.4cm^3^. The average PTV was 378.2cm^3^ (SD 47.7cm^3^, range 221.9–513.3 cm^3^). The reference standard PTV was 333.1cm^3^. Figure 2 shows the most commonly occurring minor issues highlighted to clinicians in transverse delineation of the GTV. GTV outlining has also been studied in greater detail as part of a sub-study by Gwynne et al. [14].

### OAR outlining

The radiotherapy procedures document described the superior level to which the heart should be outlined and included illustrations. Ten centres did not include three or four of the most superior expected slices. In five cases centres lung outlines included parts of the bronchi or trachea. Twelve centres had generated the cord PRV in only the transverse directions omitting to include a longitudinal margin as would be necessary to fully account for positional uncertainty.

### Dose volume results

Summarised dose volume results for the submitted benchmark cases are presented in Table 2 with the corresponding dose volume objectives and deviation levels. Major deviations were seen for the cord PRV dose in two cases. For each, following discussions, the centres were accepted to the trial on the understanding that cord PRV doses would be maintained to the 40Gy level where possible and that any deviations would be reported on the PAF for review prior to the patient starting treatment. For the greater of these deviations (D1cc = 41.5Gy) the QA team suggested an alternative solution, which when explored at the centre, also failed to meet the cord objective. However, the QA team was satisfied with the centre’s approach and level of engagement with the protocol so no further action was required. This case and others highlighted, as the analysis of benchmark cases progressed, the difficulty of assessing plans produced on a range of planning systems for a benchmark case which had not been pre-outlined. This is discussed later.

**Fig. 3:**
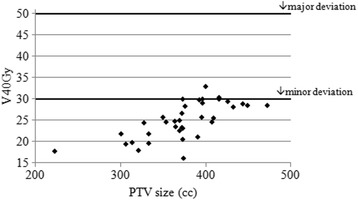
Heart V40Gy vs. PTV size for benchmark cases

**Fig. 4:**
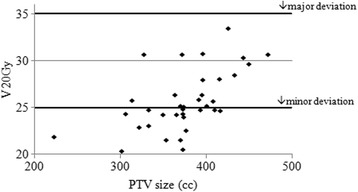
Lung V20Gy vs. PTV volume for benchmark cases

The range of heart and lung doses achieved are presented in Figs. 3 and 4 as a function of the PTV size, the minor and major deviation thresholds are also shown.

**Fig. 5:**
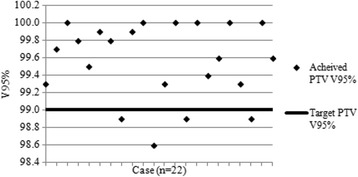
PTV coverage for benchmark case solutions planned with ‘type a’ algorithms (*n* = 22)

**Fig. 6:**
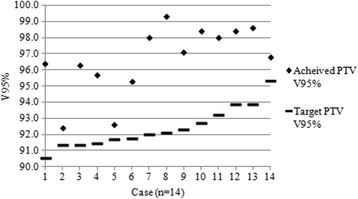
PTV coverage for benchmark solutions planned with ‘type b’ algorithms (*n* = 14)

Figure 5 shows the achieved PTV V95% with respect to the target level of 99% where planned using a type a algorithm. The target was not met in four plans: three cases (within 0.2% of the target) were attributed to a minor discrepancy between VODCA’s calculated result and the planning system used at the centre; the deviation in the final case (98.6%) was deemed acceptable since the consultant had given justification. Figure 6 shows the achieved PTV V95% for type b plans with respect to the target level, individually calculated using eq. (1): there were no deviations. As shown in Table 2 there were two deviations against a second PTV objective (point minimum dose >93.0%). These were associated with deliberate and justified compromises to a small section of the PTV in order to keep the cord PRV to within tolerance. However, following the previously mentioned planning study which resulted in the adoption of eq. (1) [12], it was evident that this objective was inappropriate for use with type b planning and the objective was removed entirely from the SCOPE 1 reporting requirements as a matter of simplification.

### Planning

**Fig. 7:**
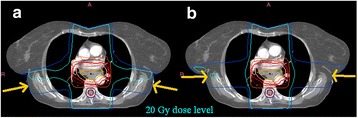
Example of a benchmark plan improvement via the QA process: Adjustment of the lateral beam orientations of the submitted plan (**a**) to better avoid the spinal cord PRV, allowed an increase in the contribution from the anterior and posterior beams, thereby enabling a greater level of lung sparing at the 20Gy dose level (**b**)

For eight plans the QA team offered one or more suggestions as to how the dose distributions could be improved upon and a revised plan was requested. An example case is shown in Fig. 7.

### Radiotherapy processes

Only 23 QA questionnaires were returned, some of these were from centres who did not recruit yet their results are included as the questionnaire explored changes to standard practice regardless of recruitment to SCOPE 1. The completed questionnaires represented 24 of 36 recruiting centres (including satellites), which contributed 161 patients (62%) to the trial. This level of response is discussed later. For all of the returned questionnaires centres demonstrated full compliance with procedures in relation to patient immobilisation, pre-treatment checks and on-treatment verification to at least the minimum standard as described in the protocol.

Centres underwent varying levels of adaptation to comply with the protocol. When asked if SCOPE had changed standard practice at the centre, 15 of the 23 centres who returned questionnaires (65%) stated that it had.

### On-trial QA

Two hundred fifty-eight patients were recruited to the trial from 36 centres (27 RT centres having completed a benchmark case and nine satellites of these). Retrospective individual case reviews (using full DICOM data) of the first patient from each centre were proposed. Owing to resourcing constraints, not all of the reviews were conducted around the time of the patient’s treatment. Indeed a proportion of the reviews were done retrospectively following the completion of the trial. DICOM data was available for 30 of 36 intended first patient reviews. For three centres the first patient data were not available, yet a subsequently recruited patient was able to be reviewed. The remaining three centres each recruited a single patient, yet no DICOM data was received and a full review could not be conducted.

### Target generation

A review was undertaken for all 33 cases with available DICOM data (as above: thirty first cases and three later cases where no DICOM data was available for the centres’ first case.).

It was possible to measure GTV length for 32 of the 33 cases. The Mean (SD) GTV length was 6.1 cm (2.0 cm), range = 2.3 to 9.9 cm). All were within the protocol limit of 10 cm. For 20 cases the GTV length recorded on the PAF and the length from the DICOM data were able to be compared: there was agreement for 12 cases, the largest discrepancy in the others was 1.3 cm. For all these discrepancies, the GTVs were quoted as being longer on the PAF than what was measured in VODCA. This is discussed later.

CTVB margins were determinable from DICOM for 32 plans. The superior and inferior margins were correct (2.0 cm or 2.1 cm) for 29 of the 32 having DICOM data. For the remaining three, the superior margin was within one slice (0.3 cm) of the protocol specification. The radial margin was correctly applied in all except one case.

PTV margins were correctly applied for 32 of the 33 cases reviewed. In one case superior and inferior margins of only 0.5 cm (instead of 1 cm) had been applied. Mean (SD) PTV volume was 314.7cm^3^ (112.4cm^3^) in the range 141.9 to 591.5cm^3^.

### OAR outlining

A review was undertaken for all of the 33 cases with available DICOM data.

Twenty-one centres plans included 1 or more minor deviations from the protocol in respect of outlining of OARs. The most common outlining deviations were inclusion of bronchi (11) and trachea (5) in the lung, under outlining the heart superiorly (12), and omitting to include a sup-inf element of the cord PRV margin (11). In one case no cord PRV had been generated.

### Dose volume results

A major deviation was associated with the non-generation of a cord PRV in one case. When created retrospectively, the PRV dose was found to exceed the protocol limits; this was the only on-trial instance of a major deviation from protocol. The error was reported back and was resolved for the centres’ second patient.

**Fig. 8:**
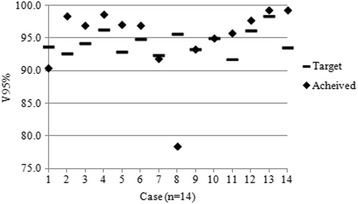
PTV coverage for reviewed on-trial cases planned with ‘type b’ algorithm (n = 14)

Nineteen plans used a type a algorithm and 14 used type b. Of the type a plans, two plans failed to achieve PTV V95% > 99%, yet were deemed to have been fully optimised. PTV coverage results for the type b plans are shown in Fig. 8. All centres had correctly calculated the individualised PTV V95% objective in accordance with the eq. (1). Three plans did not achieve the coverage target. In one case (case number 1 in Fig. 8) the low result was justified by the presence of significant air of the trachea and bronchi within the PTV. The remaining two (cases numbered 7 and 8 in Fig. 8) occurred prior to the outcome of the investigative study when centres had no specific guidance as to the acceptability of coverage of the 95% isodose, it was thought in these cases that the coverage at the superior or inferior extremes of the target region might have been improved by further optimisation. This is discussed later.

### On-trial support & commonly occurring on-trial planning issues

Two common themes emerged from discussions with the QA team. Firstly there was uncertainty as to the acceptability of dose distributions achieved using ‘type b’ algorithms. Initially advice was given on a case by case basis where, in the absence of specific requirements for PTV coverage and of detailed experience of every treatment planning system (a common issue for RTQA), deference was generally made to local knowledge and expertise. Following the publication by the QA team of the previously mentioned comparative study on this topic [12], and the circulation of a bulletin giving definitive advice, queries of this nature were eliminated. The second area of interest concerned solutions for planning when the targets were in very close proximity to the spinal cord. In response, a bulletin was circulated which summarised best practice and presented an optimisation solution. Additionally IMRT solutions, where proposed by participating centres, were permitted and quality assurance of these techniques was achieved with the assistance of colleagues in the wider national QA group.

## Discussion

### Overview

The pre-trial QA programme fulfilled a primary QA objective: it ensured that collaborating personnel in individual centres had read and understood the protocol and that centres had the necessary resources in place to deliver RT in accordance with the trial. The on-trial QA was to some extent limited by availability of resource at the QA centre. Our discussion herein focuses on areas which, with hindsight and more comprehensive resourcing may have been done differently to improve the completeness of QA data, or increase the efficiency of analysis in the context of this particular clinical trial.

### Benchmarking with pre-outlined cases

Ability to advise centres on the optimality of their benchmark case solution can be limited owing to differences in treatment planning systems and compounded by variations in target delineation. This problem can be lessened by providing both a non-outlined benchmark case to test outlining accuracy and a pre-outlined benchmark case to test planning to standardised geometry. Dosimetric results for the latter can be more easily compared to an expected outcome. This methodology is noted by Ibbott et al. [3] and now used by the RTTQA group where warranted, according to a tiered system of RT complexity as described by Bekelman et al. [15]. Whilst differences due to planning system and algorithm would remain, it may not be unreasonable, given the extent of collaboration within national RTTQA at time of writing, to explore these variations prospectively for an RTQA programme. The use of a single benchmark case for both outlining and planning in SCOPE1 does however reveal the effect of differences in GTV outlining on the eventual dose distribution as presented above.

### Recommendations for future trials QA

The pre-trial QA processes did not eliminate the recurrence of inconsistencies from protocol seen in benchmark cases, particularly in the outlining of OARs (where inconsistencies increased). This could be due to insufficient training and dissemination within centres of the outcomes and recommendations of the QA exercise and may point to the need for more rigorous processes at recruiting centres (an individual patient checklist, for example). More recent development in on-line real-time and timely retrospective outlining and plan review will also address this [14, 16]. The implications of these minor inconsistencies are unlikely to have any impact on the SCOPE 1 clinical conclusions in our opinion. Indeed, an upfront, risk assessed approach, to identify aspects of the treatment delivery chain which are likely to affect the ability to answer the trial question, such as is recommended by Ibbott et al. [3] can be applied in order to rationalise the use of QA resource.

The variation in GTV outlining of the benchmark case, quantified by Gwynne et al. [14] demonstrated the presence of a range of both methodology and precision applied for this treatment site. Within the SCOPE 1 QA process, each investigator was provided with comments from the CI: effectively they participated in an external audit of their GTV definition. Feedback such as this had not been commonly available elsewhere within clinical practice and was a first in oesophageal RT in the UK. However, improvements in the process were identified as follows: the SCOPE 1 reference standard was based on the interpretation two individuals (the CI and a radiologist). The need for a more robust reference standard became a point of discussion throughout the QA cycle as alternative legitimate clinical interpretations were observed in benchmark solutions. Variation in target delineation is a widely known and universal issue in RT [17–20], the need for ‘consensus guidelines’ with dissemination and training, is re-emphasised here. This approach is being widely incorporated into trials QA systems and RT more generally [21–24].

In our comparison of outlined GTV length against that quoted on the PAF, discrepancies were seen, always in the same direction (PAF GTV longer than VODCA GTV). This could be attributed to the method of measurement used by centres: if the operator were to use a software measure tool placed along a sectional reconstruction of the CT series the value measured would always be equal to or longer than the length calculated based on the longitudinal coordinates of the extents of the GTV. This is because the organ may lie at a diagonal to the longitudinal. Furthermore, up to one slice thickness may be added if the operator interprets the length to be ‘the number of slices outlined multiplied by the slice thickness.’ In this case the operator is assuming that each slice is representative of a volume of patient rather than a 2-dimensional level. In the absence of knowledge of exactly how each operator interpreted the value of GTV length requested on the PAF, this uncertainty remains. Future studies should therefore define how lengths, if needed, are to be calculated, or request the raw data (slice positions) in order to enable a calculation of the parameter.

The return rate of the QA questionnaire was poor (53% of recruiting centres), despite encouragement from WCTU at various stages throughout the trial. It was intended to serve a dual purpose: firstly to check that elements of practice at each centre met trial criteria, and secondly to assess the impact of the trial on changes to standard practice. For the latter reason the questionnaire was not made a pre-requisite to centre recruitment. Rather, centres were given time to reflect on any changes that had been introduced into standard practice. The result of not mandating a response to the questionnaire prior to recruitment may have contributed to a loss of momentum of its return. With this hindsight, it would have been better not to combine the two aspects, instead to require a response before centres were given QA approval. Notwithstanding a disappointing return, the responses, as summarised earlier, indicate that quality and consistency of practice across the UK increased as a result of SCOPE1. In addition, outside of oesophageal RT, the description of heart delineation for SCOPE1 has been used, with permission, in subsequent trial protocols for the Fast-Forward [25] and I-START [26] trials.

### Reflections on the PTV coverage target for type b algorithms and future use

The investigative planning study [12] provided a standard for PTV coverage. The benefit of this piece of work is demonstrated here as all of the type b deviations for PTV coverage according to the criteria suggested by the study, occurred prior to its outcome. This suggests that the guidance, once issued, provided greater clarity as to the acceptability of distributions, enabling planners to persist with the optimization process until at least the suggested minimum had been achieved. For future trials, if conducted with access to improved optimisation techniques, a higher standard of PTV coverage could be specified simply by adjustment of the multiplier (currently 0.4) within the formula.

## Conclusions

The benchmark case successfully identified and advised participating centres of the areas of their RT technique which were inconsistent with the SCOPE 1 protocol in advance of recruitment. On-trial QA revealed no more than a single major deviation (relating to cord PRV dose) for patients treated within SCOPE 1, thereby supporting the clinical conclusions. However, for recruited patients there did appear to be repetitions of minor inconsistencies of technique, revealed retrospectively, highlighting the need for sufficient QA systems and resource to ensure a continuous feedback process throughout the progress of an RT trial. The range of patient planning outcomes for the benchmark case is illustrative of the potential variation in oesophageal treatments at the outset of the trial, which are largely due to differences in interpretation of the GTV, despite the presence of a protocol. This supports the case for increasingly detailed definition of practice by means of consensus protocols, training and peer review. Further exercises in oesophageal RT QA may in the future be able to draw comparisons to this piece of work. The QA exercise as a whole has facilitated greater consistency of oesophageal RT in the UK via the widespread adoption into standard practice of many elements of the protocol, and has contributed, in part, to wider collaboration in UK Oesophageal radiotherapy [27].

## Additional files

Additional file 1: SCOPE1 Trial Radiotherapy Protocol. (PDF 988 kb)

Additional file 2: SCOPE1 Trial Radiotherapy Procedures Document. (PDF 2300 kb)

Additional file 3: SCOPE1 Trial Plan Assessment Form. (PDF 165 kb)

## Abbreviations

CT: Computed tomography
CTV: Clinical target volume
dCRT: Definitive chemo-radiation
DVH: Dose volume histogram
GTV: Gross tumour volume
IMRT: Intensity modulated radiotherapy
IV: Intravenous
OAR: Organ at risk
PAF: Plan assessment form
PRV: Planning risk volume
PTV: Planning tumour volume
QA: Quality assurance
RT: Radiotherapy
RTQA: Radiotherapy trials quality assurance
VMAT: Volumetric modulated arc therapy
WCTU: Welsh cancer trials unit

## Footnotes

1
http://www.vodca.ch/
