# Supplementary material for: Quality assurance of the SCOPE 1 trial in oesophageal radiotherapy
Source: Radiat Oncol. 2017 Nov 15;12:179. doi: 10.1186/s13014-017-0916-7 (PMC5688711; doi:10.1186/s13014-017-0916-7)
Supplement: Supplementary file 1 — SCOPE1 Trial Radiotherapy Protocol. (PDF 988 kb) [file 13014_2017_916_MOESM1_ESM.pdf]

SCOPE 1

SCOPE 1: Study of Chemoradiotherapy in Oesophageal Cancer Plus or Minus Erbitux

A randomised phase II/III multicentre clinical trial of definitive chemoradiation, with or without Cetuximab, in carcinoma of the oesophagus

CLINICAL PROTOCOL, Version 4.0, 13th November 2008

EUDRACT No. 2006-002241-37
ISRCTN: 47718479

SPONSOR: Velindre NHS Trust

Authorised by:

Name:	Dr Tom Crosby	Role:	Chief Investigator
Signature:	[signature]	Date:	13/11/08.
Name:	Gareth Griffiths	Role:	Scientific Director WCTU
Signature:	[signature]	Date:	14/11/08

WCTU WALES CANCER TRIALS UNIT UNED YMCHWIL CANSER CYMRU
YMDDIRIEDOLAETH GIG FELINDRE
VELINDRE NHS TRUST
CANCER RESEARCH UK
NCRI National Cancer Research Institute

Developed on behalf of the NCRI Upper GI Clinical Studies Group.

General Information

This document describes the SCOPE 1 trial, and provides information about procedures for entering patients into it. The protocol should not be used as an aide-memoire or guide for the treatment of other patients. Every care was taken in its drafting; however, corrections or amendments may be necessary. These will be circulated to the known investigators in the trial, but centres entering patients for the first time are advised to contact the Wales Cancer Trials Unit in Cardiff to confirm that they have the most up-to-date version of the protocol in their possession.

Sponsor

Velindre NHS Trust, Corporate Headquarters, Unit 2 Charnwood Court, Parc Nantgarw, Nantgarw, Cardiff, CF15 7QW

Funding

Clinical Trials Advisory and Awards Committee (on behalf of Cancer Research UK, Medical Research Council, and other charities). Cetuximab is provided free of charge by Merck KGaA.

Compliance

This trial will adhere to the conditions and principles which apply to all clinical trials as outlined in the Medicines for Human Use (Clinical Trials) Regulations 2004, as amended, EU Directive 2001/20/EC, EU Directive 2005/28/EC and the ICH Harmonised Tripartite Guideline for Good Clinical Practice (CPMP/ICH/135/95). It will be conducted in compliance with the protocol, the Medicines for Human Use (Clinical Trials) Regulations 2004, SI 2004 1031, as amended, the Research Governance Framework for Health and Social Care (Welsh Assembly Government November 2001 and Department of Health 2 July 2005), the Data Protection Act 1998, and other regulatory requirements as appropriate.

RANDOMISATION

Telephone the Wales Cancer Trials Unit on

029 2064 5500

Between 0900 and 1700 hours (Monday to Friday)

SAE FAX NUMBER:

029 2064 4488

Chief Investigator

Dr Tom Crosby
Consultant Oncologist
Velindre Hospital
Cardiff CF14 2TL

Co-Investigators

Dr Ian Geh
Consultant Clinical Oncologist
Queen Elizabeth Hospital
Birmingham B15 2TH

Dr Simon Gollins
Consultant Clinical Oncologist
North Wales Cancer Treatment Centre
Glan Clwyd Hospital, Bodelwyddan
Rhyl LL18 5UJ

Advisors

Clinical Advisors

Dr Stephen Falk
Consultant Clinical Oncologist
Bristol Haematology and Oncology Centre
Horfield Road
Bristol BS2 8ED

Prof David Cunningham
Head of the Gastrointestinal Unit
The Royal Marsden NHS Foundation Trust
Downs Road, Sutton
Surrey SM2 5PT

Dr S. Ashley Roberts
Department of Radiology
University Hospital of Wales
Heath Park, Cardiff
CF14 4XW

Prof. Geraint T Williams
Consultant Histopathologist
Pathology Directorate
University Hospital of Wales
Heath Park, Cardiff
CF14 4XW

Nursing

Wendy Wade
Oncology Research Practitioner
Clinical Trials Unit
Velindre Hospital
Cardiff CF14 2TL

Patient Representative

David Kirby
22 Vulcan House, Vulcan Road,
Solihull,
West Midlands
B91 2JY

Radiotherapy Quality Assurance

Dr John Staffurth
Clinical Senior Lecturer/Honorary Consultant
Department of Clinical Oncology
Velindre Hospital
Cardiff CF14 2TL

Lucy Wills
Senior Dosimetrist
Radiotherapy Planning Department
Velindre Hospital
Cardiff. CF14 2TL

Radiotherapy

Stephen Slade
Superintendent Radiographer
Radiotherapy Department
Velindre Hospital
Cardiff. CF14 2TL

Pharmacy

Bethan Tranter
Pharmacy Department
Velindre Hospital
Cardiff CF14 2TL

Translational

Dr John Bridgewater
Department of Oncology Royal Free & University College Medical School
5th Floor Courtauld Building
91 Riding House Street
London W1W 7BS

Rebecca Fitzgerald
MRC Cancer Cell Unit
Hutchinson/MRC Research Centre
Hills Road
Cambridge CB2 2QQ

Health Economics

Prof Ceri Phillips
Centre for Health Economics and Policy Studies
School of Health Science
Swansea University
Swansea SA2 8PP

Quality of Life

Prof. Jane Blazeby
Bristol Royal Infirmary
Marlborough Street
Bristol BS2 8HW

The SCOPE 1 trial is being run by the Wales Cancer Trials Unit, a NCRI accredited clinical trials unit.

Coordinating Centre

Wales Cancer Trials Unit	Tel:	+44 (0) 29 2068 7500
6th Floor, Neuadd Meirionnydd	Fax:	+44 (0) 29 2068 7501
Heath Park	Email:	wctu@wctu.wales.nhs.uk
CARDIFF	Website:	www.wctu.org.uk
CF14 4YS		

SCOPE 1 Email Address: scope1@wctu.wales.nhs.uk

Wales Cancer Trials Unit Staff

**Trial Manager:**		
**Lisette Nixon**	**Tel:**	**+44 (0) 29 2068 7459**
	**Email:**	**Lisette.Nixon@wctu.wales.nhs.uk**
Scientific Director		
Gareth Griffiths	Tel:	+44 (0) 29 2068 7456
	Email:	Gareth.Griffiths@wctu.wales.nhs.uk
Clinical Director		
Tim Maughan	Tel:	+44 (0) 29 2068 7500
	Email:	Tim.Maughan@wctu.wales.nhs.uk
Senior Statistician:		
Angela Casbard	Tel:	+44 (0) 29 2068 7470
	Email:	Angela.Casbard@wctu.wales.nhs.uk

For all queries please contact the SCOPE 1 Trial Manager.

TABLE OF CONTENTS

Table of contents **5**
Abbreviations and Glossary **8**
Trial Schema **10**
Trial Synopsis **11**
Summary **13**
1.1 Lay Summary 13
1.2 Abstract and summary of trial design 13
2. Introduction and Rationale **14**
2.1 Background 14
2.2 Chemoradiotherapy in oesophageal cancer 14
2.3 Rational for standard chemotherapy agents 14
2.4 Cetuximab and anti-EGFR therapies 15
2.5 Rationale and objective 17
3. Participating Centre Selection **18**
4. Trial Entry **19**
4.1 Patient Entry 19
4.1.1 Inclusion criteria 19
4.1.2 Exclusion criteria 20
4.2 Pre-treatment procedures 21
4.3 Randomisation 22
5. Assessements **23**
5.1 Baseline (pre-treatment) 23
5.2 Assessments during and at end of treatment 23
5.3 Post treatment (week 24) 23
5.4 Follow up 24
5.5 Schedule of Assessments 25
5.6 Schedule of Treatment 26
6. Treatment Regimen and Schedules **27**
6.1 Study treatments 27
6.1.1 Treatment summary 27
6.2 Treatment schedules 28
6.2.1 Arm A: Cisplatin + capecitabine (Control Arm) 28
6.2.2 Arm B: Cetuximab + cisplatin + capecitabine (Research Arm) 30
6.3 Non trial treatment in the research arm 32
6.3.1 Medications permitted 32
6.3.2 Medications permitted with caution 32
6.3.3 Non permitted medications 32
7. Clinical considerations **34**
7.1 Pregnancy 34
7.2 Nutritional status 34
7.3 DPD deficiency 34
8. Drug Supply, dosing, side effects and modifications **35**
8.1 Drug Supply 35
8.1.1 Cisplatin and Capecitabine (5FU) 35
8.1.2 Cetuximab 35
8.2 Dosing and precautions 36
8.2.1 Cisplatin and capecitabine (5FU) 36
8.2.2 Cetuximab 38

8.3 Possible side Effects 41
8.3.1 Cisplatin and Capecitabine (or 5FU) 41
8.3.2 Cetuximab 42
8.4 Dose modifications 43
8.4.1 Cisplatin and capecitabine (or 5FU) 43
8.4.2 Cetuximab dose modifications / criteria for stopping 46
Cetuximab skin toxicity 47
9. Radiotherapy 48
9.1 Definition of Treatment Volumes 48
9.1.1 Gross Tumour Volume 48
9.1.2 Clinical Target Volume 49
9.1.3 Planning Target Volume 49
9.1.4 Lower 1/3rd tumours 49
9.1.5 PTV coverage 49
9.2 Organs at Risk 50
9.3 Normal Tissue Tolerance 50
9.4 Treatment delivery 50
9.5 Treatment Gaps 51
10. Trial Conduct and Monitoring 52
10.1 Measures of concordance / adherence 52
10.2 Data Handling 52
10.3 Loss to follow-up 52
10.4 Central monitoring 52
10.5 Direct access to data 52
10.6 Quality assurance and quality control of data 53
10.7 End of trial 53
10.8 Patient withdrawal 53
10.8.1 Withdrawal from trial treatment 53
10.8.2 Withdrawal from trial completely 54
11. Safety Reporting 55
11.1 Centre responsibilities 57
11.2 Reporting pregnancies whilst participating in SCOPE 1 58
11.3 WCTU responsibilities 58
12. Statistical Considerations 60
12.1 Method of randomisation 60
12.2 Outcome measures 60
12.3 Sample size calculation 61
12.4 Interim monitoring and analyses 61
12.5 Statistical analyses 62
12.6 Subgroup analyses 62
13. Informed Consent, Ethical & Regulatory Considerations 63
13.1 Ethical and other issues 63
13.2 Regulatory status 63
13.3 Sponsorship and indemnity 64
13.4 Data protection 65
13.5 Publication policy 65
13.6 Finance 65
14. Quality of Life 66
15. Health Economics 68
16. T-SCOPE (Translational Laboratory Research) 69

17. Quality Assurance of Radiotherapy 70
17.1 Educational component 70
17.2 Test Case 70
17.3 Questionnaires....70
17.4 Equipment validation 70
17.5 Patient cases 70
18. Trial Committees and Trial Management Arrangements....71
19. REFERENCES 72
Appendix I 77
WHO Performance status 77
Appendix II 78
Renal Function....78
Appendix III 79
WHO grades of hearing impairment 79
Appendix IV....80
Investigations 80
Appendix V....82
Quality of life questionnaire EORTC QLQ-C30 82
Appendix VI....84
Quality of life questionnaire Oesophageal Module 84
Appendix VII....85
Dermatology Life quality Index 85
Appendix VIII....87
Your Current State of Health 87
Appendix IX....89
Collection, Preparation and Storage of Samples....89
Appendix X....91
Dysphagia Scale 91
Appendix XI....92
CTCAE Toxicity criteria....92
Appendix XII....96
RTOG/EORTC Late Radiation Morbidity Scoring Schema....96
Appendix XIII....98
RECIST criteria 98

ABBREVIATIONS AND GLOSSARY

**18-FDG PET**	^{18}F-labeled 2-deoxy-2-fluoro-D-glucose Positron emission tomography
**5FU**	5-Fluorouracil
**AC**	Adenocarcinoma
**AE**	Adverse event
**ALP**	Alkaline Phosphatase
**ALT**	Alanine Aminotransferase
**ANC**	Absolute Neutrophil Count
**AR**	Adverse reaction
**ASCO**	American Society of Clinical Oncology
**AST**	Aspartate aminotransferase
**AUC**	Area under the concentration-time curve
**BD**	Twice daily
**BSA**	Body surface area
**C**	Cisplatin
**CF**	Consent form
**CI**	Chief Investigator
**CRF**	Case Report Form
**CRT**	Chemoradiotherapy
**CSG**	Clinical Studies Group
**CT**	Computerised tomography
**CTV**	Clinical target volume
**CTAAC**	Clinical Trials Advisory and Awards Committee
**CTA**	Clinical Trials Authorisation
**CTCAE**	Common Terminology Criteria for Adverse Events
**CTU**	Clinical Trials Unit
**DVH**	Dose volume histogram
**DLQI**	Dermatology Life Quality Index
**EDW**	Electronic dynamic wedge
**EGF**	Epidermal growth factor
**EGFR**	Epidermal growth factor receptor
**ERC**	Endpoint review committee
**ESMO**	European Society for Medical Oncology
**EUS**	Endoscopic ultrasound
**EUDRACT**	European Union Drug Regulatory Agency Clinical Trial
**FBC**	Full Blood Count
**FNA**	Fine needle aspiration
**FSD**	Focus to skin distance
**GI**	Gastrointestinal
**GOJ**	Gastro-oesophageal junction
**GTV**	Gross tumour volume
**HE**	Health economics
**IB**	Investigator's Brochure
**ICRU**	International Commission on Radiation Units and Measurements
**IDMC**	Independent data monitoring committee
**ISF**	Investigator site file

**ISRCTN**	International standard randomised controlled trial number
**MDT**	Multidisciplinary team
**MHRA**	Medicines and Healthcare products Regulatory Agency
**MLC**	Multi leaf collimator
**MRC**	Medical Research Council
**NCI**	National Cancer Institute
**NCRI**	National Cancer Research Institute
**NHS**	National Health Service
**OARs**	Organs at risk
**OGD**	Oesophagogastroduodenoscopy
**OS**	Overall survival
**PDQ**	Physician data query
**PEG**	Percutaneous endoscopic gastrostomy
**PET**	Positron emission tomography
**PI**	Principal Investigator
**PIS**	Patient information sheet
**PO**	Perorally
**PRV**	Planning risk volume
**PTV**	Planning target volume
**PVI**	Protracted venous infusion
**QoL**	Quality of life
**R&D**	Research and Development
**RT**	Radiotherapy
**RTOG**	Radiation Therapy Oncology Group
**SAE**	Serious adverse event
**SAR**	Serious adverse reaction
**SCC**	Squamous cell carcinoma
**SPC**	Summary of product characteristics
**SOP**	Standard operating procedure
**SSA**	Site specific assessment
**SUSAR**	Suspected unexpected serious adverse reaction
**TK**	Tyrosine kinase
**TMF**	Trial master file
**TMG**	Trial management group
**TSC**	Trial steering committee
**TSF**	Trial site file
**UAR**	Unexpected adverse reaction
**UICC**	International Union Against Cancer
**UK**	United Kingdom
**WCTU**	Wales Cancer Trials Unit
**X**	Xeloda

TRIAL SCHEMA

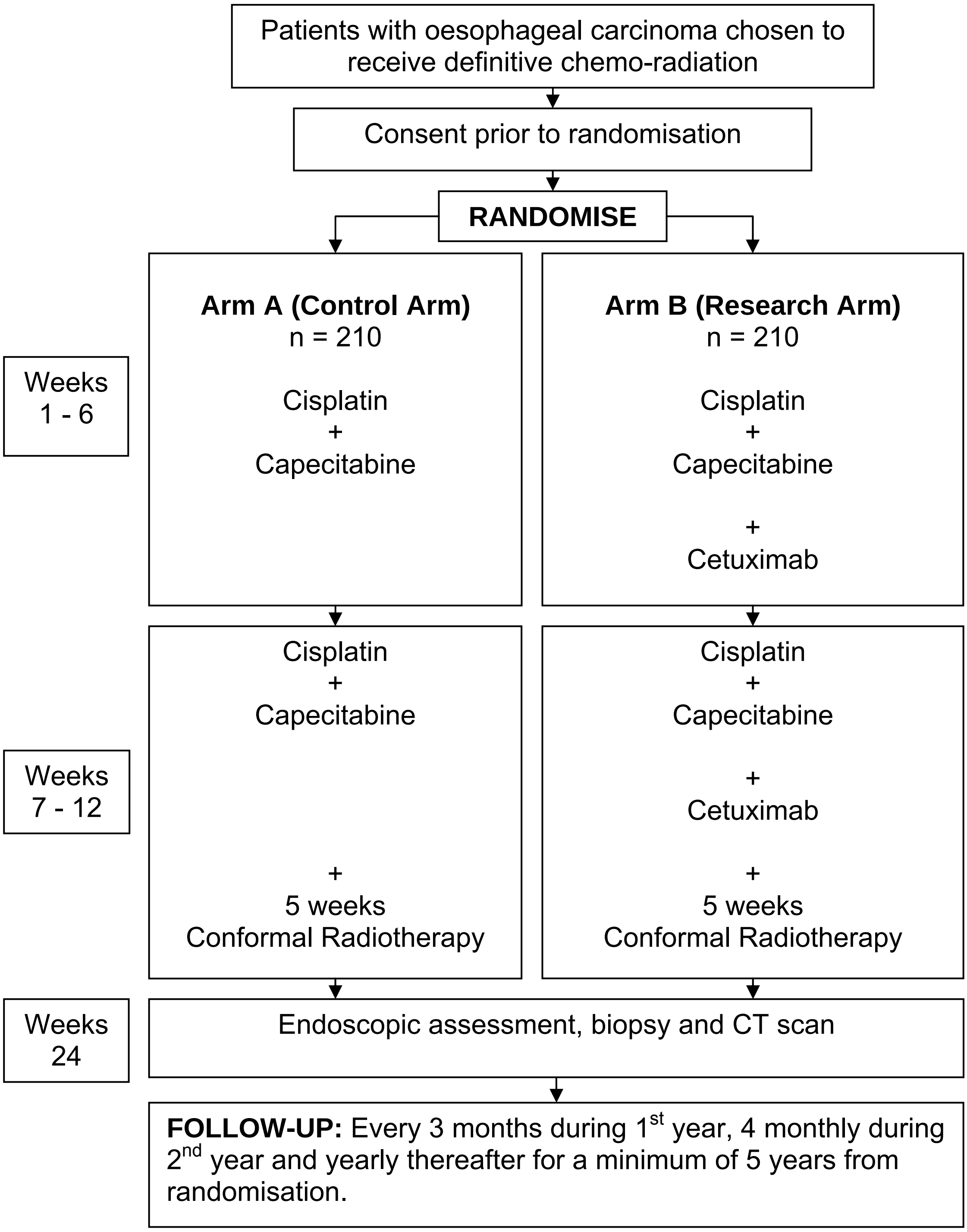

PRIMARY OUTCOME MEASURES:

- Stage 1 (after 90 patients in each arm) – toxicity, activity and feasibility
- Stage 2 (after 210 patients in each arm) – overall survival

SECONDARY OUTCOME MEASURES:

- Toxicity
- Quality Assurance
- Quality of Life
- Health Economics

TRIAL SYNOPSIS

Study Title: A randomised phase II/III multi-centre clinical trial of definitive chemo-radiation, with or without cetuximab, in carcinoma of the oesophagus.

Acronym: SCOPE 1

Short Title: Study of Chemotherapy in Oesophageal cancer Plus or minus Erbitux

**EudraCT N^{o}:**	2006-002241-37	**ISRCTN:**	47718479
**Sponsor:**	Velindre NHS Trust	**Chief Investigator:**	Dr. Tom Crosby
**Study Period:**	12 weeks treatment (Follow up over 5 years)	**Phase:**	Phase II/III

Objectives: To determine whether the addition of cetuximab to definitive chemo-radiation (CRT) shows increased survival in the treatment of patients with non-metastatic carcinoma of the oesophagus.

Number of Patients: 420 patients (Stage 1 = 180 patients; Stage 2 = 240 patients)

Diagnosis and main inclusion criteria:
Patients with non-metastatic carcinoma of the oesophagus who have been chosen for non-surgical therapy by a specialised MDT including a designated Upper GI surgeon either for clinical reasons or patient choice.

Patients meeting the following criteria can be included in the trial (full eligibility criteria listed in section 4):

- Patients older than 18 years of age suitable to receive potentially curative definitive chemo-radiation.
- Histologically confirmed carcinoma of the oesophagus (adenocarcinoma or squamous cell or undifferentiated carcinoma) or Siewert Type 1 or 2 tumour of the gastro-oesophageal junction (GOJ) with no more than 2cm mucosal extension into the stomach.

Control treatment
Cisplatin 60mg/m^2 IV Day 1 of 21 day cycle for 4 cycles
Capecitabine 625mg/m^2 po bd Days 1-84
From week 7;
Radiotherapy 50 Gray in 25 fractions over 5 weeks, 2Gy / fraction
Research arm
As above
+ Cetuximab 400mg/m^2 day 1, week 1 only
250mg/m^2 weekly thereafter for a further 11 weeks

Baseline assessments

- Endoscopic assessment and biopsy confirmation of squamous cell, adenocarcinoma or undifferentiated carcinoma of oesophagus or Siewert Type 1 or 2 tumour of the gastro-oesophageal junction (GOJ) with no more than 2cm mucosal extension into the stomach.
- Endoscopic ultrasound to include recording of proximal and distal extent of primary tumour, location of lymphadenopathy and reference point for localisation on CT planning.
- Spiral/multislice CT scan with oral contrast or water.
- Physical examination and history.
- Quality of Life and Health Economics questionnaires.
- Full blood count, serum renal, liver and bone profile (including magnesium).

Assessments during and at end of treatment

- Physical examination and history.
- Full blood count and serum renal, liver and bone profile (including magnesium).
- Toxicity assessment.
- Quality of Life and Health Economics questionnaires (specified visits).

Post treatment assessments (week 24)

- Physical examination and history.
- Endoscopic assessment and biopsy.
- CT scan thorax/abdomen.
- Toxicity assessment.
- Quality of Life and Health Economics questionnaires.

Follow up

- Patients will be seen for clinical surveillance and toxicity assessments three monthly for the first year, four monthly for the second year and yearly for a minimum of five years after treatment, after which follow up should be according to local guidelines.
- Endoscopy and/or CT scan to investigate symptoms suspicious of recurrence as clinically indicated.
- Quality of Life and Health Economics questionnaires annually.

Primary outcome of stage 1: Treatment failure rate in the research arm. This will be assessed at 24 weeks (12 weeks after completion of CRT) and is defined as residual disease pathologically confirmed by endoscopic assessment, biopsy and CT scan of thorax and abdomen. The secondary outcome measures are toxicity and feasibility.
Primary outcome of stage 2: Overall survival calculated from the date of randomisation to death from any cause. The secondary outcome measures are toxicity, quality of life, quality assurance of radiotherapy and health economics.

SUMMARY

1.1 Lay Summary

SCOPE 1 (**S**tudy of **C**hemoradiotherapy in **O**esophageal Cancer **P**lus or Minus **E**rbitux™) is a study in patients with cancer of the oesophagus for whom it has been decided the best form of treatment is chemo-radiation (treatment that involves giving radiotherapy together [or concurrently] with chemotherapy). It will attempt to answer the question of whether the addition of the monoclonal antibody cetuximab (Erbitux™) improves patient survival compared to standard chemo-radiation alone.

It is known that some cancers grow as a result of activation of a protein on the surface of the cancer cell called EGFR (Epidermal Growth Factor Receptor). It is thought that this is an important mechanism that leads to resistance of cancer cells to radiation. Cetuximab is a treatment that works by preventing activation of this (EGFR) protein. In this study all patients will receive 12 weeks of chemotherapy. Patients will also receive 5 weeks of radiotherapy during the second half of treatment.

Half of the patients will be allocated to also receive cetuximab every week for the 12 weeks of trial treatment. This allocation will depend on chance. The effects of the addition of cetuximab will be analysed to see if it is any more effective and any more toxic than chemo-radiation alone, together with the costs of the different treatment and the effects on quality of life.

1.2 Abstract and summary of trial design

SCOPE 1 is a two arm, open, randomised Phase II/III trial. Eligible patients will have histologically confirmed carcinoma of the oesophagus (squamous cell, adenocarcinoma, or undifferentiated carcinoma) and have been chosen to receive definitive chemoradiotherapy (CRT) by an accredited multidisciplinary team (MDT) including a specialist Upper GI surgeon. 420 patients will be randomised to receive definitive CRT with or without cetuximab using a 1:1 ratio (210 patients per arm).

During the first stage (Stage 1) of the study, the trial will assess toxicity (unexpected and expected), activity and feasibility in 90 patients in the experimental arm. Activity will be measured as treatment failure-free rate and feasibility as the number of protocol dose modifications and delays. If toxicity, activity and feasibility are found to be unacceptable recruitment into the trial will stop. If the experimental arm is found to be active, safe and feasible, by the Independent Data Monitoring Committee (IDMC), recruitment will continue into the second stage (Stage 2) of the study. Stage 2 of the study will recruit a further 120 patients into each arm (a total of 420 patients) and compare the overall survival of both groups. The experimental arm of chemo-radiation and cetuximab will be compared against the control arm of chemo-radiation alone. This second stage of the trial is powered to detect a survival advantage with the addition of cetuximab.

All patients randomised into Stage 1 will contribute to the Stage 2 comparison of overall survival. In addition to overall survival, Stage 2 of the study will also assess toxicity, quality of life (QoL) and health economics.

Detailed radiotherapy treatment protocols have been notably absent in recent UK Upper GI Cancer trials involving chemo-radiation, as have procedures to assess adherence to

treatment recommendations. Therefore a detailed radiotherapy protocol and quality assurance procedure will be incorporated in this trial.

2. INTRODUCTION AND RATIONALE

2.1 Background

Worldwide, oesophageal cancer is the eighth most common cancer, responsible for 462,000 new cases in 2002[1] and is the fifth highest in mortality rate among tumour sites. There are 7,500 new cases of oesophageal cancer diagnosed each year in the United Kingdom and it is responsible for approximately 5% of all cancer deaths with over 7000 people dying from oesophageal cancer in the United Kingdom in 2004[2]. There are two main histological types of oesophageal cancer, squamous cell carcinoma (SCC) and adenocarcinoma (AC). Recently there has been an increase in the numbers of adenocarcinomas of the lower oesophagus and gastro-oesophageal junction in populations of the western world[1] whilst the incidence of squamous carcinoma has fallen slightly.

Surgery has long been and remains the cornerstone for cure of oesophageal cancer and is considered for all patients with potentially resectable oesophageal cancer who are fit for surgery and have no evidence of distant disease. However, survival is poor, with only approximately 40% alive 2 years after surgery[3,4]. Rates of surgical intervention vary[5-8] but in the UK are as low as 25% of all cases[8].

2.2 Chemoradiotherapy in oesophageal cancer

In a pivotal study[9], US Intergroup RTOG-8501 randomised 121 patients with squamous cell carcinoma (SCC) or adenocarcinoma (AC) to receive CRT (4 cycles cisplatin and 5-Fluorouracil (5FU), the first 2 cycles given concurrently with 50Gy radiotherapy in 25 fractions) or radiotherapy alone (64Gy in 32 fractions). This trial, together with a subsequent systematic review[10], demonstrated a survival superiority of CRT over radiotherapy alone (1-year mortality odds ratio 0.61, 95% CI 0.31-0.89, $p<0.001$), albeit at the expense of increased toxicity. This and other reported studies[9,11-16] have been predominantly in patients with SCC and have demonstrated remarkably consistent median survival of 14-18 months and 2 year overall survival of 35-40% with CRT.

In the UK, most experience has been gained in those patients, both with SCC and AC, deemed unsuitable for surgery due to the presence of co-morbidity or extent of disease[12,17]. Despite the expected poor prognosis of this patient group of the 90 consecutive patients who were deemed inoperable by a specialist Upper GI surgeon, the median survival was 26 months (2 and 5 year survival 51% (95% CI. 41, 64%) and 26% (95% CI. 13, 52%) respectively). In this study 45 patients (50%) suffered grade 3 or 4 toxicities, mainly mucosal and haematological due to the 5FU chemotherapy, though there were no treatment related deaths.

2.3 Rational for standard chemotherapy agents

Concurrent CRT regimens have been based upon cisplatin and 5FU. Both have good single agent activity in oesophageal malignant disease and are amongst the best radio-

sensitisers in tumour models[18,19]. The regimen used most frequently in the UK involves conformal external beam radiotherapy (RT), 50 Gy in 25 fractions over 5 weeks, with 2 cycles cisplatin and 5FU given concurrently, with or without a further 2 cycles of the same chemotherapy, given as a neo-adjuvant phase. The latter, as well as delivering additional systemic therapy, allows time for careful RT planning, frequently improves the patients' dysphagia and 'debulks' the tumour prior to RT.

5FU has historically been given as a continuous infusion throughout treatment. Capecitabine (Xeloda™), an oral fluoropyrimidine, sequentially converted to 5FU via 3 enzymes located in liver and tumour tissue, mimics the effect of continuous infusional 5FU. Capecitabine has been shown to be at least as effective as infusional 5FU in advanced oesophago-gastric cancer[20] and the use of capecitabine instead of infusional 5FU during Upper GI concurrent CRT has now become standard practice in some centres e.g. Velindre Cancer Centre, Cardiff and the Royal Marsden Hospital, Sutton. In Velindre, 31 patients with oesophageal and 7 patients with gastric cancer have been treated with capecitabine based CRT (Table 1). Capecitabine is also being used concurrently with RT to treat other Upper GI tumour sites[21]. In patients with significant dysphagia capecitabine can be dissolved in warm water and swallowed or even administered via a naso-gastric tube.

Table 1. A single centre experience in the use of capecitabine instead of infusional 5FU during Upper GI concurrent CRT is shown below:

	Treatment	N	Adeno:SCC	G3/4 toxicities (%)	Dose modifications/ treatment interruptions
Oesophagus	Definitive CRT	31	18:13	9 (29)<br>G3 PPE x 3<br>G3 Mucositis x 5<br>G3 Neutropenia x 1<br>1 x DVT	4 x dose reductions<br>4 x dose delay<br>2 x treatment discontinued –PD<br>1 x treatment discontinued –IHD
Stomach	Post-operative CRT	7	7:0	2 (29)<br>G3 neutropenia x 1<br>G3 PPE x 1	

Abbreviations: PPE, Palmar-plantar erythema; DVT, deep vein thrombosis; PD, progressive disease; IHD, Ischemic Heart disease.

2.4 Cetuximab and anti-EGFR therapies

The majority of patients who relapse do so within the previously irradiated area[9,14,22]. The reported local failure rate in recent studies is 45-58% of patients treated with CRT. This may reflect the advanced nature of the disease, however factors such as tumour cell repopulation during radiation therapy have long been known to be an important mechanism of radio-resistance[23,24]. Radiotherapy stimulates tumour cell growth through activation of the EGF receptor complex causing homodimerisation of the extracellular

receptor inducing autophosphorylation of the intracellular tyrosine kinase (TK) domain[25], in turn stimulating a number of intracellular signal transduction pathways such as the ras-raf-MAPK pathway[24]. This activation sequence can be blocked by the monoclonal antibody, cetuximab, preventing radiotherapy induced growth stimulation.

Bonner *et al*[26] recently reported results of a trial in patients with locally advanced squamous cell carcinoma of the head and neck, a disease with many similarities to oesophageal cancer, which tested the benefits of the addition of cetuximab to radiotherapy. 424 patients were randomised to receive 70-76.8Gy of radiotherapy with or without cetuximab. Patients were followed up for a minimum of 2 years. The addition of the antibody was well tolerated, notably without an increase in mucositis, although 34% developed predictable and mostly manageable G3-4 acute skin reactions (compared to 18%). With the addition of cetuximab there was improved local control (47% vs 34% at 3 year, p<0.01) and overall survival (55% vs 45% at 3 years, p=0.05) with nearly a doubling in median survival (49 vs 29 months). Further studies are now being planned or underway, e.g. RTOG 0234, combining adjuvant cetuximab and chemo-radiation after surgery in patients with head and neck cancer.

In patients with metastatic colorectal cancer, cetuximab has shown significant activity in combination with chemotherapy in patients who have relapsed on the same chemotherapy regimen given alone[27]. Studies are now underway or being planned, particularly in GI cancer, testing the benefits of cetuximab in addition to chemotherapy and radiation therapy, for example in locally advanced rectal cancer (EXPERT–C, XERXES). In all of the above and other published studies, cetuximab is given independently without dose modifications to standard therapy.

Anti-EGFR therapies have been combined with chemo-radiation in the treatment of thoracic malignancies. 15 patients with stage III non-small cell lung cancer received gefitinib with 60Gy radiotherapy, given concurrently with carboplatin and escalating doses of paclitaxel. This anti-EGFR small molecular agent was well tolerated even in the cohort receiving full dose chemotherapy, with an impressive response rate of 91%[28]. RTOG 0324, a Phase II study of primary chemoradiotherapy and cetuximab in Stage IIIA/B non-small cell lung cancer completed accrual of 84 patients in June 2005[29]. An interim toxicity analysis was felt acceptable in terms of toxicity although there were 2 deaths due to treatment related pneumonitis. Grade 3 and 4 side effects were seen in 50% and 23%, respectively, principally due to haematological toxicity.

In oesophageal cancer cetuximab has been tested in combination with irinotecan, cisplatin and concurrent radiotherapy in a Phase II single centre study (NCT00165490, 02-012) and in the ongoing SWOG-SO414 phase II study. In the former study 17 of 39 planned patients had completed the protocol by the ASCO 2006 meeting. Despite significant activity, only 2/15 (13%) had a pathological complete response. Grade 3/4 toxicities seen were diarrhoea (9 patients), neutropenia (9 patients), febrile neutropenia (5 patients), anorexia (5 patients), vomiting (4 patients), fatigue (3 patients) and mucositis (1 patients). In a larger series, 37 patients with oesophago-gastric cancer underwent carboplatin AUC=2, paclitaxel 50mg/m^2, cetuximab 250mg/m^2 weekly concurrently with 50.4Gy radiotherapy. There were no Grade 4 non-haematological toxicities, 20% of patients had Grade 3 oesophagitis. Eighteen of 27 (67%) had no residual disease on endoscopic biopsy after treatment and 7 of 16 (43%) who underwent surgery had a complete pathological response[30]. Building on their pivotal

work with cetuximab and radiotherapy in head and neck cancer, Dobelbower *et al* have performed a Phase 1 study of cisplatin, infusional 5FU, erlotinib (anti-EGFR small molecule) with 50.4Gy radiotherapy in 11 patients with oesophageal cancer. There were no unexpected toxicities[31].

Assessing response to chemo-radiation, without surgically removing the oesophagus for histological examination, is notoriously difficult. Endoscopy negative rates following CRT range from 30-80% (mean 61%)[32-39]. Although not a reliable marker for pathological complete response (pCR) within the surgically resected specimen[35,39] endoscopic response is strongly correlated with long-term survival[32-34]. No other technique has been shown to be more reliable though trials are ongoing into the role of 18-FDG PET and EUS guided FNA in this setting. PET assesses metabolic activity as opposed to size as for CT. This means PET is able to determine benign from malignant lesions and is not only effective in detecting the primary tumour, but also is more sensitive in detecting small nodal and distant metastases[40]. It is likely to become more commonly used after the results of larger trials and availability of equipment. It is not clear whether the presence of the EGF receptor predicts for response to cetuximab. Although not a pre-requisite for entry into this study, this and other predictors of response will be the subject of associated translational research.

2.5 Rationale and objective

Modern regimens of CRT can lead to significant long-term survival. However the majority of patients will die of their disease, most commonly with local progression/recurrence of their tumours. Cetuximab may overcome one of the principal mechanisms of tumour radio-resistance, namely repopulation, in patients treated with CRT. There are single arm CRT plus anti-EGFR agent Phase I/II trials currently underway in patients with oesophageal cancer but there are currently no published trials of chemoradiotherapy with cetuximab in oesophageal cancer (Medline, Cancerlit), and no registered ongoing randomised trials (International Cancer Research Portfolio, PDQ). Cetuximab now has a license for treatment of patients with metastatic colorectal cancer and patients with head and neck cancer in combination with radiotherapy, therefore it is timely to assess in this disease setting.

The purpose of this research is first to determine whether the addition of cetuximab to definitive chemo-radiation (CRT) shows evidence of enhanced overall survival, shows that it is safe, and that treatment of patients with non-metastatic carcinoma of the oesophagus is feasible. If cetuximab is found to be active, safe and feasible then the trial will continue recruitment to accrue sufficient patients to establish whether the addition of cetuximab to the standard treatment improves overall survival.

3. PARTICIPATING CENTRE SELECTION

This study will be carried out at participating centres within the UK who will be required to complete a site assessment form to confirm that they have adequate resources and experience to conduct the trial. As a minimum, centres must have access to and experience with EUS and multi-slice CT scans for staging and partake in appropriate specialist Upper GI MDTs which must include a specialist Upper GI surgeon. Oncologists should have appropriate experience with concurrent CRT in Upper GI Cancer.

The following documentation must be received by the Wales Cancer Trials Unit (WCTU) in order for a centre to become an approved SCOPE 1 centre.

- Confirmation of Local Ethics approval (site specific information).
- Confirmation of Trust R&D approval.
- Signed partnership agreement between Trust R&D department and Velindre NHS Trust.
- A copy of the most recent version of the Patient Information Sheet and Consent Form on local headed paper.
- Written confirmation that the Principal Investigator (PI) will ensure that patients and carers are appropriately competent to administer the drug capecitabine.
- Confirmation from the MHRA that centres/investigators have been added to the SCOPE 1 Clinical Trial Authorisation (CTA).
- Completed Investigator statement (signed by the PI at the centre).
- Completed delegation log (signature list and delegation of responsibilities).
- Full contact details for all site personnel.
- Set of lab normal ranges from laboratory being used for analyses.
- Pass of QA test for radiotherapy planning and treatment delivery.
- Choice of 5-Fluorouracil PVI (5FU). It is strongly encouraged that all centres use oral capecitabine but in centres who do not have access to capecitabine, treatment with 5FU is acceptable if stated before randomisation and used for all patients in both arms of the trial.

Site initiation will be by attendance at regional set-up meetings and or by teleconference or site visits. Recruitment may then commence.

Once all of the documentation has been received, confirmation of centre approval will be sent by WCTU to the Principal Investigator and the Clinical Supplies department at Merck KGaA who are supplying the cetuximab.

All this documentation must be stored in the Investigator Site File (TMF) at the centre and the Trial Site File at the WCTU. The WCTU must be notified of any changes to the trial personnel and their responsibilities during the running of the trial and the respective trial files must contain this up to date information.

4. TRIAL ENTRY

4.1 Patient Entry

Eligible patients will have been diagnosed with carcinoma of the oesophagus, AC, SCC, or undifferentiated carcinoma, or Siewert Type 1 or 2 tumour of the gastro-oesophageal junction (GOJ) and selected for treatment with definitive chemo-radiation by an appropriate specialised MDT. Such patients may enter the trial if they meet all the necessary inclusion and none of the exclusion entry criteria.

Any queries about whether a patient is eligible to enter the trial should be addressed to the WCTU before randomisation. Concerns will then be discussed with the Chief Investigator.

Patients are eligible for the trial if all of the following inclusion criteria (section 4.1.1) are met and none of the exclusion criteria (section 4.1.2) apply.

Randomisation will be performed centrally by the WCTU (Section 4.3) using a 1:1 allocation ratio. Patients who satisfy all the inclusion criteria and none of the exclusion criteria will be randomised. The WCTU will then send a positive entry status to confirm that the patient has been entered into the trial.

4.1.1 Inclusion criteria

Patients meeting the following criteria may be included in the trial:

1. 18 years of age or older.
2. Have been selected to receive potentially curative definitive chemo-radiation by a specialist Upper GI MDT including a designated Upper GI surgeon.
3. Not suitable for surgery either for medical reasons or through patient choice.
4. Histologically confirmed carcinoma of the oesophagus (adenocarcinoma or squamous cell or undifferentiated carcinoma) or Siewert Type 1 tumour of the gastro-oesophageal junction (GOJ) or Siewert Type 2 with no more than 2 cm mucosal extension into the stomach.
5. Tumours staged with both endoscopic ultrasound (EUS) and spiral CT scan to be T1-4, N0-1 confirming localised, non-metastatic disease (both within 6 weeks prior to randomisation, but the most recent within 4 weeks).
6. Total disease length (primary and lymph nodes) less than 10cm defined by EUS.
7. WHO performance status 0-1 (Appendix I).
8. Adequate cardiovascular function for safe delivery of chemo-radiation in the opinion of the principal investigator.
    - Where there is clinical concern patients should have an adequate cardiac ejection fraction ≥ 40% as determined by MUGA scan or ECHO (within 4 weeks prior to randomisation).
9. Adequate respiratory function for safe delivery of chemo-radiation in the opinion of the Principal Investigator.
    - Where there is clinical concern FEV_1 ≥ 1 litre as determined by spirometry (within 4 weeks prior to randomisation).
10. Adequate bone marrow and hepatic function (within 1 week prior to randomisation)
    - Absolute neutrophil count (ANC) ≥ $1.5x10^9$/L

- White blood cell count ≥ $3x10^9$/L
- Platelets ≥ $100x10^9$/L
- Haemoglobin (Hb) ≥ 10g/dL (patients' Hb should be corrected to >10g/dl before treatment)
- Adequate liver function (within 1 week prior to randomisation)
- Serum bilirubin ≤ 1.5x ULN
- ALT / AST ≤ 2.5x ULN
- ALP ≤ 3x ULN

11. Adequate renal function (within 1 week prior to randomisation)
    - Glomerular filtration rate (GFR) assessed by EDTA clearance to be > 40mL/min (or estimated by Cockcroft-Gault formula to be > 60 mL/min) (Appendix II).
12. Patients who are fit to receive all protocol treatment.
13. Patients who are able and willing to administer capecitabine.
14. Patients who are of child bearing age are willing to use contraception.
15. Patients who have completed baseline quality of life questionnaires (Appendices III and IV).
16. Patients who have provided written informed consent prior to randomisation.

4.1.2 Exclusion criteria

If any of the following criteria apply, patients cannot be included in the trial:

1. Patients who have had previous treatment for invasive oesophageal carcinoma or gastro-oesophageal junction carcinoma (not including PDT or laser therapy for high grade dysplasia/carcinoma in-situ).
2. Patients with metastatic disease i.e. M1a or M1b according to UICC TNM version 6.
3. Patients with any previous treatment for malignancy which will compromise ability to deliver definitive mediastinal chemo-radiation or may compromise survival.
4. Patients who have had a previous malignancy during the previous 5 years.
5. Patients with significant (>2cm) extension of tumour into the stomach.
6. Patients with unstable angina or uncontrolled hypertension or cardiac failure or other clinically significant cardiac disease.
7. Patients who have had major surgery or major trauma in the 4 weeks prior to randomisation.
8. Patients who have been treated with a monoclonal antibody in the 4 weeks prior to randomisation.
9. Patients who have been treated with radiotherapy in the 3 months prior to randomisation.
10. Patients who need continued treatment with a contraindicated concomitant medication or therapy (Refer to section 6.3).
11. Patients with known dihydropyrimidine dehydrogenase (DPD) deficiency.
12. Patients with hearing impairment or sensory-motor neuropathy of WHO grade > 2 (see appendix III).
13. Women who are pregnant.

4.2 Pre-treatment procedures

Before randomisation, the following study procedures should be completed:

Patients must have histologically confirmed carcinoma of the oesophagus (adenocarcinoma or squamous cell) or Siewert Type 1 tumour of the gastro-oesophageal junction (GOJ) or Siewert Type 2 with no more than 2 cm mucosal extension into the stomach. This will have been determined by endoscopic assessment with biopsy.

Within 6 weeks prior to randomisation:

1. Spiral/multislice CT scan(+/- PET) . (Appendix IV).
2. Endoscopic ultrasound to include recording of proximal and distal extent of primary tumour, location of lymphadenopathy and reference point for localisation on CT planning (Appendix IV).

Note: The last staging assessment must have been carried out within 4 weeks prior to randomisation.

Within 1 week prior to randomisation:

1. History and physical examination (to include height, weight and WHO performance status).
2. Quality of life questionnaire (QLQ-C30) (Appendix V) + oesophageal module (QLQ-OES18) (Appendix VI) and Dermatology life quality index (DLQI)(Appendix VII).
3. Full blood count.
4. Serum renal, liver and bone profile (including serum magnesium).
5. Glomerular filtration rate - calculated creatinine clearance - Cockcroft & Gault formula (EDTA/24 hour urine collection if predicted GFR <60mls/min) (Appendix II)
6. Electrocardiogram.
7. Pregnancy test in females of child bearing age.

PET scans and bone scans may be performed according to local practice and availability and/or if clinically indicated e.g. bone pain or biochemical profile. Where these scans have been performed data will be collected in the Case Report Form (CRFs).

More detailed information on how investigations such as the endoscopic assessment, biopsy, CT scan and endoscopic ultrasound should be performed are given in Appendix IV. These are recommendations and should not replace local guidelines.

Before any study related procedures are undertaken the patient's written informed consent must be obtained. The patient should be given a minimum of 24 hours after the initial invitation to participate before being asked to sign the consent form.

4.3 Randomisation

Randomisation will be performed centrally by the WCTU. Patients will be randomised using the method of minimisation with an element of randomisation. Minimisation will ensure balanced treatment allocation by centre and a number of potential prognostic factors. Randomisation will use a 1:1 allocation ratio.

Randomisation can only be performed after the WCTU has confirmed that the patient is eligible, the patient has signed the consent form and completed a baseline Quality of Life (QoL) questionnaire. After baseline evaluation the Research Nurse or Doctor should complete the randomisation form and telephone the WCTU:

Randomisation Line:
Wales Cancer Trials Unit
Telephone 029 2064 5500
(Open Monday - Friday, 9am - 5pm)

At randomisation, the patient will be allocated to a treatment arm (chemo-radiation or chemo-radiation and cetuximab) and given a unique patient trial number. These details should be recorded on the randomisation form and the top copy returned to the WCTU. Randomisation details will be confirmed by fax by the WCTU. CRFs and QoL questionnaires will be sent to the Clinician, Data Manager or Nurse nominated as responsible for the patient.

It may be possible for patients to be enrolled in other clinical trials, but this will need to be discussed with the Chief Investigator or Co-Investigator.

5. ASSESSEMENTS

A follow up schedule is given in the following tables.

5.1 Baseline (pre-treatment)

Before any study related procedures are undertaken the patient's written informed consent must be obtained. The patient should be given a minimum of 24 hours after the initial invitation to participate before being asked to sign the consent form.

In addition to the Pre-treatment procedures (4.2) the following are to be carried out prior to treatment. The nurse/doctor will ensure the required data is recorded on the CRFs.

1. State of Health (EQ-5D) (Appendix VIII).
2. Healthcare Resource Utilisation Log.
3. Blood sample separated and stored (T-SCOPE, translational research, Section 16) (Appendix IX).
4. Dysphagia scale (Appendix X).

5.2 Assessments during and at end of treatment

1. A planning CT scan to be carried out during the first cycle of Chemotherapy (weeks 1 to 3)
2. Physical exam to include weight (weeks 1, 4, 7, 8, 9, 10, 11 and 12).
3. Toxicity assessment (weeks 1, 4, 7, 8, 9, 10, 11 and 12).
4. Full blood count (as a minimum FBC should include haemoglobin, WBC including ANC and platelets) (weeks 1, 4, 7, 8, 9, 10, 11 and 12). Haemoglobin should be maintained above 12g/dL during radiotherapy.
5. Serum renal, liver and bone profile (weeks 1, 4, 7 and 10). Urea, creatinine, sodium, potassium, magnesium, corrected calcium, albumin, bilirubin, AST and/or ALT, ALP and phosphate. (EDTA/24 hour clearance should be performed if renal function deteriorated by more than 25% (Appendix II)).
6. Quality of life QLQ-C30 + oesophageal module (QLQ-OES18) + dermatology life quality index (DLQI)(week 7, 13)
7. State of Health (EQ-5D) (week 7, 13)
8. Healthcare Resource Utilisation Log (week 4, 7, 10, 13)
9. Dysphagia scale

NB: FBC and clinical assessment (CTCAE v3.0 toxicity scores) should be performed on the day of starting each cycle (or within 3 days before) and the results available before starting.
Biochemistry (including creatinine, bilirubin and either AST or ALT) should be done at the same time and the results be available before starting the cycle. This would be considered routine clinical practice.

5.3 Post treatment (week 24)

1. Physical examination.
2. Toxicity assessment.
3. Endoscopic assessment and biopsy.

4. CT scan; thorax and abdomen (Appendix IV).
5. Quality of life QLQ-C30 + oesophageal module (QLQ-OES18) + dermatology life quality index (DLQI).
6. State of Health (EQ-5D).
7. Healthcare Resource Utilisation Log.
8. Blood sample (for T-SCOPE)(Appendix IX).

5.4 Follow up

1. Patients will be seen 3-monthly for the first year, 4 monthly for the second year and yearly for a minimum of 5 years after treatment, after which follow up should be according to local guidelines.
2. Endoscopic assessment and/or CT scans to investigate symptoms suspicious of recurrence as clinically indicated. In the case of local recurrence it will be important to ascertain the relationship of the recurrent disease to the radiotherapy field by comparing endoscopic data at recurrence with the diagnostic/staging endoscopy/EUS and/or CT data with the staging/planning CT scans.
3. Quality of life QLQ-C30 + oesophageal module (QLQ-OES18) + dermatology life quality index (DLQI) (Annually).
4. State of Health (EQ-5D) and Healthcare Resource Utilisation Log (Annually).
5. Where recurrence is diagnosed by investigations including endoscopic assessment we request a biopsy sample for translational research.

5.5 Schedule of Assessments

Month (Calendar months for study follow up)		1				2				3					6	9	12	16	20	24	36	48	60
Week	Pre-treatment	1	2	3	4	5	6	7	8	9	10	11	12	13	24								
Physical History / Exam	X	X			X			X	X	X	X	X	X	X	X	X	X	X	X	X	X	X	X
Height (week 1 only) & weight	X	X			X			X			X			X			X						
Full blood count	X	X			X			X	X	X	X	X	X	X									
WHO Performance Status	X	X			X			X			X			X	X	X	X	X	X	X	X	X	X
Dysphagia scale	X	X			X			X			X			X	X	X	X	X	X	X	X	X	X
Serum renal, liver & bone profile inc. Mg^{++}	X	X			X			X			X			X									
Electrocardiogram	X																						
Pregnancy test [A]	X																						
Quality of life QLQ-C30 + oesophageal module + dermatology life quality index	X							X						X	X		X			X	X	X	X
State of Health (EQ-5D)	X							X						X	X		X			X	X	X	X
Healthcare Resource Utilisation Log	X				X			X			X			X	X		X			X	X	X	X
Endoscopic biopsy for histological assessment [B]	X														X								
Endoscopic ultrasound [C]	X																						
Spiral / multi-slice CT scan [D]	X														X								
Blood sample (stored)	X														X								
Echocardiography / MUGA scan [E]	X																						
Spirometry [F]	X																						
Planning CT scan			X																				
Toxicity assessment	X	X			X			X	X	X	X	X	X	X	X	X	X	X	X	X	X	X	X

[A] – In females of child bearing potential.
[B] – Confirmation of squamous cell or adeno carcinoma of the oesophagus pre-treatment.
[C] – Endoscopic to include recording of proximal and distal extent of primary tumour, location of lymphandenopathy & reference point for localisation on CT planning.
[D] – Spiral / multi-slice CT scan with oral contrast or water. Minimum slice 5mm. IV contrast venous phase. CT must include thorax and abdomen.
[E] – Patients with significant history of cardiac co-morbidities that may compromise a patient's ability to tolerate definitive chemo-radiation should have Echocardiography / MUGA scan performed.
[F] – Patients with significant history of respiratory co-morbidities that may compromise a patient's ability to tolerate definitive chemo-radiation should have spirometry performed.
[G] - 5-Fluorouracil will be administered as protracted venous infusion in the event that a patient experiences problems in swallowing the capecitabine treatment.

5.6 Schedule of Treatment

Month (Calendar months for study follow up)		1				2				3					6	9	12	16	20	24	36	48	60
Week	Pre-treatment	1	2	3	4	5	6	7	8	9	10	11	12	13	24								
Cisplatin		X			X			X			X												
Capecitabine / 5-Fluorouracil PVI [G]		X	X	X	X	X	X	X	X	X	X	X	X										
Cetuximab*		X*	X*	X*	X*	X*	X*	X*	X*	X*	X*	X*	X*										
Radiotherapy								X	X	X	X	X											

* Research Arm only

6. TREATMENT REGIMEN AND SCHEDULES

6.1 Study treatments

Patients will be randomised to receive treatment on either of the following treatment arms.

Patients should start treatment within two weeks after randomisation.

Both arms will be delivered over 12 weeks.

6.1.1 Treatment summary

ARM A (Control Arm)

Day 1 to Day 42 (weeks 1-6) – Chemotherapy alone.

Cisplatin	60mg/m^2 IV Day 1 of 21 day cycle for 2 cycles.
Capecitabine*	625mg/m^2 po bd Days 1-42.

Day 43 to Day 84 (weeks 7-12) – Chemo radiotherapy.

Cisplatin	60mg/m^2 IV Day 1 of 21 day cycle for 2 cycles.
Capecitabine*	625mg/m^2 po bd Days 43-84.

Plus

Radiotherapy	50 Gray in 25 fractions over 5 weeks, 2Gy / fraction to start Day 43, Monday – Friday each field each day.

ARM B (Research Arm)

Day 1 to Day 42 (weeks 1-6) – Chemotherapy alone.

Cisplatin	60mg/m^2 IV Day 1 of 21 day cycle for 2 cycles.
Capecitabine*	625mg/m^2 po bd Days 1-42.

PLUS

Cetuximab	400mg/m^2 Day 1 cycle 1 only.<br>250mg/m^2 Day 1 each week (week 2 -6).

Day 43 to Day 84 (weeks 7-12) – Chemo radiotherapy.

Cisplatin	60mg/m^2 IV Day 1 of 21 day cycle for 2 cycles.
Capecitabine*	625mg/m^2 po bd Days 43-84.

PLUS

Cetuximab	250mg/m^2 Day 1 each week (weeks 7-12).

Plus

Radiotherapy	50 Gray in 25 fractions over 5 weeks, 2Gy / fraction to start Day 43, Monday – Friday each field each day.

* For patients who find swallowing capecitabine difficult, it is possible to dissolve the tablets in lukewarm water. The capecitabine tablets should be placed in approximately

200ml of lukewarm water. By stirring for about 15 minutes the tablets should dissolve. There is no stability data for any form of capecitabine suspension, so this should be done immediately prior to use and the solution swallowed immediately, rinsing to ensure all of the contents are ingested. As the solution will have a bitter taste it could be flavoured with a fruit juice or squash, but grapefruit juice should not be used. The solution may also be administered through a naso-gastric tube or other enteral feeding tube.

All centres should use capecitabine except in situations where patients experience problems swallowing this treatment and cannot cope with the procedure above when the following alternative treatment should be given:

Dosage: 5-Fluorouracil PVI (5FU) 200mg/m^2 IV Days 1-21 (every 3 weeks for a total of 4 cycles).

In centres who do not have access to capectabine, treatment with 5FU is acceptable but this should be stated before randomisation and used for all patients in both arms of the trial.

6.2 Treatment schedules

The following treatment schedules have been designed for ease of use and clarity. There may be some variation according to local practice. Mannitol may be given concurrently with cisplatin or may be given as a short infusion according to local policy (e.g. 100ml mannitol 20% infused over 15 minutes after sodium chloride and before cisplatin). Post chemotherapy anti-emetics should be given according to local policy.

6.2.1 Arm A: Cisplatin + capecitabine (Control Arm)

Days 1-21 of each cycle

Capecitabine 625mg/m^2 po bd taken within 30 mins of food starting in the evening of Day 1, finishing morning of Day 22. In case of dysphagia dissolve in 200ml lukewarm water and take with juice (not grapefruit) if it tastes bitter (Refer to section 6.1.1).

Day 1 of each chemotherapy cycles

T(hrs)			
T 0	Pre-meds	iv bolus dexamethasone 8 mg<br>iv bolus ondansatron 8 mg (or equivalent)	flush line with 5% dextrose or normal saline
T 0 - 1	Pre-hydration	Furosemide 40mg IV bolus or po*sodium chloride 0.9% (normal saline) 1 litre<br>+ 20mmol KCl + 10mmol $MgCl_2$	IV infusion over 1 hour
T 2 - 4	cisplatin 60mg/m^2	In sodium chloride 0.9% (normal saline) 1 litre<br>concurrently with mannitol 10% 200ml	IV infusion over 2 hours
T 4 - 6	Post-	Sodium chloride 0.9% (normal saline) 1 litre	IV infusion

	hydration	+ 20 mmol KCl + 10mmol $MgCl_2$	over 2 hours
T 6 - 7	Post-hydration**	Sodium chloride 0.9% (normal saline) 500ml + 10 mmol KCl	IV infusion over 1 hour

Patients should be advised to drink 2 litres of fluids over the next 24 hours.
* Furosemide may be used in some centres, as per their usual practice, to increase urine output. However Furosemide is a potentially ototoxic drug and thus may increase the risk of ototoxicity with cisplatin.
**This final 500mls of post-hydration may be omitted if the patient is able to drink 500ml after the cisplatin.

Day 1, Cycles 3 and 4

The first fraction of radiotherapy must be given after the cisplatin has been administered on Day 1 of chemotherapy cycles 3 and 4. Radiotherapy may be given during the post-hydration which may need to be interrupted for a short period of time.

6.2.2 Arm B: Cetuximab + cisplatin + capecitabine (Research Arm)

Days 1-21 of each cycle

Capecitabine 625mg/m^2 po bd taken within 30 mins of food starting evening of Day 1, finishing morning of Day 22. In case of dysphagia dissolve in 200mL lukewarm water and take with juice if taste bitter (not grapefruit). (Refer to section 6.1.1)

Cycle 1: Day 1 of chemotherapy cycle

T(hrs)			
T 0	Pre-meds	paracetamol 1g po<br>ranitidine 150mg po<br>iv chlorphenamine 10mg,<br>iv bolus dexamethasone 8 mg<br>iv bolus ondansetron 8 mg (or equivalent)	flush line with normal saline (not dextrose)
T 0 - 2	cetuximab 400mg/m^2	(week 1 only)	IV infusion over 2 hours
T 0 - 3	**Patients should be monitored for hypersensitivity reaction to cetuximab**		
T 2 – 3	pre-hydration	Furosemide 40mg IV bolus or po* sodium chloride 0.9% (normal saline) 1 litre + 20mmol KCl + 10mmol $MgCl_2$	IV infusion over 1 hour
T 3 – 5	cisplatin 60mg/m^2	In sodium chloride 0.9% (normal saline) 1 litre<br>concurrently with mannitol 10% 200mL	IV infusion over 2 hours
T 5 – 7	Post-hydration	Sodium chloride 0.9% (normal saline) 1 litre + 20 mmol KCl + 10mmol $MgCl_2$	IV infusion over 2 hours
T 7 – 8	Post-hydration**	Sodium chloride 0.9% (normal saline) 500mL + 10 mmol KCl	IV infusion over 1 hour

Patients should be advised to drink 2 litres of fluids over the next 24 hours.
* Furosemide may be used in some centres, as per their usual practice, to increase urine output. However Furosemide is a potentially ototoxic drug and thus may increase the risk of ototoxicity with cisplatin.
**This final 500mL of post-hydration may be omitted if the patient is able to drink 500mL after the cisplatin.

Cycles 2, 3 and 4: Day 1 of chemotherapy cycle

T(hrs)			
T 0	Pre-meds	paracetamol 1g po<br>ranitidine 150mg po<br>iv chlorphenamine 10mg,<br>iv bolus dexamethasone 8 mg<br>iv bolus ondansetron 8 mg (or equivalent)	flush line with normal saline (not dextrose)
T 0 - 1	cetuximab $250mg/m^2$	(weeks 2 to 12)	IV infusion over 1 hours
T 0 - 2	**Patients should be monitored for hypersensitivity reaction to cetuximab**		
T 1 – 2	pre-hydration	Furosemide 40mg IV bolus or po*sodium chloride 0.9% (normal saline) 1 litre<br>+ 20mmol KCl + 10mmol $MgCl_2$	IV infusion over 1 hour
T 2 – 4	cisplatin $60mg/m^2$	In sodium chloride 0.9% (normal saline) 1 litre<br>concurrently with mannitol 10% 200ml	IV infusion over 2 hours
T 4 – 6	Post-hydration	Sodium chloride 0.9% (normal saline) 1 litre<br>+ 20 mmol KCl + 10mmol $MgCl_2$	IV infusion over 2 hours
T 6 – 7	Post-hydration**	Sodium chloride 0.9% (normal saline) 500mL<br>+ 10 mmol KCl	IV infusion over 1 hour

Patients should be advised to drink 2 litres of fluids over the next 24 hours.
*Furosemide may be used in some centres, as per their usual practice, to increase urine output. However Furosemide is a potentially ototoxic drug and thus may increase the risk of ototoxicity with cisplatin.
**This final 500mls of post-hydration may be omitted if the patient is able to drink 500mls after the cisplatin.

Day 8 and 15 of each chemotherapy cycle

T(hrs)			
T 0	Pre-meds	paracetamol 1g po<br>ranitidine 150mg po<br>iv chlorphenamine 10mg,<br>iv bolus dexamethasone 8 mg	flush line with normal saline (not dextrose)
T 0 - 1	cetuximab $250mg/m^2$		IV infusion over 1 hour
T 0 - 2	**Patients should be monitored for hypersensitivity reaction to cetuximab**		
T 1			flush line with normal saline (not dextrose)

Day 1, Weeks 3 and 4
The first fraction of radiotherapy must be given after the cisplatin has been administered on Day 1 of chemotherapy cycles 3 and 4. Radiotherapy may be given during the post-hydration which may need to be interrupted for a short period of time.

6.3 Non trial treatment in the research arm

6.3.1 Medications permitted

Pre-medication with an antihistamine is required to protect the patient in the event of a hypersensitivity reaction to cetuximab.
Pre-medication with steroids such as dexamethosone, analgesic (e.g. paracetamol), anti-emetic (e.g. granisetron, ondansatron) and H2 blockers (e.g. ranitidine) are recommended prior to trial treatment.

6.3.2 Medications permitted with caution

The following drugs have been shown to possibly have some interaction with cisplatin and may require dose modification:

- Ototoxic drugs like aminoglycoside antibiotics or loop diuretics (e.g. furosemide), may increase ototoxic potential of cisplatin.
- Anti-epileptics; the serum level of phenytoin may be reduced and levels should be monitored and the dose adjusted accordingly.
- Reduce renal excretion of bleomycin and methotrexate which increases their toxicity.
- Anti-gout agents (like allopurinol, colchicine, probenecid or sulfinpyrazone).

The following drugs have been shown to possibly have some interaction with Pyrimidine analogues like capecitabine or 5FU and may require dose modification:

- Coumarin derivative anticoagulants (like warfarin) require more frequent monitoring due to altered coagulation parameters, and effects may occur up to several months after initiating capecitabine therapy.
- There can also be interactions with cytochrome P-450 (isozymes 1A2, 2C9 and 3A4).
- Allopurinol (reduce the efficacy of 5FU).
- Phenytoin plasma levels may be increased, levels should be regularly monitored.

6.3.3 Non permitted medications

Cetuximab has no known interactions with concomitant medications.

The following drugs have been shown to interact with cisplatin and should be avoided if possible:

- Cumulative nephrotoxicity may be potentiated by aminoglycoside antibiotics e.g. gentamicin. These should not be administered, if possible, simultaneously or 1-2 weeks after treatment with cisplatin.
- Cisplatin can also react with aluminium and all aluminium containing IV sets, needles, catheters and syringes should be avoided.
- The solution for infusion should not be mixed with other drugs or additives.

The following drugs have been shown to have some interaction with pyrimidine analogues like capecitabine or 5FU and should be avoided:

- Sorivudine and analogues (would require a 4-week wash out prior to entering in the trial)
- Aluminium hydroxide or magnesium hydroxide containing antacids.
- Methotrexate is not recommended with any form of chemotherapy.

7. CLINICAL CONSIDERATIONS

7.1 Pregnancy

Patients should be advised that contraception should be used where applicable to avoid the possibility of pregnancy occurring to themselves, or their partners, due to the potentially harmful effects of chemo-radiation. In the very unlikely event that a pregnancy occurs patients should be advised to report this to their treatment centre immediately. The PI is responsible for reporting this information to the WCTU immediately and the pregnancy should be followed up according to guidance by the WCTU who will apply the MHRA pharmacovigilance recommendations.

7.2 Nutritional status

Patients with oesophageal cancer may have a poor nutritional status and centres should be aware that good clinical practice would normally include a basic dietician review. Patients within the trial would be expected to receive the same nutritional support as those not included, and that where there is a clinical need for invasive enteral nutrition, such as insertion of a PEG, this is permitted, and data will be collected to confirm the rate of enteral nutritional required.

7.3 DPD deficiency

Occasionally (approximately 1-3%) a patient may have a markedly exaggerated toxicity due to reduced 5FU catabolism. If this occurs, await full recovery of toxicities. Further treatment should be carried out at a much reduced capecitabine dose (e.g. 50%). Please discuss with the Chief Investigator or one of the clinical co-investigators as required.

8. DRUG SUPPLY, DOSING, SIDE EFFECTS AND MODIFICATIONS

Chemotherapy should only be administered under the direction of Oncologists in specialist units under conditions permitting adequate monitoring and surveillance. Supportive equipment should be available to control anaphylactic reactions.

8.1 Drug Supply

8.1.1 Cisplatin and Capecitabine (5FU)

Cisplatin, capecitabine and 5FU (when used in place of capecitabine) shall be available through routine medical supplies. Patients will be asked to bring any remaining tablets to their next visit so that they can be counted for drug accountability and concordance. Patient diaries will also be used to check concordance.

8.1.2 Cetuximab

Cetuximab is not licensed for this indication and drug supply will be from stock supplied by Merck KGaA. Cetuximab is provided in ready to use vials at a concentration of 5mg/mL. Details of storage and preparation of cetuximab are available as part of the drug packaging and in cetuximab's Summary of Product Characteristics (SPC).

Details of the procedures for obtaining cetuximab within the trial will be included in the SCOPE 1 pharmacy pack which will be sent to the pharmacy department of centres participating in the trial. The pack will include record sheets for drug accountability and cetuximab order forms. Stock will arrive labelled for 'Clinical trial use only'. Further drug supplies will be co-ordinated by hospital pharmacies directly with Merck KGaA using a drug request form.

Full drug accountability will be required for the cetuximab supplied to the pharmacy at the participating centres. Pharmacists will be required to document the receipt, dispensing, return and destruction of cetuximab.

Further details on the storage and preparation of cetuximab are available in the cetuximab SPC.

8.2 Dosing and precautions

Body Surface Area (BSA) should be calculated according to local policy. If required the formula and calculator are available at http://www.halls.md/body-surface-area/refs.htm.

8.2.1 Cisplatin and capecitabine (5FU)

Cisplatin will be administered as a short intravenous infusion at 60mg/m^2 over 2 hours on treatment days with adequate pre- and post hydration and electrolyte correction (see treatment schedule). On days of concurrent chemo-radiation cisplatin should be completed before radiotherapy treatment. Patients may undergo their radiotherapy during the post-hydration following cisplatin, which may be interrupted provided it is completed afterwards.

Cisplatin 1 mg/ml Sterile Concentrate should be diluted in 1 litre of 0.9% sodium chloride injection. Cisplatin reacts with metallic aluminium to form a black precipitate of platinum. All aluminium containing IV sets, needles, catheters and syringes should be avoided. The solution for infusion should not be mixed with other drugs or additives

Capecitabine treatment is usually administered on an outpatient basis.

Oral capecitabine 625mg/m^2 twice a day (to the nearest achievable dose using 500mg and 150mg tablets). Patients will be asked to take the medication every 12 hours for example 8:00am / 9:00 am and 8:00 pm / 9:00 pm each day, within 30 minutes after eating.

If a patient vomits after taking the capecitabine tablets they should not take another dose. The next dose of capecitabine should be taken at the planned usual time.

Calculated capecitabine dose at dose level 625 mg/m^2.

Dose Level 625 mg/m^2 (twice daily)		Number of tablets administered in the morning		Number of tablets administered in the evening	
Body Surface Area (m^2)	Dose per administration (mg)	150 mg	500 mg	150 mg	500 mg
≤1.38	800	2	1	2	1
1.39 – 1.52	950	3	1	3	1
1.53 – 1.78	1000	-	2	-	2
1.79 – 1.92	1150	1	2	1	2
1.93 – 2.18	1300	2	2	2	2
≥ 2.19	1450	3	2	3	2

Calculated capecitabine dose at dose level 75%

75% dose level (twice daily)		Number of tablets in the morning		Number of tablets in the evening	
BSA	Dose per admin	150mg	500mg	150mg	500mg
≤ 1.38	650mg	1	1	1	1
1.39 – 1.52	650mg	1	1	1	1
1.53 – 1.78	750mg	5	-	5	-
1.79- 1.92	800mg	2	1	2	1
1.93 – 2.18	1000mg	-	2	-	2
≥ 2.19	1150mg	1	2	1	2

Calculated capecitabine dose at dose level 50%

50% dose level (twice daily)		Number of tablets in the morning		Number of tablets in the evening	
BSA	Dose per admin	150mg	500mg	150mg	500mg
≤ 1.38	450mg	3	-	3	-
1.39 – 1.52	500mg	-	1	-	1
1.53 – 1.78	500mg	-	1	-	1
1.79- 1.92	600mg	4	-	4	-
1.93 – 2.18	650mg	1	1	1	1
≥ 2.19	650mg	1	1	1	1

5FU

For patients unable to receive capecitabine, 5FU will be administered as protracted venous infusion via a central line at 200mg/m^2/day. Administration and anticoagulation will be according to local treatment policy.

8.2.2 Cetuximab

There are no known interactions between cetuximab and the cytotoxic agents being used. The latter should therefore be administered according to the schedule and precautions described above. The following advice applies just to the additional use of cetuximab.

Pre-treatment

Prior to the first infusion, patients on the cetuximab research arm of the study (Arm B) must receive pre-medication with an appropriate antihistamine. The antihistamine (to reduce the risk of an allergic reaction) should be administered prior to the starting dose and the subsequent weekly doses.

Cetuximab (Erbitux™) 400mg/m^2 (initial) dose shall be given as an IV infusion over 120 minutes. Subsequent weekly doses of 250mg/m^2 shall be given as an infusion over 60 minutes. The maximum infusion rate must not exceed 10 mg/min cetuximab (Erbitux™) (corresponding to 2 mL/min ready-to-use solution).

Close monitoring of the patient is required during the infusion and for at least 1 hour after the end of the infusion. Resuscitation equipment must be available. The supervising clinician should be in the room or unit during the first dose of cetuximab.

On days where patients are receiving radiotherapy, cetuximab should be given prior to radiotherapy.

Cetuximab is contraindicated in patients with known severe (grade 3 or 4) hypersensitivity reactions to cetuximab. There are no specific drug interactions documented with cetuximab, however, any agent that could interfere with a patient's immune system should be avoided. Exceptions to this include the study regime and necessary supportive treatments (including anti-emetics, corticosteroids etc).

Dosage and Administration Procedure:

Initial dose:
The total **initial dose** (first infusion) is **400 mg/m² (80 mL/m² ready-to-use solution)** and is administered over a period of 120 minutes (maximum infusion rate of 10 mg/min, corresponding to 2 mL/min ready-to-use solution). Patients must be pre-treated with an antihistamine. Observe the patient during infusion and for one hour afterwards. Check the vital signs pre-, mid-, post- and one hour post-infusion. Use a sterile 0.9% NaCl solution to flush the line at the end of infusion.

Further infusions:
The **weekly dose** (all further infusions) is **250 mg/m² (= 50 mL/m² ready-to-use solution)** and is administered over a period of 60 minutes (maximum infusion rate of 10 mg/min, corresponding to 2 mL/min ready-to-use solution). It is recommended that the patient is pre-treated with an antihistamine prior to each infusion. Observe the patient during infusion and for one hour afterwards. Check the vital signs pre-, mid-,

post- and one hour post-infusion. Use a sterile 0.9% NaCl solution to flush the line at the end of infusion.

Do not mix cetuximab solution with any intravenously administered medicinal product other than a sterile 0.9 % NaCl solution. Use a separate infusion line for cetuximab infusion. For dose reduction due to adverse events, see the section 8.4.2. **Wait at least one hour after completing the cetuximab infusion before patient receives chemotherapy.**

Preparation and Administration of the Infusion

Cetuximab solution contains no antimicrobial preservative or bacteriostatic agent. This means care must be taken to ensure aseptic handling when preparing the infusion.

There are two options:

Syringe Pump:

- Calculate the required amount of cetuximab per patient and administration (e.g., 250 mg/m² for a 2 m² patient = 500 mg cetuximab). Calculate the required amount in volume cetuximab solution at 5 mg/mL (e.g., 500 mg cetuximab = 100 mL cetuximab 5 mg/mL)
- Draw up the volume calculated above from one or several cetuximab 5 mg/mL vials, using one or several appropriate sterile syringes attached to a suitable needle.
- Remove the needle, affix the infusion line to the first filled syringe, and prime it with cetuximab.
- Put the first filled syringe into the syringe pump and set the rate. Repeat for remaining syringes.
- Monitor the infusion rate. The calculated infusion rate must not exceed the maximum infusion rate of 10 mg/min, i.e. 120 mL/h of the ready-to-use solution.
- Use a sterile 0.9% NaCl solution to flush the line at the end of infusion.

Infusion Pump or Gravity Drip:

- Calculate the required amount of cetuximab per patient and administration (e.g., 250 mg/m² for a 2 m² patient = 500 mg cetuximab). Calculate the required amount in volume cetuximab solution at 5 mg/mL (e.g., 500 mg cetuximab = 100 mL cetuximab 5 mg/mL)
- Take an infusion bag of adequate size (e.g., 250 mL) of 0.9% NaCl solution for infusion (isotonic saline for infusion).
- Draw up the volume calculated above from the NaCl bag, using an appropriate sterile syringe attached to a suitable needle. Discard the drawn up NaCl solution.
- Draw up the volume calculated above from one or several cetuximab 5 mg/mL vials, using one or several appropriate sterile syringes attached to a suitable needle.
- Fill the calculated volume of cetuximab into the NaCl infusion bag.
- Affix the infusion line and prime it with cetuximab before starting the infusion.

- Monitor the infusion rate. The calculated infusion rate must not exceed the maximum infusion rate of 10 mg/min.
- Use a sterile 0.9% NaCl solution to flush the line at the end of infusion.

A one-hour observation period is recommended after the cetuximab infusion.

For the initial dose, the recommended infusion period is 120 minutes. For subsequent weekly doses, the recommended infusion period is 60 minutes.

The maximum infusion rate must not exceed 10 mg/min (i.e., 2 mL/min of the 5 mg/mL solution, or, after dilution of 1 part cetuximab 5 mg/mL in 4 parts 0.9%-NaCl solution (1:5 dilution) 10 mL/min = 600 mL/h).

Recommended materials, compatibility and stability

Infusion sets or syringes made of polyethylene, polyurethane, polyolefine thermoplastic, polyamide glass microfibre, polypropylene and polyvinyl chloride have been tested for compatibility with cetuximab, and are recommended for use.

Cetuximab is stable, and is compatible with infusion systems made from any combination of the recommended infusion system components when administered at room temperature (up to 25°C). Preparations of cetuximab in the recommended infusion containers are chemically and physically stable for up to 48 hours at controlled room temperatures up to 25 °C. The product contains no antimicrobial agent and should be used immediately. If not used immediately, in-use storage times and conditions prior to use are the responsibility of the user. In-use storage at 2-8 °C should not exceed 24 hours, unless preparation has taken place under controlled and validated aseptic conditions. Discard any unused portion of the vial.

8.3 Possible side Effects

8.3.1 Cisplatin and Capecitabine (or 5FU)

Haematological toxicity: Myelosuppression is uncommonly observed with cisplatin and capecitabine (or 5FU). Neutropaenia and thrombocytopaenia should be monitored according to the recommended protocol and appropriate dose modifications made. Anaemia may occur cumulatively with cisplatin and should be corrected during radiotherapy to maintain the haemoglobin > 12g/dL.

Nephrotoxicity: Renal toxicity has been noted in about one third of patients given a single dose of cisplatin when prior hydration has not been employed. Renal toxicity becomes more prolonged and severe with repeated courses of the drug. Serum renal function should be used to estimate renal function as a baseline prior to and during treatment using the Cockroft & Gault formula (Appendix II). An EDTA clearance should be performed where indicated in the protocol and an appropriate dose adjustment made to the chemotherapeutic agents.

Gastrointestinal toxicity: Nausea and vomiting is common following cisplatin, usually starting within 1 hour of treatment and lasting up to 24 hours. Anorexia, nausea and occasional vomiting may persist for up to one week. Nausea occurs less commonly with capecitabine (or 5FU).
Diarrhoea occurs with capecitabine (or 5FU) and patients should receive advice regarding discontinuation of therapy and use of loperamide or codeine phosphate. Clinicians should be aware of infective causes of diarrhea (e.g. *Clostridium difficile)*, and patients should be tested in cases of concern. Antibiotic treatment is not recommended routinely but may be required in such circumstances. Stomatitis occurs with capecitabine and patients should receive advice regarding good oral care, and the use of mouthwash (e.g. Corsodyl™).

Hand-foot syndrome (Palmar-plantar erythrodysaesthesia/PPE): Occurs with capecitabine (or 5FU) and is reversible, where necessary with dose delay and reduction (see below). Other side effects include dermatitis, pigmentation and nail changes.

Cardiac: An infrequent association with capecitabine (or 5FU) is acute chest pain that appears to be related to coronary artery spasm and occurs more frequently in patients with known ischaemic heart disease. Fluoropyrimidines are also associated with tachyarrhythmias and ECG changes.

Neurotoxicity: Cisplatin neurotoxicity is characterised by peripheral neuropathies and paraesthesia in both upper and lower extremities and while reversible, may take a year or more to recover. Losses of taste and seizures have also been reported. This is quite different to 5FU associated neurotoxicity characterised by a transient reversible cerebellar syndrome.

Ototoxicity: Ototoxicity is cumulative with cisplatin and is not reversible. Unilateral or bilateral tinnitus, which is usually reversible, and/or hearing loss in the high frequency range, may occur. These abnormalities usually appear within 4 days after drug administration. Patients with significant neuro- and ototoxicity are not eligible and where

necessary patients should be changed to carboplatin according to the protocol (8.4 non-haematological toxicities).

Anaphylaxis: Reactions to cisplatin therapy have been occasionally reported in patients who were previously exposed to cisplatin. Patients who are particularly at risk are those with a prior history or family history of atopy. Serious reactions may be controlled by IV adrenaline, corticosteroids or antihistamines.

Serum Electrolyte Disturbances: Hypomagnesaemia, hypocalcaemia, hyponatraemia, hypokalaemia and hypophosphataemia have been reported to occur in patients treated with cisplatin and hypomagnesemia may occur with cetuximab and should be monitored according to the protocol.

Other Toxicities: Hair loss is not expected with this combination but may rarely occur with most chemotherapeutic agents. Vascular toxicities coincident with the use of cisplatin in combination with other antineoplastic agents have been reported rarely. These events may include myocardial infarction, cerebrovascular accident, thrombotic microangiopathy (haemolytic uraemic syndrome) or cerebral arteritis. There have been reports of optic neuritis, papilloedema and cerebral blindness following treatment with cisplatin.

8.3.2 Cetuximab

In approximately 5% of patients, hypersensitivity reactions may occur during treatment with cetuximab; about half of these reactions are severe.

The most commonly reported side effect with cetuximab is an acne-like skin rash, reported in approximately 80% of the patients. Less frequently skin reactions such as pruritus, dry skin, desquamation, hypertrichosis, or nail disorders (e.g. paronychia) can occur. Approximately 15% of the skin reactions are severe. The skin reactions generally resolve, without sequelae, over time following cessation of treatment if the recommended adjustments in dose regimen are followed, though occasionally may require the use of topical or even systemic antibiotics.

Mild to moderate infusion-related reactions can occur with cetuximab infusion; symptoms such as fever, chills, nausea, vomiting, headache, dizziness, or dyspnoea are seen. Severe infusion-related reactions may also occur, usually developing during or within 1 hour following the initial cetuximab infusion. These include rapid onset of airway obstruction, urticaria, hypotension, loss of consciousness and/or angina.

Cetuximab use has been associated with hypomagnasaemia (associated with lethargy and paraesthesia), possibly related to an effect on renal reabsorption, and it is therefore recommended to monitor serum magnesium levels, particularly when used with cisplatin, and replacement therapy given as required.

Dyspnoea has been reported in up to 25% of patients and conjunctivitis may be expected in approximately 5% of patients.

8.4 Dose modifications

8.4.1 Cisplatin and capecitabine (or 5FU)

Haematological

The FBC should be taken and reviewed (up to 3 days) prior to Day 1 of each cycle of chemotherapy.

**Neutrophil / platelet count (10^9/L) (day 1)**	**Action**
ANC ≥ 1 and/or plts ≥ 75	Full dose drugs
ANC 0.5- <1 and/or plts 50- <75 OR any episode of neutropenic sepsis during the previous cycle	Stop chemotherapy until recovery.<br>Restart with 25% dose reduction cisplatin and capecitabine (or 5FU)
ANC <0.5 and/or plts <50	Stop chemotherapy until recovery.<br>Restart with 50% dose reduction cisplatin and capecitabine (or 5FU)

Nephrotoxicity

Cisplatin produces cumulative nephrotoxicity. If a baseline estimate of renal function using the Cockroft & Gault formula predicts the GFR to be ≥ 60mL/min full dose cisplatin should be used. If the estimate is <60mls/min an EDTA Creatinine clearance should be performed and the appropriate cisplatin dose used (see table below). In the case of a 25% deterioration in estimated renal function (using the Cockroft & Gault formula) on pre-treatment blood samples an EDTA Creatinine clearance should be performed and pending this an appropriate dose reduction in cisplatin should be made. The EDTA Creatinine clearance result, when available, takes precedent over estimated GFR for subsequent cisplatin dose calculations.

**GFR (mls/min) Baseline and prior to Day 1**	**Action**
≥ 60ml/min	Continue full dose
50-59 ml/min	Cisplatin 50% dose
40-49ml/min	Cisplatin 50% dose<br>Capecitabine 75% dose
30-39 ml/min	Stop cisplatin, use carboplatin AUC 5<br>Capecitabine 50% dose
< 30ml/min	Stop cisplatin, use carboplatin AUC 5<br>Stop capecitabine

Consider dose reduction of 5FU in cases of severe renal impairment (i.e. GFR ≤ 10ml/min.

Neurotoxicity

Neurotoxicity/ototoxicity appears to be cumulative. Prior to each course, any new or progressive symptoms of peripheral neuropathy should be established. In the occurrence of ≥ Grade 2 ototoxicity and/or other neurotoxicity cisplatin should be replaced by carboplatin AUC 5.

Capecitabine (or 5FU) dose non-haematological modifications

Toxicity due to capecitabine administration may be managed by symptomatic treatment and/or modification of the dose (treatment interruption or dose reduction). Once the dose has been reduced, it should not be increased at a later time. Patients taking capecitabine should be informed of the need to interrupt treatment immediately if moderate or worse toxicity occurs. Doses of capecitabine omitted for toxicity are not replaced or restored, instead patients should resume the planned treatment cycle. The following are the recommended dose modifications for toxicity. In addition patients should receive loperamide in case of diarrhoea, pyridoxine for PPE and mouthwashes and anti-emetics according to local policy

Toxicities should be graded according to CTCAEv3.0. An abridged version is included in Appendix XI for use when completing CRFs. In particular, diarrhoea, nausea, vomiting, stomatitis and skin reactions are to be noted.

Please use alternative specific toxicity for Hand-foot syndrome (PPE), the frequency of which in patients receiving capecitabine has led to altered toxicity ratings.

Grade of hand-foot syndrome	
1	Numbness, dysaesthesia/paraesthesia, tingling, painless swelling or erythema of the hands and/or feet and/or discomfort which does not disrupt the patient's normal activities.
2	Painful erythema and swelling of the hands and/or feet and/or discomfort affecting the patient's activities of daily living.
3	Moist desquamation, ulceration, blistering and severe pain of the hands and/or feet and/or severe discomfort that causes the patient to be unable to work or perform activities of daily living.

If grade 2 or 3 hand-foot syndrome occurs, administration of capecitabine should be interrupted until the event resolves or decreases in intensity to grade 1. Following grade 3 hand-foot syndrome, subsequent doses of capecitabine should be decreased.

Capecitabine (or 5FU) dose reduction schedule for non-haematological toxicities.

Toxicity	During a course of therapy	Dose adjustment for next cycle (% of starting dose)
• Grade 1		
	Maintain dose level	Maintain dose level
• Grade 2		
1st appearance	Interrupt until resolved to grade 0-1	100%
2nd appearance	Interrupt until resolved to grade 0-1	75%
3rd appearance	Interrupt until resolved to grade 0-1	50%
4th appearance	Discontinue treatment permanently	
• Grade 3		
1st appearance	Interrupt until resolved to grade 0-1	75%
2nd appearance	Interrupt until resolved to grade 0-1	50%
3rd appearance	Discontinue treatment permanently	
• Grade 4		
1st appearance	Discontinue permanently *Or* If physician deems it to be in the patient's best interest to continue, interrupt until resolved to grade 0-1 after discussion with Chief Investigator	50%

Chest pain
If unexplained chest pain occurs on treatment, capecitabine (or 5FU) should be stopped, an ECG performed and cardiac enzymes measured. In the case of angina or myocardial infarction being confirmed this should be managed according to usual local practice. Patients should not recommence capecitabine (or 5FU) therapy and further therapy should be discussed with the Chief Investigator. Such cardiac toxicity should be reported through a SAE form.

8.4.2 Cetuximab dose modifications / criteria for stopping

Allergic/Hypersensitivity reactions	Action
Grade 1<br>Transient flushing or rash, drug fever <38°C	Decrease the cetuximab infusion rate by 50% and monitor closely for any worsening.<br>Cetuximab infusion time can be increased to a maximum of 4 hours; the infusion time will remain prolonged for subsequent infusions.<br><br>If patient has a second reaction on this prolonged infusion rate, the infusion should be stopped and the treating clinician should consider withdrawing the patient from the study.
Grade 2<br>Rash; flushing; urticaria; dyspnoea; drug fever ≥ 38°C	Stop cetuximab infusion.<br>Administer bronchodilators, oxygen etc. as medically indicated.<br>Resume infusion at 50% of previous rate once allergic/hypersensitivity reaction has resolved or decreased to grade 1 in severity, and monitor closely for any worsening.<br>Cetuximab infusion time can be increased to a maximum of 4 hours; the infusion time will remain prolonged for subsequent infusions.<br><br>If patient has a second reaction on this prolonged infusion rate, the infusion should be stopped and not reintroduced.<br>This second reaction must be reported as a serious adverse event.
Grade 3: Symptomatic bronchospasm, with or without urticaria; parenteral medication(s) indicated; allergy-related oedema/angioedema; hypotension	Stop cetuximab infusion immediately and do not reintroduce.<br><br>Administer adrenaline, bronchodilators, antihistamines, glucocorticoids, intravenous fluids, vasopressor agents, oxygen etc., as medically indicated.<br><br>This must be reported as a serious adverse event.
Grade 4: Anaphylaxis	Patients have to be withdrawn immediately from treatment and must not receive any further cetuximab treatment.<br><br>This must be reported as a serious adverse event.

Cetuximab skin toxicity

Patients presenting with grade 1 acneiform skin rash should be considered for concomitant treatment with topical and oral antibiotics. Systemic antibiotics should be considered for grade 2 acneiform eruptions and are mandatory for grade 3 and above reactions.

Grade	1	2	3	4
Rash: acne/acneiform	Intervention not indicated	Intervention indicated	Associated with pain, disfigurement, ulceration, or desquamation	-

From NCI CTCAE v3

Cetuximab dose modifications for skin toxicity

Grade 1 and 2: Continue cetuximab for all occurrences
Grade 3: Dose delay and reduction as detailed in diagram below
Grade 4: Discontinue cetuximab (see below)

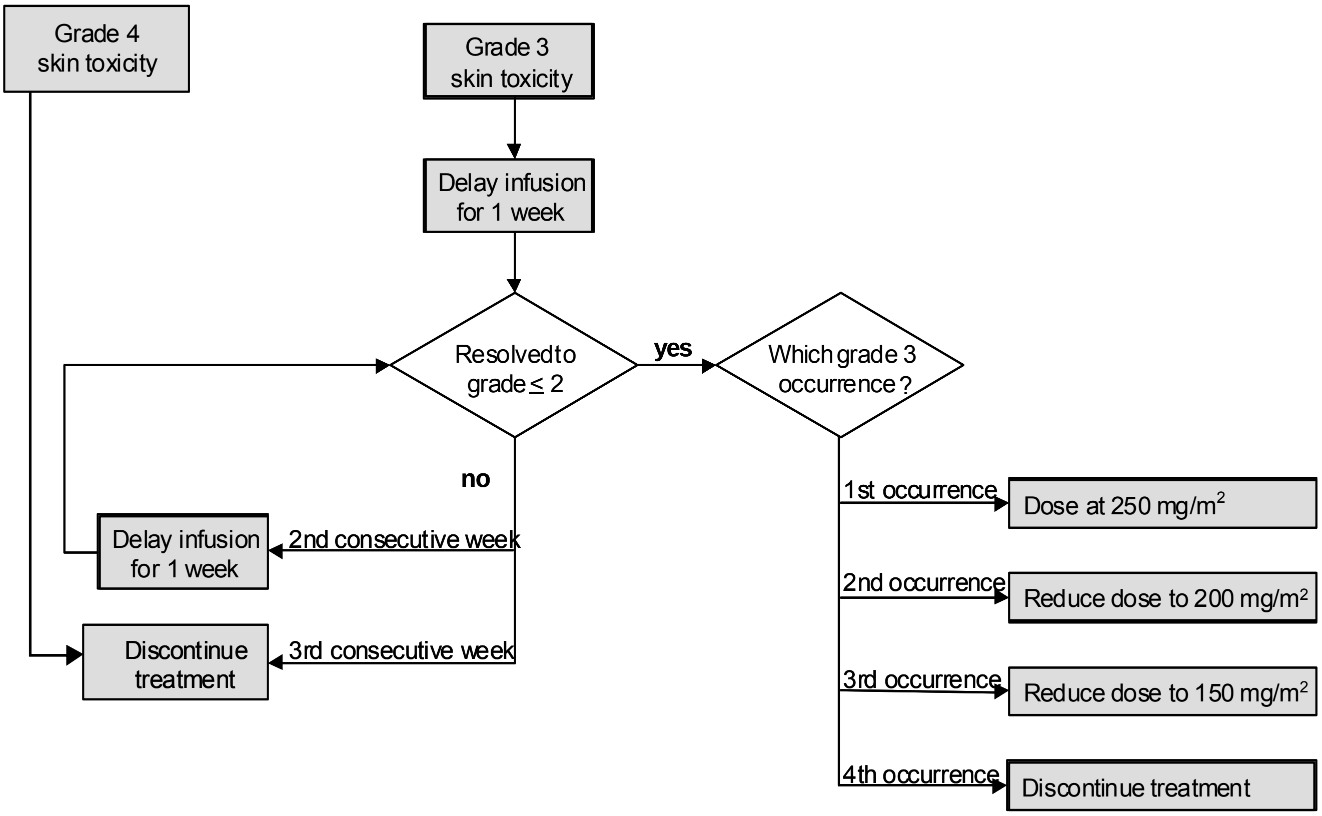

9. RADIOTHERAPY

The detailed radiotherapy guidelines and procedures (separate document) and quality assurance procedures (section 17) have been put in place to ensure protocol compliance as these are a very important aspect of this trial. The separate radiotherapy document for this trial gives detailed information on oesophageal planning including guidance on plan optimisation. A CD ROM will be sent to centres wishing to participate in this study which will include a Practical Guide to Oesophageal Planning using the technique described below with three example cases. A test case must be planned by each Investigator supervising patients in this trial before recruitment can start.

The radiotherapy schedule is the same for both arms (50Gy in 25 fractions over 5 weeks). It is highly recommended that the Radiotherapy will be delivered in a single phase, treating each field daily Monday to Friday and prescribed to the ICRU 50 reference point, usually the point of intersection of the central axes. Conformal radiotherapy with a pixel based inhomogeneity correction is essential. Photon energy should be between 6MV and 10MV (energies in excess of 10MV should only be used in exceptional cases due to secondary build-up depth.

Patients should be planned and treated in the supine position with their arms above their heads. The planning CT scan should be performed within 2 weeks of starting the neoadjuvant phase of chemotherapy AND within 8 weeks after the staging spiral CT scan. The planning CT scan should be performed according to local practice but should be at 3-5mm slices. To enable accurate assessment of the doses to organs at risk (OAR) the scan should extend superiorly to at least one CT slice above the apices of the lungs and inferiorly to the iliac crest (L2). Scans for upper third tumours may need to extend superiorly to the tragus. It is helpful to use intravenous contrast to help distinguish the GTV from surrounding tissues; oral contrast is NOT helpful in most cases and it may interfere with planning calculations. In most cases the GTV (extent of primary and nodal disease) is defined by the EUS taking into account information from the diagnostic spiral CT scan, barium studies and 18-FDG PET scan if performed.

9.1 Definition of Treatment Volumes

Targets are defined following the principles of ICRU 50 and 62. The spinal cord and spinal cord PRV (section 3.2 in the ‘radiotherapy guidelines and procedures’ document) should be outlined prior to definition of the treatment volumes. The target volumes are localised on axial slices of the planning CT scan.

9.1.1 Gross Tumour Volume

Gross Tumour Volume (GTV) is the gross primary and nodal disease as defined on the planning-CT scan with all available diagnostic information. This should include EUS as a minimum. In principal the GTV should encompass disease defined on any of the available imaging modalities used (i.e. CT, EUS and/or PET), even if only apparent on a single modality. The EUS GTV is used to define the longitudinal margins with the aid of an EUS derived reference point i.e. arch of aorta or tracheal carina which is easily seen on CT axial images e.g. if the carina is 26cm ab oral (distance to incisors) on EUS and

the proximal tumour extent is at 28cm, this would be 4 x 5mm CT slices below the carina as seen on the CT. However, one should encompass 'tumour' seen on the CT plan even if outside the EUS defined disease extent i.e. the GTV should be the most proximal and distal extension of disease as seen on EUS or CT scan. The lateral and anterior-posterior GTV margins are derived from the planning CT scan.

9.1.2 Clinical Target Volume

The CTV is defined differently in two clinical situations depending on the proximity of the GTV to the gastro-oesophageal junction (GOJ). Tumours that have a high risk of disease within the stomach have the potential to metastasise to lymph nodes that will only be included within the CTV if specifically outlined. Thus, two CTV definition protocols are defined in the Radiotherapy guidelines and procedures document.

Therefore the clinical target volume is:

Superiorly and inferiorly	The EUS defined extent of tumour (primary or nodal) with a 2cm margin along the axis of the oesophagus (see above)
Laterally and anteriorly	1cm margin around the tumour defined by the planning CT scan
Posteriorly	0.5-1cm cm margin around tumour defined by the planning CT scan

9.1.3 Planning Target Volume

The PTV is created by the addition of the following margins:

Superiorly and inferiorly	CTV + 1cm<br>i.e. geometrically superior-inferiorly
Laterally and anteriorly/posteriorly	CTV + 0.5cm (this margin is applied in all circumstances regardless of the proximity of the target to the spinal cord)

The maximum treatment field length is 17cm, i.e. maximum EUS disease length (primary tumour and lymph nodes 10cm assuming 1cm extension from PTV to field length).

9.1.4 Lower 1/3rd tumours

If the tumour involves the GO junction, i.e. Siewert Type 1, the inferior margins to define the PTV should also be 3 cm below the EUS defined GTV. However the inferior volume should be grown as described in the 'radiotherapy guidelines and procedures' (separate document) to cover the lymph node stations along the lesser curve to include the para-cardial, and left gastric lymph nodes along the lesser curve of the stomach as seen on the CT scan.

9.1.5 PTV coverage

The guidelines for the PTV should be taken as definitive (subject to the type of dose calculation algorithm used). 95% of the PTV volume should receive greater than 99% of the dose and the minimum dose to any part of the PTV should be greater than 93%.

The PTV min should be no less than 93% and the PTV max should be no more than 107% of the dose prescribed to the ICRU 50 reference point.

9.2 Organs at Risk

The full extent of the right and left lungs are outlined, this should be done in such a way that the planning system will be able to calculate a combined lung dose volume histogram (DVH).

The spinal cord should be outlined on slices which include or are within 2cm of the PTV in the superior and inferior directions. A Planning Risk Volume (PRV) for the cord is created to account for positioning error, the size of the margin added to the cord being commensurate with the accuracy of treatment delivery expected and as such the tolerance level allowed in portal image verification on treatment.

The whole heart is outlined to the extent of the pericardial sac (if visible). The major blood vessels (superior to the organ) and the inferior vena cava (towards the inferior extent of the heart) are excluded.

The whole liver is outlined if the level of its superior edge overlaps with the level of the inferior extent of the PTV.

Each kidney is outlined separately if the level of its superior edge overlaps with the level of the inferior extent of the PTV.

9.3 Normal Tissue Tolerance

The following normal tissue tolerance doses should be observed and recorded for each patient plan;

Spinal cord	The spinal cord (with a margin of 5mm) should receive less than 40Gy i.e. D40=0%
Heart	less than 30% of the total volume of heart should receive more than 40Gy i.e. V40 < 30%
Lung	less than 25% of the total lung volume should receive more than 20Gy i.e. V20 < 25%

9.4 Treatment delivery

All treatment will be delivered in a single 3D (conformal) CT planned phase i.e. treatment cannot start following conventional simulation. Given the target volume described above and the normal tissue dose constraints below, it is up to individual

participating centres to decide the field arrangements. It is recommended however that a 4-field technique is usually satisfactory, with anterior-posterior parallel opposed and two posterior oblique or lateral fields. Additional fields using gantry orientations as above may improve dose homogeneity across the PTV. The treatment should be delivered with a single phase technique.

The plan should be verified in the simulator, or equivalent, prior to starting treatment and at least one portal image taken in the first week of treatment on the linear accelerator and weekly throughout treatment thereafter.

9.5 Treatment Gaps

Gaps in treatment should be managed in both arms according to usual centre practice. Squamous cell carcinomas should be Category 1, i.e. unscheduled breaks in treatment should not lead to prolonged overall treatment time where possible e.g. by delivering 2 fractions per day (more than 6 hours apart).Where possible during the concurrent CRT phase patients should continue with RT even if unable to tolerate CT. Patients should be withdrawn from trial treatment if RT is delayed greater than 2 weeks (Section 10.8.1).

10. TRIAL CONDUCT AND MONITORING

10.1 Measures of concordance / adherence

Compliance checks for all trial medications will be by confirmation in the CRF. The capecitabine concordance will be checked by tablet counts and reference to patient diary cards.

10.2 Data Handling

The top copy of each completed CRF should be returned to WCTU for data entry within 4 weeks of the visit. The remaining copy is to be retained at the local centre.

The WCTU staff will be in regular contact with local centre personnel to check on progress and to help with any queries that may arise. Incoming forms will be checked for completeness, consistency, timeliness and compliance with the protocol. Centres may be withdrawn from further recruitment in the event of serious and persistent non-compliance. Data will be handled and stored in accordance with the Data Protection Act (1998).

10.3 Loss to follow-up

If a patient is lost to follow-up the WCTU will request the centre contact the patient's GP to obtain information on the patient's status. If needed and the patient has given the necessary consent, they will be traced via the National Health Service Information Service (NHS IC) or the NHS Central Register.

10.4 Central monitoring

Case report form pages and data received by WCTU from participating trial sites will be checked for missing, illegible or unusual values (range checks) and consistency over time. If missing or questionable data are identified, a data query will be raised on a data clarification form. The data clarification form will be sent to the relevant participating site. The site shall be requested to answer the data query or correct data on the data clarification form. The case report form pages should not be altered. All answered data queries and corrections should be signed off and dated by a delegated member of staff at the relevant participating site. The completed data clarification form should be returned to the WCTU and a photocopied version retained at the site along with the relevant case report form. The WCTU shall send reminders for any overdue data.

10.5 Direct access to data

Investigators should agree to allow trial related monitoring, including audits and regulatory inspections by providing direct access to source data/documents as required. Patient consent for this will be obtained.

10.6 Quality assurance and quality control of data

A quality assurance program will be in place to ensure adherence to the protocol. Major and minor deviations will be collected.

A monitoring Standard Operating Procedure (SOP) will be developed for the trial. We aim to review trial documentation and perform source data verification of at least 10% of patients during the course of the trial. Any centre may be selected for monitoring and we will aim to monitor at least 3 patients or 10% of patients at each centre (whichever is greater).

The NCRI Radiotherapy Clinical Studies Group have worked with the trial management group (TMG) to put in place appropriate radiotherapy quality assurance procedures.

10.7 End of trial

For the purpose of complying with UK Medicines for Human Use (Clinical Trial) Regulations (SI 2004/1021) introduced in May 2004, the end of the trial will be when the last patient has completed protocol treatment and the week 24 assessment which includes Endoscopic assessment and CT scan.

For the purposes of the Research Ethics Committee approval, the study end date is deemed to be the date of last capture. For this trial this includes follow-up of all patients for a minimum of 5 years. This will be done via the hospital but in the longer term this may be carried out via the National Health Service Information Centre (NHS IC) or NHS Central Register.

10.8 Patient withdrawal

In consenting to the trial, patients are consenting to cetuximab (if allocated), trial follow up and data collection. If a patient wishes to withdraw from cetuximab, participating centres should nevertheless explain the importance of remaining on trial follow up. If the patient explicitly states their wish not to contribute further data to the trial, the research nurse should inform the WCTU in writing.

10.8.1 Withdrawal from trial treatment

A patient may withdraw, or be withdrawn, from the treatment protocol for the following reasons:

a) Intolerable adverse effects as judged by the Investigator or the patient.
b) Patient decision to discontinue treatment.
c) Any patient whose treatment is delayed for longer than 3 weeks due to drug treatment related toxicity should discontinue therapy. Treatment delay for radiotherapy should be not be longer than 2 weeks.
d) Serious systemic allergic reaction to any of the study drugs e.g. angio-oedema, anaphylaxis.

The reason should be recorded on the treatment form and the withdrawal form.

In such cases patients will be clinically managed as deemed appropriate by their supervising clinician, but such treatment would not be expected to include cetuximab which is considered as an experimental treatment in this disease.

The patient should however remain in the trial for the purposes of follow up and data analysis and follow up CRFs should be returned to the WCTU.

10.8.2 Withdrawal from trial completely

If a patient explicitly withdraws consent to have any data recorded, their decision must be respected and recorded on the withdrawal form. Details of the withdrawal form should be noted in the patient records and no further SCOPE 1 CRFs should be completed for the patient.

11. SAFETY REPORTING

The Medicines for Human Use (Clinical Trials) Regulations 2004 apply for this protocol:

Adverse Event (AE): any untoward medical occurrence in a subject to whom a medicinal product has been administered, including occurrences which are not necessarily caused by or related to that product.

Adverse Reaction (AR): any untoward and unintended response in a subject to an investigational medicinal product which is related to any dose administered to that subject.

Serious Adverse Event (SAE): Any adverse event that

- results in death
- is life-threatening*
- requires hospitalisation or prolongation of existing hospitalisation**
- results in persistent or significant disability or incapacity
- is a congenital anomaly or birth defect
- Other important medical events***

Comments

*Life threatening in the definition of a serious adverse event or serious adverse reaction refers to an event in which the subject was at risk of death at the time of event; it does not refer to an event which hypothetically might have caused death if it were more severe

**Hospitalisation is defined as an inpatient admission, regardless of the length of stay, even if the hospitalisation is a precautionary measure, for continued observation. Pre-planned hospitalisation e.g. for pre-existing conditions which have not worsened or elective procedures does not constitute an adverse event.

***Other events that may not result in death, are not life threatening, or do not require hospitalisation may be considered as a serious adverse experience when, based upon appropriate medical judgment, the event may jeopardise the patient and may require medical or surgical intervention to prevent one of the outcomes listed above (excluding new cancers or result of overdose).

Serious adverse reactions (SARs) are SAEs which are considered by the investigator to be possibly/probably/definitely related to the trial treatment. Most SARs are expected.

Serious Adverse Reactions (SARs): SARs are **SAEs** which are considered by the investigator to be possibly/probably/definitely related to the trial treatment.

Suspected Unexpected Serious Adverse Reactions (SUSARs): These are **SARs** which are classified as “unexpected” i.e. an adverse reaction the nature and severity of which is not consistent with the information about the medicinal product in question set

out in the summary of product characteristics (SPC) and Investigators Brochure (IB) for that product. A copy of the SPCs and IB (cetuximab) for the investigational medicinal products has been sent to sites as part of the investigator site file. If an adverse reaction is more severe than expected or it is thought that it is exacerbated by the study treatments, this must be considered in the assessment of the event.

Expected side effects from protocol treatment (listed in alphabetical order, not the order of importance).

Cetuximab	Capecitabine	Cisplatin	RT
Acne-like rash (see table and diagram on p45 of this of protocol)<br>Allergic reaction<br>Conjunctivitis<br>Dermatitis<br>Dyspnoea<br>Dysphagia<br>Fatigue<br>Hypersensitivity<br>Hypokalaemia<br>Hypomagnasaemia<br>Oesphagitis<br>Rash<br>Stomatitis<br>Urticaria	Abdominal pain<br>Alopecia<br>ALT / AST changes<br>Anorexia<br>Bilirubin changes<br>Cardiotoxicity<br>Cardiac ischaemia / infarction<br>Central/peripheral nervous system<br>Creatinine changes<br>Dermatitis<br>Diarrhoea<br>Disease<br>Fatigue<br>Febrile neutropenia<br>Hand foot syndrome<br>Hypocalcaemia<br>Hypercalcaemia<br>Infection with normal ANC<br>Injection site reaction<br>Leukocytes<br>Myelosuppression<br>Nail changes<br>Nausea<br>Neutrophils (ANC)<br>Oesphagitis<br>Platelets<br>Stomatitis	Anaphylactic-like<br>Neurotoxicity<br>Alopecia<br>ALT / AST changes<br>Anoriexia<br>Bilirubin changes<br>Creatinine changes<br>Fatigue<br>Febrile neutropenia<br>Haemoglobin<br>Hearing<br>Hypocalcaemia<br>Hypouricemia<br>Infection with normal ANC<br>Injection site reaction<br>Leukocytes<br>Nephrotoxicity<br>Neutrophils (ANC)<br>Neuropathy – motor<br>Neuropathy – sensory<br>Nausea<br>Oesphagitis<br>Ototoxicity<br>Platelets<br>Tinitus<br>Thrombosis/Thrombus	Acute gastritis<br>Anorexia<br>Chronic renal damage<br>Diarrhoea<br>Dermatitis<br>Dysphagia<br>Fatigue<br>Gastro-intestinal perforation<br>Gastro-intestinal bleeding<br>Nausea and vomiting<br>Oesphagitis<br>Radiation dermatitis<br>Skin discolouration<br>Second malignancy<br>Subacute intestinal obstruction<br>Weight loss

For all adverse reactions:

- Grade 1-2: Reported only on CRF Toxicity form
- Grade 3-5*: Reported on CRF Toxicity form and on SAE form as SAR (*where Grade 5 is death)

Please note that although this list was exhaustive at the time of authorisation of this protocol, the SPC may have been updated and we encourage you to consult the most

recent version for the treatments in question when assessing the expectedness of an adverse reaction. The current SPC can be accessed via www.emc.medicines.org.uk.

Adverse events (AE) should be graded using the NCI Common Terminology Criteria for Adverse Events (CTCAE) Version 3.0. The toxicity grades should be recorded on the toxicity part of the CRF.

Please note that a SAE form is not considered as complete unless the following details are provided:

- Full participant trial number
- An adverse event/reaction
- A completed assessment of the seriousness, causality and expectedness as performed by the PI or another appropriately qualified clinician registered on the delegation log

If any of these details are missing, you will be contacted and the information must be provided as soon as it becomes available.

11.1 Centre responsibilities

All SAEs and reactions must be reported by the Principal Investigator at the participating centre to the WCTU unless the SAE/SAR is specified as not requiring immediate reporting (see below). All other AEs should be reported on either the treatment or follow-up CRFs as usual. The PI, or another appropriately qualified clinician registered on the delegation log, should assess the SAE to determine whether it is a reaction to protocol treatment (graded as definite, probable, possible or not related) and whether it is unexpected or expected in view of the SPCs or IB. A completed SAE form for all events requiring immediate reporting should be faxed to the WCTU within 24 hours of knowledge of the event.

SAE Fax Number: 029 2064 4488

In addition to the standard SAE definition for the purposes of this protocol certain cetuximab hypersensitivity reactions (section 8.4.2) should be reported as SAEs.

The following SAEs **do not** require immediate reporting:

- Disease progression or death as a result of disease progression
- Hospitalisation as a result of disease progression (for example, procedures involving the insertion of a PEG or stent as these are procedures for routine management)
- Hospitalisation as a result of expected adverse events related to anti-cancer therapy (for example neutropenic sepsis)

These should be completed on the relevant CRF and forwarded to WCTU in the usual timeframes for CRF pages.

11.2 Reporting pregnancies whilst participating in SCOPE 1

Patients who are pregnant are excluded from participating in the SCOPE 1 clinical trial. The reason for this being risks involved if a foetus is exposed to chemotherapy and radiotherapy. However, it is important to consider the potential for foetal exposure to these agents (resulting from maternal or paternal exposure) and have suitable procedures in place if this should arise. Therefore, in the event of a pregnancy in a trial participant, or the partner of a trial participant, the WCTU must be contacted within 24 hours to request a Pregnancy Report Form. The Pregnancy Report Form should be completed and returned to the WCTU to capture all the relevant information required for the expedited reporting of these events. The outcome of a pregnancy should be followed up carefully and any abnormal outcome of the mother or the foetus should be reported. This also applies to pregnancies following the administration of the investigational product to the father prior to sexual intercourse.

11.3 WCTU responsibilities

Following the initial report of an SAE, further follow up information may be requested by the WCTU. The patient will be identified by trial number, date of birth and initials only. The patient's name should not be used on any correspondence.

Velindre Hospital NHS Trust is undertaking the duties of trial sponsor and has delegated to the WCTU the responsibility for the reporting of SUSARs and other SARs to the regulatory authorities (MHRA and the relevant Ethics Committees) as follows:

- SUSARs which are fatal or life-threatening must be reported not later than 7 days after the sponsor is first aware of the reaction. Any additional relevant information must be reported within a further 8 days.

- SUSARs that are not fatal or life-threatening must be reported within 15 days of the sponsor first becoming aware of the reaction.

- A list of all SARs (expected and unexpected) will be reported annually to the MHRA and MREC and a copy sent to all the PIs.
- The WCTU will report a list of all SARs (expected and unexpected) and any other safety recommendations to all PIs every six months throughout the course of the trial. This frequency may be reviewed and amended as necessary.

Once an SAE is received at the WCTU, it will be evaluated by staff at the WCTU and the Chief Investigator (or their delegate) for seriousness, expectedness and causality. Investigator reports of suspected SARs will be reviewed immediately and those that are SUSARs identified and reported to the MHRA, MREC and Merck KGaA.

The causality and expectedness assessment given by the PI cannot be overruled by the CI (or their delegate) and in the case of disagreement, both opinions will be provided with the report. SAEs will be handled at the highest level of event categorisation. This ensures that all SAEs which are assessed as SARs or SUSARs (by either the CI or PI at

any stage) will be reported correctly and included into annual and investigator safety reports.

Flowchart for SAEs

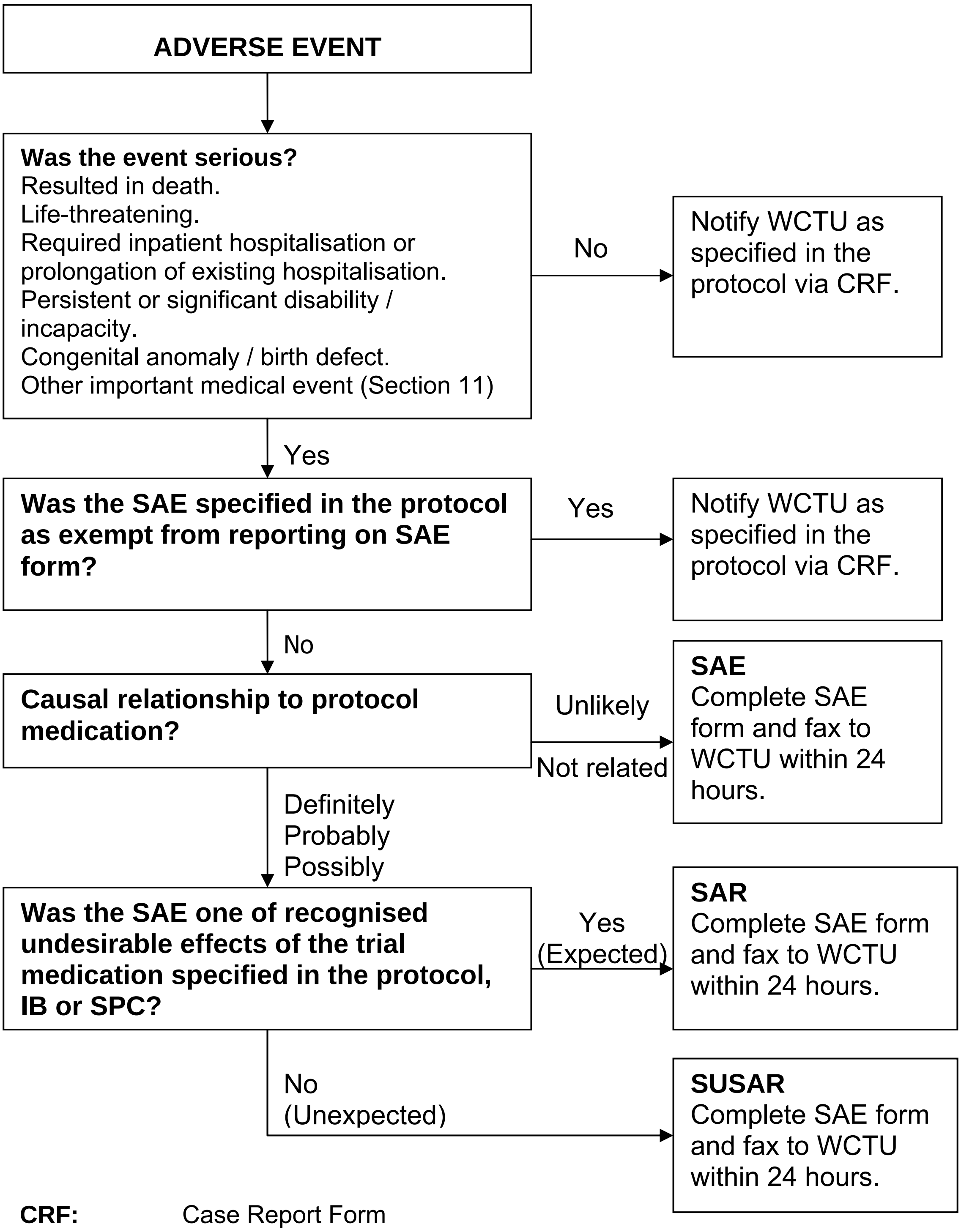

**CRF:**	Case Report Form
**IB:**	Investigator Brochure
**SAE:**	Serious Adverse Event
**SAR:**	Serious Adverse Reaction
**SPC:**	Summary of Product Characteristics
**SUSAR:**	Suspected Unexpected Serious Adverse Reaction

12. STATISTICAL CONSIDERATIONS

12.1 Method of randomisation

Patients will be randomised centrally using the method of minimisation which includes a random element. Patients will be stratified for a number of clinically important stratification factors. The randomisation ratio for control:research arm will be 1:1.

The trial will be conducted in two stages.

12.2 Outcome measures

Stage 1:
The primary outcome measure is treatment failure rate in the research arm. Treatment failure rate will be assessed at 24 weeks (12 weeks after completion of CRT) and is defined as pathologically confirmed residual disease by endoscopic assessment and biopsy and on CT scan of thorax and abdomen.

The secondary outcome measures are: toxicity and feasibility.

- **Toxicity:** will be scored using the NCI CTCAE v3.0 and RTOG acute radiation morbidity scoring criteria (Appendix XII) at baseline, during treatment and at pre-specified time points on follow-up. Serious adverse events will be monitored "real-time" by the Chief Investigator and TMG.
- **Feasibility:** will be measured through the number of protocol dose modifications and delays.

Stage 2:
The primary outcome measure is overall survival (OS). Overall survival is calculated from the date of randomisation to death from any cause. Those patients still alive will be censored at the date last seen.

The secondary outcome measures are: Toxicity, quality of life, quality assurance and health economics.

- **Toxicity:** will be scored using the NCI CTCAE v3.0 and RTOG acute radiation morbidity scoring criteria at baseline, during treatment and at pre-specified time points on follow-up. Serious adverse events will be monitored "real-time" by the Chief Investigator and TMG.
- **Quality of Life (QoL):** will be documented through the use of EORTC-C30 plus oesophageal specific modules (OES18) and dermatology life quality index (DLQI) at baseline, during treatment and at pre-specified time points on follow-up.
- **Quality assurance (QA):** A detailed QA program will be in place to ensure adherence to the protocol (please see separate radiotherapy protocol). Major and minor deviations will be collected.
- **Health Economics (HE):** HE analysis will be performed using appropriate data collected on the CRFs, from relevant cost and resource utilisation data collected

from finance and clinical staff, from administration of EQ-5D at baseline, during treatment and at pre-specified time points on follow-up.

NB: Although activity, safety and feasibility will not be formally assessed in the control arm in Stage 1, the randomisation of patients will continue using the same process and on a 1:1 allocation and therefore those patients in Stage 1 will be included in the analysis of Stage 2.

12.3 Sample size calculation

Stage 1:
The treatment failure-free rate in patients treated with CRT is approximately 60% at 24 weeks [23-30]. With the addition of cetuximab it is felt that a treatment failure-free rate of less than 60% would not be sufficiently large enough to warrant further investigation in a Phase III setting, but that a rate of 75% or higher would warrant further investigation. Using a Fleming's single stage design, p_1=0.60 and p_2=0.75, setting α=0.05 and 90% power 83 patients would be required. To allow for 10% not being assessable and with a 1:1 allocation 90 patients will be recruited into each arm, a total of 180 patients. The IDMC will review the treatment failure-free rate, along with toxicity and feasibility, before they endorse continuation into Stage 2.

Stage 2
Calculations were performed using the ART package[41] in Stata 9 assuming a 3 year recruitment period followed by 1 year of follow up (based on a 2-sided log rank test). The 2 year overall survival rate in patients treated with CRT is approximately 35%. In order to detect an improvement in 2 year overall survival from 35% to 47.5% (Hazard Ratio [HR] =0.71) with 80% power at a 5% significance level a total of 420 patients (269 events) are required. A total of 210 patients will be recruited to each arm.

12.4 Interim monitoring and analyses

The first planned analysis of both trial arms will occur after a total of 60 patients have been recruited (30 patients in each arm). The research arm will be analysed to exclude any unexpected early toxicities, notably an excess of mucositis.

Interim analysis of the accumulating data will then be performed at regular intervals (annually) for review by the IDMC. These analyses will be performed by the WCTU. The IDMC will review the outcome Stage 1 is complete to endorse continuation into Stage 2.

The IDMC will review the results and, with consideration of any results from any relevant external trials, make a recommendation to the Trial Steering Committee (TSC) on whether the trial should continue recruitment or close to recruitment. While the trial is recruiting, only the appropriate staff at the WCTU and the IDMC will see the result of the interim analyses.

12.5 Statistical analyses

All analyses will be on an intention-to-treat basis, i.e. all patients randomised will be included, and all patients will be analysed according to their allocated group whatever treatment they received.

A detailed statistical analysis plan will be prepared and finalised before analysis of the data.
The main analysis will compare overall survival between the two groups using a logrank test.

Kaplan Meier curves and logrank tests will also be used to compare the two groups on other tome to event outcomes of toxicities. The secondary outcomes of proportions of patients with SAEs will be compared using a chi-squared test.

12.6 Subgroup analyses

No formal subgroup analyses are planned. However, if any treatment difference is found we will look to see whether it is consistent across patient subgroups (defined by all pre-treatment factors collected) although this analysis will be exploratory in nature.

13. INFORMED CONSENT, ETHICAL & REGULATORY CONSIDERATIONS

13.1 Ethical and other issues

The protocol will have the favourable opinion of a Multi-centre Research Ethics Committee (MREC) but a REC must also approve each centre, through the SSI process, before patients are recruited at that centre. All patients must be informed of the aims of the study, the known possible adverse events, the procedures and possible hazards to which they may be exposed. They will be informed of the strict confidentiality of their data, but that their medical records may be reviewed for trial purposes by authorised individuals other than their treating physician.

The patient's consent to participate in the trial should be obtained after a full explanation has been given of the treatment options, including the conventional and generally accepted methods of treatment. The patient's consent will also be sought to notify their GP of their involvement in the trial. Patients should be given sufficient time after being given the trial Patient Information Sheet, to consider and discuss participation in the trial with friends and family. A contact number should be given to the patient should they wish to discuss any aspect of the trial. Following this, the randomising investigator should determine that the patient is fully informed of the trial and their participation is in accordance with UK regulations. Patients should always be asked to sign and date a consent form. One copy should be given to the patient but the original copy should be kept in the study site file and a further copy should be kept with patient's hospital notes.

The right of the patient to refuse to participate in the trial without giving reasons must be respected. After the patient has entered the trial, the investigator must remain free to give alternative treatment to that specified in the protocol, at any stage, if he/she feels it to be in the best interest of the patient. However, the reason for doing so should be recorded and the patient will remain within the trial for the purpose of follow-up and data analysis according to the treatment option to which he/she has been allocated. Similarly, the patient must remain free to withdraw at any time from the protocol treatment without giving reasons and without prejudicing his/her further treatment.

This is a randomised controlled trial therefore neither the patients nor their physicians will be able to choose the patient's treatment. Treatment will be allocated randomly using a computer-based algorithm. This is to ensure that the groups of patients receiving each of the different treatments are similar.

13.2 Regulatory status

The trial is being performed under a Clinical Trial Authorisation (CTA) from the MHRA. This is conditional on all requirements for reporting of adverse events being met, in particular Suspected Unexpected Serious Adverse Reactions (SUSARs). These must be notified by centres to the WCTU who will forward them to the Medicines and Healthcare products Regulatory Agency (MHRA).

13.3 Sponsorship and indemnity

The sponsor of the trial is Velindre NHS Trust as employer of the staff at the Wales Cancer Trials Unit and the Chief Investigator.

Velindre NHS Trust has sponsorship responsibilities as follows:

I. Obtaining trial authorisation and appropriate ethics committee opinion – with the following responsibilities delegated to WCTU:

- Request a Clinical Trial Authorisation (CTA) and subsequent amendments.
- Selection of Investigators and ensuring each centre has full trial documentation.
- Ensuring an appropriate ethics committee opinion has been sought and any amendments approved.
- Giving notice to the MHRA when the trial has ended.

II. Pharmacovigilance – with the following responsibilities delegated to WCTU:

- Keeping records of all adverse events reported by Investigators.
- Ensuring recording and prompt reporting of serious adverse reactions to the CI.
- Reporting to MHRA and main REC any SUSARs within specified timeframes.
- Ensuring Investigators informed of SUSARs.
- Providing annual listing of all SARs to MHRA, Investigators, IDMC and MREC.

III. Ensuring that the research is conducted in accordance with the principles of Good Clinical Practice (GCP) with the following responsibilities delegated to WCTU:

- Having quality control systems in place to ensure that the study is conducted according to GCP at all participating centres.

The following responsibilities are delegated to the individual participating centres:

- Have in place arrangements to adhere to GCP
- Keep a copy of all essential documents (as defined in ICH GCP) and ensure appropriate archiving and destruction once the study has ended
- Take appropriate urgent safety measures.

Non-negligent harm: This trial is an academic, Investigator-led and designed trial, coordinated by the WCTU. The Chief Investigator, local Investigators and coordinating centre do not hold insurance against claims for compensation for injury caused by participation in a clinical trial and they cannot offer any indemnity. The Association of the British Pharmaceutical Industry (ABPI) guidelines will not apply.

Negligent harm: Where studies are carried out in a hospital, the hospital continues to have a duty of care to a patient being treated within the hospital, whether or not the patient is participating in this trial. Velindre NHS Trust does not accept liability for any breach in the other hospital's duty of care, or any negligence on the part of employees of hospitals. This applies whether the hospital is an NHS Trust or not.

13.4 Data protection

The WCTU will act to preserve patient confidentiality and will not disclose or reproduce any information by which patients could be identified (except where patients are registered with the Office for National Statistics or traced via the NHS Central Register, which requires separate consent). Data will be stored in a secure manner and our trials are registered in accordance with the Data Protection Act 1998 and with the Data Protection Officer at Velindre NHS Trust Cardiff.

13.5 Publication policy

Data from all centres will be analysed together and published as soon as possible. Individual participants may not publish data concerning their patients that are directly relevant to questions posed by the trial until the Trial Management Group (TMG) has published its report. The TMG will form the basis of the Writing Committee and advise on the nature of publications.

All publications shall include a list of participants, and if there are named authors, these should include the Chief Investigator, Co-Investigators, Trial Manager, and statistician(s) involved in the trial. If there are no named authors then a writing committee will be identified.

13.6 Finance

SCOPE 1 is funded by the Clinical Trials Advisory and Awards Committee (CTAAC), Funder Number CRUK/07/03, (on behalf of Cancer Research UK, Medical Research Council, and other charities). The trial is in the National Cancer Research Network (NCRN) portfolio and therefore local NCRN support should be available at each participating centre to support entry of patients into this trial.

Cetuximab is manufactured by Merck KGaA who has agreed to supply free cetuximab for all research arm patients for the duration of the protocol treatment.

14. QUALITY OF LIFE

Health related quality of life (HRQL) is a secondary outcome for the Phase III part of this trial. The purpose for measuring HRQL in this trial is to assess patients' perceptions of the benefits and harms of treatment that can be subsequently used to fully inform clinical decision-making. In this study it is hypothesised that in the experimental arm there will be fewer patients with recurrent disease and this will lead to better survival. This improved clinical outcome may reflect in HRQL and better scores for physical, role and social function as well as fewer symptoms of fatigue, anorexia and eating difficulties during and following treatment. In patients undergoing the experimental arm it is also hypothesised that the addition of cetuximab to chemoradiotherapy may add additional HRQL problems during treatment (e.g. skin toxicity).

HRQL aims

- To determine whether cetuximab in addition to chemoradiation improves generic and disease specific aspects of HRQL following treatment than chemoradiation alone. Specific HRQL domains that are expected to be better in the experimental group are: physical and role function, fatigue, dysphagia and eating restrictions
- To determine whether cetuximab in addition to chemoradiation is associated with poorer HRQL during treatment. Specific HRQL domains that are expected to be worse in the experimental arm include: dyspnoea, skin rashes and diarrhoea.

HRQL instruments

Generic domains of HRQL will be assessed with the EORTC core Quality of Life Questionnaire, the EORTC QLQ-C30[42]. This instrument has been well validated in many international clinical trials in oncology including oesophageal adenocarcinoma and squamous cell cancer. The full 30-item measure is composed of multi-item and single scales. These include five functional scales (physical, role, emotional, social and cognitive), three symptom scales (fatigue, nausea and vomiting, and pain), a global health status scale, and six single items (dyspnoea, insomnia, appetite loss, constipation, diarrhoea and financial difficulties). All scales and single items meet the standards for reliability and validity. Disease specific and chemoradiotherapy associated symptoms and side effects will be assessed with the oesophageal cancer specific module, the EORTC QLQ-OES18[43]. This has been validated and tested in patients receiving definitive chemoradiotherapy. It demonstrates good psychometric properties including sensitivity to change over time. The module includes scales assessing dysphagia, eating restrictions, reflux, dry mouth and problems with saliva and deglutition. The Dermatology Life Quality Index (DLQI) will also be administered[44]. This is a well validated, easy to use index which assesses the impact of dermatological conditions on patients' quality of life[45]. It has been included to accurately assess the impact of the acneiform eruption commonly seen with cetuximab. In addition to using the generic and site-specific questionnaires it is proposed to design an additional scale to assess specific symptoms and side effects of cetuximab. At present there are no validated instruments designed for this purpose. During the pilot study, this scale will be developed according to EORTC guidelines for questionnaire development and supervised by Professor Blazeby in Bristol. It is expected that the final scale will be ready to use within 6 months of the start of the trial.

Study design

This is a multi-centre study and the HRQL evaluation will involve all patients in all participating centres. Patients will be informed in the Patient Information Sheet that they will have their HRQL assessed regularly while involved in the trial.

Mode and timing of data collection

Baseline questionnaires will be given to the patient in clinic after written consent has been obtained and prior to randomisation. Follow-up questionnaires will be completed by the patient during attendance at hospital for clinical visits. Patients will be asked to fill out the questionnaires themselves as completely and accurately as possible. The average time to complete the entire questionnaire is 10-15 minutes. The trials unit will record whether the HRQL forms have been filled in within the specified time window. Reasons for questionnaire non-completion will be recorded. All patients who participate in the SCOPE 1 study will complete questionnaires at all time points.

Timing of HRQL assessment

- Baseline questionnaire.
- During and at end of treatment (Week 7 and week 13).
- Post treatment (24 weeks, time of endoscopy and scan).
- Subsequent follow up will occur at 12, 18, 24 months and annually to 60 months.

Sample size

This is based upon the calculations for the primary outcome of the trial. A separate sample size calculation for the HRQL study has not been performed.

Statistical analysis plan

Data will be scored according to the algorithms described in the EORTC QLQ-C30 scoring manual [46]. All scales and single items are scored on categorical scales and linearly transformed to 0-100 scales where: (a) a high score for a symptom scale or item represents a high level of symptoms or problems, and (b) a high score for a functional scale represents a high or health level of functioning and a high score for the global health status/HRQL represents high QL. Groups of patients will be compared at agreed time points and overall for differences in these parameters. The treatment groups will be compared at the individual time points with appropriate adjustments being made for multiple comparisons. Because of the longitudinal nature of the data, an analysis that takes into account the repeated measures is also needed. A generalised linear modelling approach will be adopted. This will allow the appropriate error distribution to be used and will enable the analysis to take account of important prognostic factors.

15. HEALTH ECONOMICS

Cost-utility analysis of cetuximab in addition to chemoradiation in oesophageal cancer

Aim: The aim of this sub study is to assess the cost-utility of cetuximab in addition to chemoradiation in the treatment of patients with oesophageal cancer.

Perspective: The perspective to be employed is that of the UK National Health Service, but with consideration also given to patient and family related costs.

Design: The cost-utility analysis will involve a comparison of the additional costs associated with the use of cetuximab in the treatment regimen ± any changes in resources utilised elsewhere.

Costs and resource utilization
Costs of cetuximab will be based on discussions with clinicians and finance staff*, while other healthcare resources utilised by patients during the study period will be collected via the healthcare resource utilisation log administered during treatment and at follow-up. At each of the 3-4 weekly visits, patients will be asked to indicate whether they have had any contacts with their GP, practice nurse, community nurse or attended hospital either as an outpatient or in-patient. They will be asked whether the contact was connected to their condition or for any other purpose. The involvement of others in providing transport and/or support will also be logged. In addition, they will be asked to indicate the medication they have been taking during the 3-4 week period. Consultations with healthcare professionals will be costed using published sources of unit costs and healthcare resources utilised will be added to the respective treatment costs in each arm.

Quality adjusted life years (QALYs)
QALYs will be derived from survival data generated during the second stage of the trial and from the EQ-5D# (Appendix VIII) scores collected at baseline, during treatment and at follow-up. The patient diary will also be used to collect additional data.

Cost per QALY
Differences between the two groups in terms of resources utilised and QALYs will be determined and a cost per QALY estimate produced.

Sensitivity analysis
A probabilistic sensitivity analysis will be undertaken and a cost-effectiveness acceptability curve produced. In addition a series of one-way sensitivity analyses will be undertaken to assess the robustness of the estimate to changes in costs, resources utilised, utility scores and QALYs.

*The cost will be assessed despite being made available for the trial by Merck KGaA
#EQ-5D is generally accepted as appropriate for determination of QALYs

16. T-SCOPE (TRANSLATIONAL LABORATORY RESEARCH)

The SCOPE 1 trial is an excellent opportunity to prospectively collect EUS biopsy samples for a range of translational research. Translational studies would look at a number of key areas;

- Determine to what extent EGFR status could predict the response to cetuximab.
- Establish a correlation between EGFR inhibition and DNA repair.
- Determine the effect of cetuximab on overall DNA repair.

Translational studies would also be able to look at other aspects of oesophageal cancer and the effect of CRT, and include histological and biochemical investigations. These could include:

- Identify molecular predictors of outcome independent of tumour staging for patients with oesophageal cancer undergoing CRT.
- Determine DNA damage caused by CRT and link this to the cell cycle and related protein expression / synthesis and pathways.
- Describe the relationship between tumour cell density and composition of the interstitium to determine the interstitial diffusion coefficient of therapeutic agents
- Provide more detailed histological characterization of this type of tumour.

Blood samples will allow both heterozygosity studies to be carried out and biochemical markers to be investigated.

Details of these translational studies will be contained within separate proposals to reflect the expertise of the group carrying out this research. The WCTU will co-ordinate these activities which will include transport, storage and distribution of the samples. The WCTU will ensure data is confidential and specific information is released only at times agreed by the IDMC.

The main protocol covers pre and post treatment paraffin embedded biopsy samples. A matched blood sample will also be collected and stored. Paraffin embedded biopsy samples are also requested if biopsy is taken as a result of clinical decision to investigate disease progression during follow up. This does not exclude the possibility of fresh frozen sample, or sample collected into fixation solution being added. This would constitute a separate proposal and ethics carried out for a sub-set of patients for specific measures providing the expected number of samples will give sufficient power to provide statistical significance and are supported by sufficient funding.

A separate section is included in the consent form for the storage of paraffin blocks and the collection and storage of blood samples for translational research.

17. QUALITY ASSURANCE OF RADIOTHERAPY

For the SCOPE 1 trial there is a Quality Assurance of Radiotherapy sub-study. This will consist of the following:

17.1 Educational component

An educational CD ROM is being distributed to all investigators. This CD ROM includes three example cases, each of which consist of a clinical summary, CT slice data, outlines and Radiotherapy plan information. GUINESS software is provided for viewing the CT, outline and planning data.

A radiotherapy treatment planning and delivery protocol has been written and is supplied in hard copy and electronically on the CD ROM.

17.2 Test Case

All clinical and radiation oncologists participating in the trial are required to outline a test case. All planning departments are required to plan this test case. This would usually be carried out on the outline completed by the Principal Investigator. The CT data set is included on the educational CD ROM.

All centres and Investigators must have a 'Pass' for the planned test case before entering patients into the trial.

17.3 Questionnaires

Centres are required to complete the following questionnaires:

National QA for Clinical Trials baseline questionnaire if this has not already been completed

SCOPE 1 specific questionnaire

17.4 Equipment validation

A visit by the QA team to validate dosimetry for this body region if not already carried out for the INCH or CONVERT trial.

17.5 Patient cases

On trial, centres are required to provide a full dataset of images, structures, plan and doses for each patient. The first clinical case from each investigator, a 10% sample and those with noted major deviations from the protocol will be analysed real time. However centres are to continue with planned treatment and not to wait for a response from the QA team.

The data from the QA RT programme will be analysed separately from the main trial. The participants will be notified of any major deviations.

18. TRIAL COMMITTEES AND TRIAL MANAGEMENT ARRANGEMENTS

The Trial Management Group (TMG) co-ordinates and manages the trial's day-to-day activities. The TMG is comprised of the Chief Investigator, other Co-Investigators and members of the WCTU.

The data will be reviewed (periodically) by an Independent Data Monitoring Committee (IDMC) as described in the trial specific IDMC charter, consisting of at least two Clinicians who are not responsible for patients entered into the trial and an independent Statistician. The IDMC will be asked to recommend whether the accumulated data from the trial, together with results from other relevant trials, justifies continuing recruitment of further patients. A decision to discontinue recruitment, in all patients or in selected subgroups will be made only if the result is likely to convince a broad range of clinicians including trial Investigators and the general clinical community. If a decision is made to continue, the IDMC will advise on the frequency of future reviews of the data on the basis of accrual and event rates. The IDMC will make confidential recommendations to the Trial Steering Committee (TSC).

The role of the TSC is to act on behalf of the sponsor and funder, to provide overall supervision for the trial, to ensure that it is conducted in accordance with GCP, and to provide advice through its independent chairman. This independent committee will review the recommendations from the IDMC and will decide on continuing or stopping the trial, or modifying the protocol.

19. REFERENCES

1. Parkin DM, Bray F, Ferlay J, Pisani P: **Global Cancer Statistics, 2002**. *CA Cancer J Clin* 2005, **55:**74-108.

2. Cancer Research UK. UK Oesophageal Cancer Statistics. Cancer Research UK. 2006. www.cancerresearchuk.org/statistics. Accessed 3-8-2006.

3. Allum WH, Griffin SM, Watson A, Colin-Jones D: **Guidelines for the management of oesophageal and gastric cancer**. *Gut* 2002, Suppl V, **50:**1v-23.

4. Medical Research Council Oesophageal Cancer Working Party: **Surgical resection with or without preoperative chemotherapy in oesophageal cancer: a randomised controlled trial**. *The Lancet* 2002, **359 (9319):** 1727-1733.

5. Coia LR, Minsky BD, Berkey BA, John MJ, Haller D, Landry J, Pisansky TM, Willett CG, Hoffman JP, Owen JB, Hanks GE: **Outcome of patients receiving radiation for cancer of the esophagus: Results of the 1992-1994 Patterns of Care Study**. *Journal of Clinical Oncology* 2000, **18 (3):**455-62.

6. Fok M, Law SY, Wong J: **Operable esophageal carcinoma: current results from Hong Kong**. *World Journal of Surgery* 1994, **18 (3):**355-60.

7. Muller JM, Erasmi H, Stelzner M, Zieren U, Pichlmaier H: **Surgical therapy of oesophageal carcinoma**. *British Journal of Surgery* 1990, **77(8):**845-57.

8. Pye JK, Crumplin MK, Charles J, Kerwat R, Foster ME, Biffin A, Hospital Clinicians in Wales: **One -year survey of carcinoma of the oesophagus and stomach in Wales**. *British Journal of Surgery* 2001, **88 (2):**278-85.

9. Cooper JS, Guo MD, Herskovic A, Macdonald JS, Martenson JA Jr, Al-Sarraf M, Byhardt R, Russell AH, Beitler JJ, Spencer S, Asbell SO, Graham MV, Leichman LL: **Chemoradiotherapy of locally advanced esophageal cancer: long-term follow-up of a prospectice randomized trial (RTOG 85-01)**. *Journal of the American Medical Association* 1999, **281(17):**1623-7.

10. Wong RK, Malthaner RA, Zuraw L, Rumble BR, Cancer Care Ontario Practice Guidelines Initiative Gastrointestinal Cancer Disease Site Group: **Combined modality radiotherapy and chemotherapy in nonsurgical management of localized carcinoma of the esophagus: a practice guideline**. *International Journal of Radiation Oncology, Biology, Physics* 2003, **55 (4):**930-42.

11. Bedenne L, Michel P, Bouche O, Triboulet J-P, Conroy T, Pezet D, Roullet B, Seitz J-F, Lacourt J, Milan C: **Randomized phase III trial in locally advanced esophageal cancer: radiochemotherapy followed by surgery versus radiochemotherapy alone (FFCD 9102)**. *Proceedings of American Society of Clinical Oncology* 2002, Journal of Oncology **21:**abstr 519.

12. Crosby TD, Brewster AE, Borley A, Perschky L, Kehagioglou P, Court J, Maughan TS: **Definitive chemoradiation in patients with inoperable oesophageal carcinoma**. *British Journal of Cancer* 2004, **90(1):**70-5.

13. Denham JW, Steigler A, Kilmurray J, Wratten C, Burmeister B, Lamb DS, Joseph D, Delaney G, Christie D, Jamieson G, Smithers BM, Ackland S, Walpole E: **Relapse patterns after chemo-radiation for carcinoma of the oesophagus**. *Clinical Oncology (Royal College of Radiologists (Great Britain))* 2003, **15(3):**98-108.

14. Minsky BD, Pajak TF, Ginsberg RJ, Pisansky TM, Martenson J, Komaki R, Okawara G, Rosenthal SA, Kelsen DP: **INT 0123 (Radiation Therapy Oncology Groups 94-05) phase III trials of combined-modality therapy for esophageal cancer: high-dose versus standard-dose radiation therapy**. *Journal of Clinical Oncology* 2002, **20(5):**1167-74.

15. Herskovic A, Martz K, al-Sarraf M, Leichman L, Brindle J, Vaitkevicius V, Cooper J, Byhardt R, Davis L, Emami B. **Combined chemotherapy and radiotherapy compared with radiotherapy alone in patients with cancer of the esophagus**. New England Journal of Medicine 2006, 326(24), 1593-8.

16. Stahl M, Wilke H, Walz MK. **A Randomized phase III trial in locally advanced squamous cell carcinoma (SCC) of the esophagus: Chemoradiation with and without surgery**. *Proceeding of the Annual Meeting of the American Society of Clinical Oncology (ASCO), Journal of Oncology* 2003, **22,** abstr 1001.

17. Gill V, Melcher A, Crellen A: **Chemoradiotherapy for Oesophageal Cancer: The Leeds Cancer Centre Experience**. *Clinical Oncology (Royal College of Radiologists (Great Britain))* 2003, **15**.

18. Tsutsumi K, Yasuda M, Nishioka T: **X-ray irradiation altered chemosensitivity of a p53-null non-small cell lung cancer cell line.** *Cell Structure and Function* 2006, **31(2):**47-52.

19. Byfield JE, Calabro-Jones P, Klisak I, Kulhanian F: **Pharmacologic requirements for obtaining sensitization of human tumor cells in vitro to combined 5-Fluorouracil or ftorafur and X rays**. *International Journal of Radiation Oncology, Biology, Physics* 1982, **8(11):**1923-33.

20. Cunningham D, Rao S, Starling N, Iveson T, Nicolson M, Coxon F, Middleton G, Daniel F, Oates J, Norman AR, NCRI Upper GI Study Group: **Randomised multicentre phase III study comparing capecitabine with fluorouracil and oxaliplatin with cisplatin in patients with advanced oesophagogastric (OG) cancer: The REAL 2 trial**. *Proceeding of the Annual Meeting of the American Society of Clinical Oncology (ASCO), Journal of Clinical Oncology* 2006, **24: abstr**LBA4017.

21. Das P, Wolff RA, Abbruzzese JL, Krishnan S, Varadhachary GR, Xiong HQ, Evans DB, Vauthey JN, Baschnagel A, Crane CH: **Concurrent capecitabine and upper abdominal radiation therapy is well-tolerated**. *Proceeding of the American Society of Clinical Oncology (ASCO)GI cancers symposium. Journal of Clinical Oncology* 2006, abstr106.

22. Morgan C, Brewster AE, Maughan TS, Harvey-Jones A, Crosby TDL: **Patterns of failure after definitive chemo-radiation for inoperable carcinoma of the oesophagus**. *Clinical Oncology* 2004, **16:**S15.2.

23. Steel GG: *Basic Clinical Radiobiology*, edn 3. Edited by G Gordon Steel. 2002.

24. Baumann M, Krause M: **Targeting the epidermal growth factor receptor in radiotherapy: radiological mechanism, preclinical and clinical results**. *Radiotherapy and Oncology: Journal of the European Society for Therapeutic Radiology and Oncology* 2004, **72 (3):**257-66.

25. Schmidt-Ullrich RK, Mikkelsen RB, Dent P, Todd DG, Valerie K, Kavanagh BD, Contessa JN, Rorrer WK, Chen PB: **Radiation-induced proliferation of the human A431 squamous carcinoma cells is dependent on EGFR tyrosine phosphorylation**. *Oncogene* 1997, **15(10):**1191-7.

26. Bonner JA, Harari PM, Giralt JL, Azarnia N, Shin DM, Cohen RB, Joes CU, Sur R, Raben D, Jassem J, Ove R, Kies MS, Baselga J, Youssoufian H, Amella N, Rowinsky EK, Ang KK. **Radiotherapy plus cetuximab for squamous-cell carcinoma of the head and neck.** *New England Journal of Medicine* 2006, **354(6):**567-78.

27. Cunningham D, Humblet Y, Siena S, Khayat D, Bleiberg H, Santoro A, Bets D, Mueser M, Harstrick A, Verslype C, Chau I, Van Cutsem E: **Cetuximab monotherapy and Cetuximab plus irinotecan in irinotecan-refractory metastic colorectal cancer**. *The New England Journal of Medicine* 2004, **351(4):**337-45.

28. Rischin D, Burmeister B, Mitchell P, Boyer M, Macmanus M, Walpole E, Feigen M, Tin MM, Ball D: **Phase I trial of gefitinib (ZD1839) in combination with concurrent carboplatin, paclitaxel and radiation therapy in patients with stage III non-small cell lung cancer**. *Journal of Clinical Oncology* 2004, **22(14S):** abstrA7077.

29. Werner-Wasik M, Swann S, Curran W, Robert F, Komaki R, Lee CP, Jafar S, Share R, Choy H, Blumenschein G: **A Phase II Study of Cetuximab (C225) In Combination with Chemoradiation (CRT) in Patients (PTS) with Stage IIIA/B Non-Small Cell Lung Cancer (NSCLC): An interim overall Toxicity Report of the RTOG 0324 Trial**. *Proceeding of the Annual Meeting of the American Society of Clinical Oncology (ASCO)* 2005, Journal of Clinical Oncology **23 (16S): abstr7135**.

30. Suntharalingam M, Dipetrillo T, Akerman P, Wanebo H, Daly B, Doyle LA, Krasna MJ, Kennedy T, Safran H: **Cetuximab, paclitaxel, carboplatin and radiation for esophageal and gastric cancer**. *Proceeding of the Annual Meeting of the American Society of Clinical Oncology (ASCO)* 2006, *Journal of Clinical Oncology*, **24(18S):**Abstr4029.

31. Dobelbower MC, Russo SM, Raisch KP, Seay LL, Clemons LK, Suter S, Posey J, Bonner JA: **Epidermal growth factor receptor tyrosine kinase inhibitor, erlotinib, and concurrent 5-fluorouracil, cisplatin and radiotherapy for patients with esophageal cancer: a phase I study**. *Anticancer Drugs* 2006, **17(1):**95-102.

32. Lim JT, Truong PT, Berthelet E, Pai H, Joe H, Wai E, Larsson S, Kader HA, Weinerman B, Wilson K, Olivotto IA: **Endoscopic response predicts for survivial and organ preservation after primary chemoradiotherapy for esophageal**

cancer. *International Journal of Radiation Oncology, Biology, Physics* 2003, **57(5):**1328-35.

33. Hui R, Bull CA, Gebski V, O'Rouke I: **Radiotherapy and concurrent chemotherapy for oesophageal carcinoma**. *Australas Radiology* 1994, **38(4):**315-9.

34. Gill PG, Jamieson GG, Denham J, Devitt PG, Ahmed A, Yeoh E, Jones AM: **Treatment of adenocarcinoma of the cardia with synchronous chemotherapy and radiotherapy**. *British Journal of Surgery* 1990, **77:**1020-1023.

35. Bates BA, Detterbeck FC, Bernard SA, Qaqish BF, Tapper JE: **Concurrent radiation therapy and chemotherapy followed by esophagectomy for localized esophageal carcinoma**. *Journal of Clinical Oncology* 1996, **14(1):**156-63.

36. John MJ, Flam MS, Mowry PA, Podolsky WJ, Xavier AM, Wittlinger PS, Padmanabhan A: **Radiotherapy alone and chemoradiation for nonmetastatic esophageal carcinoma. A critical review of chemoradiation**. *Cancer* 1989, **63(12):**2397-403.

37. Poplin EA, Khanuja PS, Kraut MJ, Herskovic AM, Lattin PB, Cummings G, Gaspar LE, Kinzie JL, Steiger Z, Vaitkevicius VK: **Chemoradiotherapy of esophageal carcinoma**. *Cancer* 1994, **74(4):**1217-24.

38. Seitz JF, Perrier H, Monges G, Giovannini M, Gouvernet J: **Multivariate analysis of the prognostic and predictive factors of response to concomitant radiochemotherapy in epidermoid cancers of the esophagus. Value of immunodetection of protein p53**. *Gastroenterolgie Clinique et Biologique* 1995, **19(5):**465-74.

39. Brown WA, Thomas J, Gotley D, Burmeister B, Lim KH, Martin I, Walpole ET, Thomson DB, Harvey JA, Smithers BM: **Use of oesophagogastroscopy to assess the response of oesophageal carcinoma to neoadjuvant therapy**. *British Journal of Surgery* 2004, **91(2):**199-204.

40. Downey RJ, Akhurst T, Ilson D, Ginsberg R, Bains MS, Gonen M, Koong H, Gollub M, Minsky BD, Zakowski M, Turnbull A, Larson SM, Rusch V. **Whole Body 18FDG-PET and the Response of Esophageal Cancer to Induction Therapy: Results of a Prospective Trial.** 2003 Journal of Clinical Oncology **21(3)**: 428-32.

41. Barthel FMS, Royston P, Babiker A, UK Medical Research Council: **A menu-driven facility for complex sample size calculation in randomized controlled trials with a survival or a binary outcome: update**. *The Stata Journal* 2005, **5 1):**129-9.

42. Aaronson NK, Ahmedzai S, Bergman B, Bullinger M, Cull A, Duez NJ, Filiberti A, Flechtner H, Fleishman SB, de Haes JC, Kaasa S, Klee M, Osoba D, Razavi D, Rofe PE, Schraub S, Sneeuw K, Sullivan M, Takeda F: **The European Organization for Research and Treatment of Cancer QLQ-C30: a quality-of-life instrument for use in international clinical trials in oncology**. *Journal of National Cancer Inst* 1993, **85(5):**365-376.

43. Blazeby JM, Conroy T, Hammerlid E, Fayers P, Sezer O, Koller M, Arraras J, Bottomley A, Vickery CW, Etienne PL, Alderson D, European Organisation for

Research and Treatment of Cancer Gastrointestinal and Quality of Life Groups: **Clinical and psychometric validation of an EORTC questionnaire module, the EORTC QLQ-OES18, to assess quality of life in patients with oesophageal cancer.** *European Journal of Cancer* 2003, **39(10):**1384-94.

44. Finlay AY, Khan GK: **Dermatology Life Quality Index (DLQI): a simple practical measure for routine clinical use**. *Clinical and Experimental Dermatology* 1994, **19(3):**210-6.

45. Lewis V, Finlay AY: **10 years experience with the dermatology life quality index (DLQI)**. *Journal of the Investigational Dermatological Symposium Porceedings* 2004, **9(2):**169-80.

46. Fayers P, Aaronson NK, Bjordal K, Groenvold M, Curran D, Bottomley A: **EORTC QLQ-C30 Scoring Manual**. 2001:0-86.

APPENDIX I

WHO Performance status

0	Able to carry out normal activity without restriction.
1	Restricted in physically strenuous activity but ambulatory and able to carry out light work.
2	Ambulatory and capable of self-care but unable to carry out any work: up and about more than 50% of working hours.
3	Capable only of limited self-care; confined to bed or chair more than 50% of waking hours.
4	Completely disabled; cannot carry out any self-care; totally confined to bed or chair.

APPENDIX II

Renal Function

Cockroft & Gault formula

$$\text{GFR for males} = \frac{1.23 \times [140\text{-age}] \times \text{weight (kg)}}{\text{Serum creatinine } (\mu\text{mol/l})}$$

$$\text{GFR for females} = \frac{1.05 \times [140\text{-age}] \times \text{weight (kg)}}{\text{Serum creatinine } (\mu\text{mol/l})}$$

- A patient with a Cockcroft & Gault estimate of GFR ≥ 60ml/min using the above formula is eligible for the trial.

- Patients with GFR < 60 ml/min should have confirmation by EDTA clearance, other isotopic method or 24hr creatinine clearance and will be eligible if the GFR ≥ 40.

- After start of treatment, if Cockcroft & Gault estimate falls by 25% from baseline to below 50 ml/min, the EDTA should be performed but an appropriate dose modification made according to protocol pending this result

Treatment modification in the case of any renal toxicity is in section 8.4 of the protocol.

APPENDIX III

WHO grades of hearing impairment

Grade of impairment	Corresponding audiometric ISO value	Performance	Recommendations
0 – No impairment	25 dB or better (better ear)	No or very slight hearing problems. Able to hear whispers.	
1 – Slight impairment	26-40 dB (better ear)	Able to hear and repeat words spoken in normal voice at 1 metre.	Counselling. Hearing aids may be needed.
2 – Moderate impairment	41-60 dB (better ear)	Able to hear and repeat words spoken in raised voice at 1 metre.	Hearing aids usually recommended.
3 – Severe impairment	61-80 dB (better ear)	Able to hear some words when shouted into better ear.	Hearing aids needed. If no hearing aids available, lip-reading and signing should be taught.
4 – Profound impairment including deafness	81 dB or greater (better ear)	Unable to hear and understand even a shouted voice.	Hearing aids may help understanding words. Additional rehabilitation needed. Lip-reading and sometimes signing essential.

Grades 2, 3 and 4 are classified as disabling hearing impairment.

The audiometric iso values are averages of values at 500, 1000, 2000, 4000 Hz.

APPENDIX IV

Investigations

Multi-slice CT scan (within 4 weeks of randomisation)

Patients should be staged with multi-slice CT to assess the local extent of the primary tumour, the presence of loco-regional lymphadenopathy and distant metastatic disease. A water load is used, dysphagia permitting, to maximise oesophageal and gastric distension. Intravenous contrast should be used and CT scan of the thorax and abdomen acquired at 1.25-2.5mm and reformatted at 5mm for viewing (RCR Guidelines 2006).

Endoscopic Ultrasound (within 4 weeks of randomisation)

Patients should be staged with endoscopic ultrasound (EUS) to assess the extent of visible and sub-mucosal tumour, infiltration by the primary tumour of surrounding mediastinal structures and the presence and site of site of significant lymhpadenopathy. The following will be recorded:
Location of disease and anatomical structures should be described according to the distance in cms from the incisor teeth.

OGD: Proximal and distal tumour extent (cms from the incisor teeth)
Amount of gastric involvement (measurement from oesophago – gastric junction

EUS: Reference point e.g. top of arch of aorta, tracheal carina
Proximal and distal tumour extent (including submucosal disease)
T stage of tumour
If T4, record involvement of surrounding structures (e.g. right/left parietal pleura, aorta, trachea, diaphragmatic crus etc)
Pathological appearing lymphadenopathy, including distance and station (right/left tracheal, aortopulmonary window, subcarinal, paraoesophageal with relationship to tumour, paracardial, left gastric)

Post Treatment Endoscopy (12 weeks after treatment)

The endoscopy should be performed according to local practice, ideally by the same clinician who performed the diagnostic endoscopy or staging EUS. Detailed information on the pre-treatment findings must be available including tumour extent in relation to the incisors. At least 6 biopsies should be taken from any site which is suspicious of tumour. If the mucosa is macroscopically normal then 2 biopsies from the top, middle and bottom of the known site of previous tumour according to the distance from the incisors. Paraffin blocks of samples will be collected and centrally reviewed.

Follow up investigations

Apart from a standard CT scan performed at 12 weeks after completion of treatment and reported according to RECIST criteria (Appendix XIII) there are no planned investigations. Patients with clinical suspicion of disease recurrence should undergo CT of thorax/abdomen and where possible an endoscopy. In the case of local recurrence it will be important to define the relationship of the recurrent disease to the radiotherapy field by comparing endoscopic data at recurrence with the pre-treatment studies, and/or in the

situation where recurrence is diagnosed on CT alone, this diagnostic data should be correlated with the CT planning films.

APPENDIX V

Quality of life questionnaire EORTC QLQ-C30

We are interested in some things about you and your health. Please answer all of the questions yourself by circling the number that best applies to you. There are no "right" or "wrong" answers. The information that you provide will remain strictly confidential.

Please fill in your initials: ___ ___ ___
Your birthdate (Day, Month, Year): __ __ / __ __ / __ __ __ __
Today's date (Day, Month, Year): __ __ / __ __ / __ __ __ __

		Not at All	A Little	Quite a Bit	Very Much
1.	Do you have any trouble doing strenuous activities, like carrying a heavy shopping bag or a suitcase?	1	2	3	4
2.	Do you have any trouble taking a <u>long</u> walk?	1	2	3	4
3.	Do you have any trouble taking a <u>short</u> walk outside of the house?	1	2	3	4
4.	Do you need to stay in bed or a chair during the day?	1	2	3	4
5.	Do you need help with eating, dressing, washing yourself or using the toilet?	1	2	3	4

**During the past week:**		Not at All	A Little	Quite a Bit	Very Much
6.	Were you limited in doing either your work or other daily activities?	1	2	3	4
7.	Were you limited in pursuing your hobbies or other leisure time activities?	1	2	3	4
8.	Were you short of breath?	1	2	3	4
9.	Have you had pain?	1	2	3	4
10.	Did you need to rest?	1	2	3	4
11.	Have you had trouble sleeping?	1	2	3	4
12.	Have you felt weak?	1	2	3	4
13.	Have you lacked appetite?	1	2	3	4

<u>Please go on to the next page</u>

SAMPLE

During the past week:		Not at All	A Little	Quite a Bit	Very Much
14.	Have you felt nauseated?	1	2	3	4
15.	Have you vomited?	1	2	3	4
16.	Have you been constipated?	1	2	3	4
17.	Have you had diarrhoea?	1	2	3	4
18.	Were you tired?	1	2	3	4
19.	Did pain interfere with your daily activities?	1	2	3	4
20.	Have you had difficulty in concentrating on things, like reading a newspaper or watching television?	1	2	3	4
21.	Did you feel tense?	1	2	3	4
22.	Did you worry?	1	2	3	4
23.	Did you feel irritable?	1	2	3	4
24.	Did you feel depressed?	1	2	3	4
25.	Have you had difficulty remembering things?	1	2	3	4
26.	Has your physical condition or medical treatment interfered with your family life?	1	2	3	4
27.	Has your physical condition or medical treatment interfered with your social activities?	1	2	3	4
28.	Has your physical condition or medical treatment caused you financial difficulties?	1	2	3	4

For the following questions please circle the number between 1 and 7 that best applies to you

29. How would you rate your overall health during the past week?

1	2	3	4	5	6	7
Very poor						Excellent

30. How would you rate your overall quality of life during the past week?

1	2	3	4	5	6	7
Very poor						Excellent

© Copyright 1995 EORTC Study Group on Quality of Life. All rights reserved. Version 3.0

SAMPLE

APPENDIX VI

Quality of life questionnaire Oesophageal Module

EORTC QLQ-OES18

Patients sometimes report that they have the following symptoms or problems. Please indicate the extent to which you have experienced these symptoms or problems during the past week. Please answer by circling the number that best applies to you.

		Not at All	A Little	Quite a Bit	Very Much
31.	Could you eat solid food?	1	2	3	4
32.	Can you eat liquidised or soft food?	1	2	3	4
33.	Could you drink liquids?	1	2	3	4
34.	Have you had trouble with swallowing your saliva?	1	2	3	4
35.	Have you choked when swallowing?	1	2	3	4
36.	Have you had trouble enjoying your meals?	1	2	3	4
37.	Have you felt full up too quickly?	1	2	3	4
38.	Have you had trouble with eating?	1	2	3	4
39.	Have you had trouble eating in front of other people?	1	2	3	4
40.	Have you had a dry mouth?	1	2	3	4
41.	Have you had problems with your sense of taste?	1	2	3	4
42.	Have you had trouble with coughing?	1	2	3	4
43.	Have you had trouble with talking?	1	2	3	4
44.	Have you had acid indigestion or heartburn?	1	2	3	4
45.	Have you had trouble with acid or bile coming into your mouth?	1	2	3	4
46.	Have you had pain when you eat?	1	2	3	4
47.	Have you had pain in your chest?	1	2	3	4
48.	Have you had pain in your stomach?	1	2	3	4

© QLQ-OES18 Copyright 1999 EORTC Quality of life Group. All rights reserved

SAMPLE

APPENDIX VII

Dermatology Life quality Index

DLQI

Trial No. _______________ Initials: ___ ___ ___
Date of Birth (Day, Month, Year): __ __ / __ __ / __ __ __ __ Score: ☐
Today's date (Day, Month, Year): __ __ / __ __ / __ __ __ __

The aim of this questionnaire is to measure how much your skin problem has affected your life OVER THE LAST WEEK. Please tick ☐ one box for each question.

**1.**	Over the last week, how **itchy**, **sore**, **painful** or **stinging** has your skin been?	Very much<br>A lot<br>A little<br>Not at all	☐<br>☐<br>☐<br>☐
**2.**	Over the last week, how **embarrassed** or **self conscious** have you been because of your skin?	Very much<br>A lot<br>A little<br>Not at all	☐<br>☐<br>☐<br>☐
**3.**	Over the last week, how much has your skin interfered with you going **shopping** or looking after your **home** or **garden**?	Very much<br>A lot<br>A little<br>Not at all<br>Not relevant	☐<br>☐<br>☐<br>☐<br>☐
**4.**	Over the last week, how much has your skin influenced the **clothes** you wear?	Very much<br>A lot<br>A little<br>Not at all<br>Not relevant	☐<br>☐<br>☐<br>☐<br>☐
**5.**	Over the last week, how much has your skin affected any **social** or **leisure** activities?	Very much<br>A lot<br>A little<br>Not at all<br>Not relevant	☐<br>☐<br>☐<br>☐<br>☐
**6.**	Over the last week, how much has your skin made it difficult for you to do any **sport**?	Very much<br>A lot<br>A little<br>Not at all<br>Not relevant	☐<br>☐<br>☐<br>☐<br>☐
**7.**	Over the last week, has your skin prevented you from **working** or **studying**?	Yes<br>No<br>Not relevant	☐<br>☐<br>☐
	If "No", over the last week how much has your skin been a problem at **work** or **studying**?	A lot<br>A little<br>Not at all	☐<br>☐<br>☐
**8.**	Over the last week, how much has your skin created problems with your **partner** or any of your **close friends** or **relatives**?	Very much<br>A lot<br>A little<br>Not at all<br>Not relevant	☐<br>☐<br>☐<br>☐<br>☐

SAMPLE

**9.**	Over the last week, how much has your skin caused any **sexual difficulties**?	Very much	☐
		A lot	☐
		A little	☐
		Not at all	☐
		Not relevant	☐
**10.**	Over the last week, how much of a problem has the **treatment** for your skin been, for example by making your home messy, or by taking up time?	Very much	☐
		A lot	☐
		A little	☐
		Not at all	☐
		Not relevant	☐

Please check you have answered EVERY question. Thank you.

©AY Finlay, GK Khan, April 1992, This must not be copied without the permission of the authors.

SAMPLE

APPENDIX VIII

Your Current State of Health

Please consider your state of health <u>today</u> and tick one box for each question.

Q.1: Your mobility….

❒ I have no problems in walking about.

❒ I have some problems in walking about.

❒ I am confined to bed.

Q.2: Your self-care…

❒ I have no problems with self-care.

❒ I have some problems with washing or dressing myself.

❒ I am unable to wash or dress myself.

Q.3: Your usual activities…(e.g. work, study, housework, family or leisure activities)

❒ I have no problems with performing my usual activities.

❒ I have some problems with performing my usual activities.

❒ I am unable to perform my usual activities.

Q.4: Pain / Discomfort…

❒ I have no pain or discomfort.

❒ I have moderate pain or discomfort.

❒ I have extreme pain or discomfort.

Q.5: Anxiety / Depression…

❒ I am not anxious or depressed.

❒ I am moderately anxious or depressed.

❒ I am extremely anxious or depressed.

SAMPLE

How good or bad is your health today?

To help people say how good or bad a health state is, we have drawn a scale (like a thermometer) on which the best state you can imagine is marked 100 and the worst state you can imagine is marked 0. We would like you to indicate on this scale how good or bad your own health is today, in your opinion. Please do this by drawing a line from the box below to whichever point on the scale indicates how good or bad your health is today.

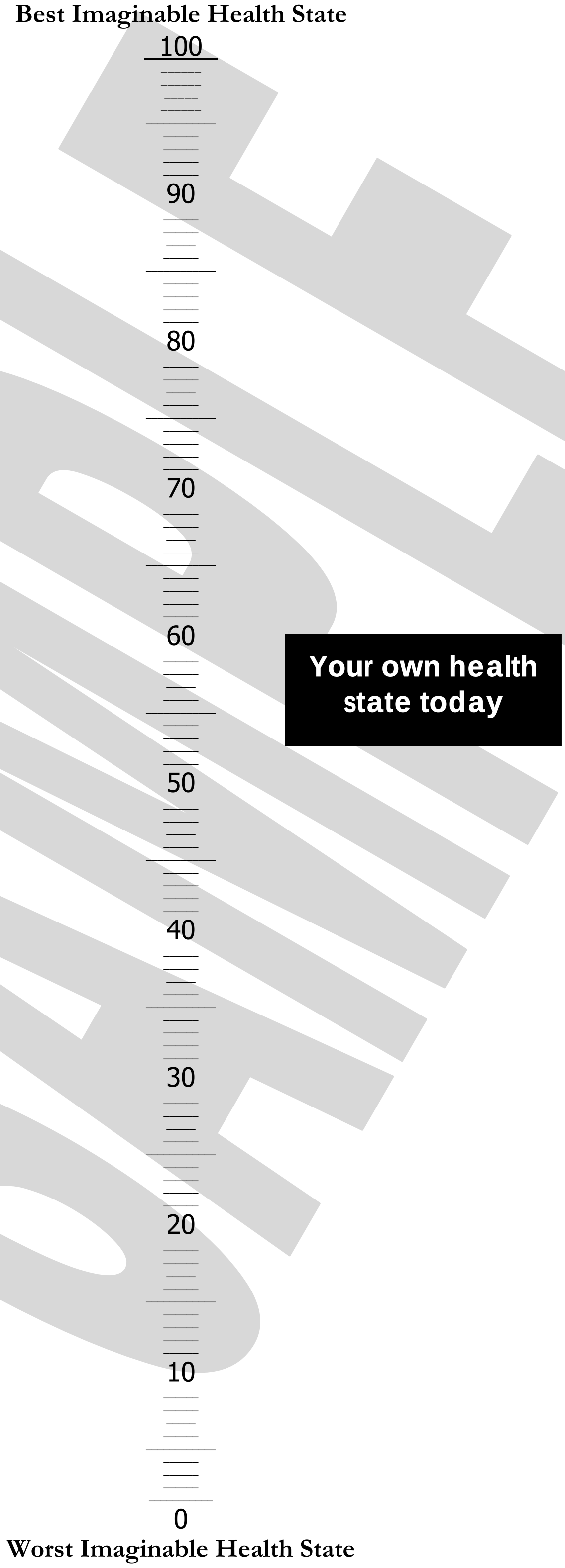

SAMPLE

APPENDIX IX

Collection, Preparation and Storage of Samples

Check that the patient has not withheld consent for blood, tissue and DNA samples to be collected and stored.

Blood Sample
To provide further information for translational research we request that a blood sample is stored for future research.

Blood sample is required

- Pre-treatment; this is most likely to be during the baseline assessment visit
- Post treatment at week 24 when the Endoscopic assessment and biopsy are carried out

At both time points collect blood into a 7mL EDTA (purple top) and 7mL serum (red top) tube according to local guidelines for use of vacutaners.

It is recommended that this sample is taken at the same time as the routine bloods as far as is possible so that the patient does not have to undergo an additional venapuncture.

- Allow the serum tube to clot for 30min prior to separation.
- Centrifuge the tubes at 2000g for 10 min.
- Aspirate the plasma/serum into 1ml aliquots (expected 6 aliquots, 3 of each).
- Retain the cells from the EDTA tube.
- Label each tube clearly with Patient Trial ID, Centre and Date of sample and whether serum or EDTA plasma. **Do not** include any other patient details such as name or DOB.
- Freeze the EDTA tube (containing cells) and the aliquots or serum and plasma immediately. Ideally at -80°C but -20°C is acceptable as an alternative.

Processing and freezing of the sample should ideally be carried out within 2 hours. It **MUST** be carried out on the same day.

Package the samples into dry ice and ship in batches to the Wales Cancer Bank using DHL. Collection of samples should be arranged through the WCTU.

For centres which are unable to separate blood, please use the sample packaging supplied following the packaging instructions. Post to the Wales Cancer Bank using the address below. Packaging will have been arranged at the time of centre set up. If additional packaging is required please contact the Trial Manager for SCOPE 1, at the WCTU on 029 2068 7459 or e-mail scope1@wctu.wales.nhs.uk.

Please enclose a sample tracking form with every sample which is sent whether by courier or by post. Fax a copy of the sample tracking form to WCTU the same day that the sample is sent. Please also enclose a copy of the T-SCOPE CRF.

Paraffin Blocks

Paraffin blocks are required

- Pre-treatment, the biopsy taken at diagnosis
- Post treatment at week 24 when the endoscopic assessment and biopsy are carried out
- During follow up only if a biopsy has been taken as a result of clinical decision

The paraffin blocks should be sent directly to the address below in Jiffy bags. Include only the Patient trial number, centre and date of sample. Patient name and hospital number should not be included.

Paraffin Blocks (and blood as applicable) should be sent to:

SCOPE 1
Wales Cancer Bank
Grove Mews
1 Coronation Road
Birchgrove
Cardiff
CF14 4QY

Pathology Reports should be sent directly to the Wales Cancer Trials Unit. Please note: the pathology report should be anonymous apart from the trial number. Patient details such as name and hospital number should be removed, and the patient trial number put on the form. Pathology reports should not be sent to the Wales Cancer Bank.

SCOPE 1
Wales Cancer Trials Unit
6th Floor, Neuadd Meirinnoydd
Heath Park,
Cardiff. CF14 4YS

APPENDIX X

Dysphagia Scale

0 Able to eat all solid food (no dysphagia)
1 Able to eat some but not all solids (dysphagia to some solids)
2 Able to swallow soft food only (dysphagia to all solids)
3 Able to swallow liquids only (dysphagia to solids and soft food)
4 Inability to swallow even saliva (complete dysphagia)

Adapted from:
Mellow MH, Pinkas H. 1985. Endoscopic Laser Therapy for Malignancies Affecting the Esophagus and Gastroesophageal Junction. Archives of Internal Medicine, 145:1443-6.

APPENDIX XI

CTCAE Toxicity criteria

Selected Common Terminology Criteria based on CTCAE Version 3.0

Short Name	1	2	3	4
**Allergic reaction**	Transient flushing or rash; drug fever <38°C (<100.4°F)	Rash; flushing; urticaria; dyspnea; drug fever ≥38°C (≥100°F)	Symptomatic broncospasm, with or without urticaria; parenteral medication(s) indicated; allergy-related oedema / angioedema; hypotension	Anaphylaxis
**Hearing (without monitoring program)**	-	Hearing loss not requiring hearing aid or intervention (i.e., not interfering with activities of daily living (ADL))	Hearing loss requiring hearing aid or intervention (i.e., interfering with ADL)	Profound bilateral hearing loss (>90 dB)
**Haemoglobin**	<LLN-10.0g/dL	<10.0 - 8.0g/dL	<8.0 – 6.5g/dL	<6.5g/dL
**Leukocytes (total wbc)**	<LLN - 3.0x10^9/L	<3.0x10^9/L - 2.0x10^9/L	<2.0x10^9/L - 1.0x10^9/L	<1.0x10^9/L
**Neutrophils**	< LLN - 1.5 x 10^9/L	< 1.5 – 1.0 x $10^{9/}$L	<1.0 – 0.5 x 10^9/L	<0.5 x 10^9/L
**Platelets**	< LLN - 75.0 x 10^9/L	< 75.0 – 50.0 x $10^{9/L}$	<50.0 – 25.0 x 10^9/L	<25.0 x 10^9/L
**Cardiac Ischemia/infarction**	Asymptomatic arterial narrowing without ischaemia	Asymptomatic and testing suggesting ischaemia; stable angina	Symptomatic and testing consistent with ischemia; unstable angina; intervention indicated	Acute myocardial infarction
**Hypertension**	Asymptomatic, transient (<24hrs) increase by > 20mmHg (diastolic) or to > 150/100 if previously WLN; intervention not indicated	Recurrent or persistent (>24hrs) or symptomatic increase by > 20mmHg (diastolic) or to >150/100 if previously WLN; monotherapy may be indicated	Requiring more than one drug or more intensive therapy than previously	Life-threatening consequences (e.g., hypertensive crisis)
**Left ventricular systolic dysfunction**	Asymptomatic, resting ejection fraction (EF) <60-50%; shortening fraction (SF) <30-24%	Asymptomatic, resting EF <50-40%<br>SF <24-15%	Symptomatic congestive heart failure (CHF) responsive to intervention.<br>EF <40—20% SF <15%	Refractory CHF or poorly controlled; EF <20%; intervention such as ventricular assist device, ventricular reduction surgery, or heart transplant indicated
**Pulmonary hypertension**	Asymptomatic without therapy	Asymptomatic, therapy indicated	Symptomatic hypertension, responsive to therapy	Symptomatic hypertension, poorly controlled

**INR (International Normalized Ratio of prothrombin time)**	>1 - 1.5 x ULN	>1.5 - 2 x ULN	> 2 x ULN	-
**PTT (Partial Thromboplastin Time)**	>1 - 1.5 x ULN	>1.5 - 2 x ULN	> 2 x ULN	-
**Fatigue**	Mild fatigue over baseline	Moderate or causing some difficulty performing some ADL	Severe fatigue interfering with ADL	Disabiling
**Weight Loss**	5 to <10% from baseline; intervention not indicated	10 - <20% from baseline; nutritional support indicated	≥ 20% from baseline; tube feeding or TPN indicated	
**Alopecia**	Thinning or patchy	Complete	-	-
**Injection site reaction / extravasation reaction**	Pain, itching, erythema	Pain or swelling, with inflammation or phlebitis	Ulceration or necrosis that is severe, operative intervention indicated	-
**Urticaria**	Intervention not indicated	Intervention indicated for <24 hours	Intervention indicated for ≥24 hrs	
**Wound complication, non-infectious**	Incisional separation of ≤25% of wound, no deeper than superficial fascia	Incisional separation of > 25% of wound with local care, asymptomatic hernia	Symptomatic hernia without evidence of strangulation; fascial disruption/dehiscence without evisceration; primary wound closure or revision by operative intervention indicated; hospitalization or hyperbaric O_2 therapy indicated	Symptomatic hernia with evidence of strangulation; fascial disruption with evisceration; major reconstruction flap, grafting, resection or amputation indicated
**Diarrhoea**	Increase of <4 stools per day over baseline; mild increase in ostomy output compared to baseline	Increase of 4-6 stools per day over baseline; IV fluids indicated <24 hrs; moderate increase in ostomy output compared to baseline; not interfering with ADL	Increase of ≥ 7 stools per day over baseline; incontinence; IV fluids <24 hrs; hospitalisation; severe increase in ostomy output compare to baseline; interfering with ADL	Life-threatening consequences (e.g. hemodynamic collapse)
**Dysphagia**	Symptomatic, able to eat regular diet	Symptomatic and altered eating/swallowing (e.g. altered dietary habits, oral supplements); IV fluids indicated <24 hrs	Symptomatic and severely altered eating/swallowing (e.g. inadequate oral caloric or fluid intake); IV fluids, tube feedings, or TPN indicated ≥24 hrs	Life-threatening consequences (e.g. obstruction, perforation)
**Oesophagitis**	Asymptomatic pathologic, radiographic, or endoscopic findings only	Symptomatic; altered eating/swallowing (e.g. altered dietary habits, oral supplements); IV fluids indicated <24 hrs	Symptomatic and severely altered eating/swallowing (e.g. inadequate oral calorific or fluid intake); IV fluids, tube feedings, or TPN indicated ≥ 24 hrs	Life threatening consequences

**Mucositis/ Stomatitis (clinical exam)**	Erythema of the mucosa	Patchy ulcerations or pseudomembranes	Confluent ulcerations or pseudomembranes; bleeding with minor trauma	Tissue necrosis; Significant spontaneous bleeding; life threatening consequences
**Mucositis/ Stomatitis (symptomatic)**	Minimal symptoms, normal diet	Symptomatic but can eat and swallow modified diet	Symptomatic and unable to adequately aliment or hydrate orally	Symptoms associated with life-threatening consequences
**Nausea**	Loss of appetite without alteration in eating habits	Oral intake decreased without significant weight loss, dehydration or malnutrition; IV fluids indicated <24hrs	Inadequate oral caloric or fluid intake; IV fluids, tube feedings or total parenteral nutrition (TPN) indicated ≥24 hrs	Life-threatening consequences
**Vomiting**	1 episode in 24 hours	2-5 episodes in 24 hours; IV fluids indicated < 24hours	≥ 6 episodes in 24 hours; IV fluids, or TPN indicated ≥ 24hrs	Life- threatening consequences
**Perforation GI (oesophagus, Stomach, duodenum, ileum, colon)**	Asymptomatic, radiographic finding only	Medical intervention indicated, IV fluids indicated < 24hrs	IV fluids, tube feedings, or TPN indicated ≥ 24hrs; operative intervention indicated	Life-threatening consequences
**Haemorrhage, GI**	Mild, intervention (other than iron supplements) not indicated	Symptomatic and medical intervention or minor cauterization indicated	Transfusion, interventional radiological, endoscopic or operative intervention indicated; radiation therapy (i.e., haemostasis of bleeding site)	Life- threatening consequences; major urgent intervention indicated
**ALT / AST**	>ULN – 2.5 x ULN	>2.5 – 5.0 x ULN	>5.0 – 20.0 x ULN	>20.0 x ULN
**Bilirubin**	>ULN – 1.5 x ULN	>1.5 – 3.0 x ULN	>3.0 – 10.0 x ULN	>10.0 x ULN
**Hypocalcemia**	<LLN – 2.0 mmol/L	<2.0 – 1.75 mmol/L	<1.75 – 1.5 mmol/L	<1.5 mmol/L
**Creatinine**	>ULN-1.5 x ULN	>1.5-3.0 x ULN	>3.0-6.0 x ULN	>6.0 x ULN
**Glomerular Filtration Rate**	<75-50% LLN	<50-25% LLN	<25% LLN, chronic dialysis not indicated	Chronic dialysis or renal transplant indicated
**Hypomagnesia**	<LLN – 0.5 mmol/L	<0.5 – 0.4 mmol/L	<0.4 – 0.3mmol/L	<0.3 mmol/L
**Proteinuria**	1+ or 0.15-1.0g.24hrs	2+ to 3+ or >1.0 - 3.5g/24hrs	4+ or >3.5g/24hr	Nephrotic syndrome
**Hyperuricemia**	>ULN – 10mg/dL ≥0.59 mmol/L without physiologic consequences		>ULN – 10mg/dL ≥0.59 mmol/L with physiologic consequences	>10mg/dL ≥0.59 mmol/L
**Dizziness**	With head movements or nystagmus only; not interfering with function	Interfering with function, but not interfering with ADL	Interfering with ADL	Disabling

**Bronchospasm**	Asymptomatic	Symptomatic not interfering with function	Symptomatic interfering with function	Life-threatening
**Cough**	Symptomatic, non-narcotic medication only indicated	Symptomatic and narcotic medication indicated	Symptomatic and significantly interfering with sleep or ADL	
**Dyspnea**	Dyspnea on exertion but can walk 1 flight of stairs without stopping	Dyspnea on exertion but unable to walk 1 flight of stairs or 1 city block (0.1km) without stopping	Dyspnea with ADL	Dyspnea at rest; intubation/ventilator indicated
**Cytokine release syndrome**	Mild reaction; infusion interruption not indicated; intervention not indicated	Requires therapy of infusion interruption but responds promptly to symptomatic treatment (e.g. antihistamines, NSAIDS, narcotics, IV fluids); prophylactic medications indicated for ≥24 hrs	Prolonged (i.e. not rapidly responsive to symptomatic medication and/or brief interruption of infusion); recurrence of symptoms following initial improvement; hospitalization indicate for other clinical sequelae (e.g. renal impairment, pulmonary infiltrates)	Life-threatening; pressor or ventilatory support indicated
**Tumour flare**	Mild pain not interfering with function	Moderate pain; pain or analgesics interfering with function, but not interfering with ADL	Severe pain; pain of analgesics interfering with function and interfering with ADL	Disabling
**Arterial vessel injury**	Asymptomatic diagnostic finding, intervention not indicated	Symptomatic (e.g. claudication) not interfering with ADL, repair or revision not indicated	Symptomatic interfering with ADL; repair or revision indicated	Life-threatening; disabling; evidence of end organ damage (e.g. CVA, MI, organ or limb loss)
**Thrombosis/ thrombus/ embolism**	-	Deep vein thrombosis or cardiac thrombosis; intervention (e.g., anticoagulation, lysis, filter, invasive procedure) not indicated	Deep vein thrombosis or cardiac thrombosis; intervention (e.g., anticoagulation, lysis, filter, invasive procedure) not indicated	Embolic event including pulmonary embolism or life threatening thrombus

NB: These are selected categories. For full list see http://ctep.cancer.gov/reporting/ctc.html

For all cases where applicable grade 5 is death

APPENDIX XII

RTOG/EORTC LATE RADIATION MORBIDITY SCORING SCHEMA

	0	1	2	3	4
Skin	None	Slight atrophy Pigmentation change Some hair loss	Patch atrophy; Moderate telangiectasia; Total hair loss	Marked atrophy; Gross telangiectasia	Ulceration
Subcutaneous Tissue	None	Slight induration (fibrosia) and loss of subcutaneous fat	Moderate fibrosis but asymptomatic Slight field contracture <10% linear reduction	Severe induration and loss of subcutaneous tissue Filed contracture >10% linear measurement	Necrosis
Mucous Membrane	None	Slight atrophy and dryness	Moderate atrophy and telangiectasia Little mucous	Marked atrophy with complete dryness Severe telangiectasia	Ulceration
Salivary Glands	None	Slight dryness of mouth Good response on stimulation	Moderate dryness of mouth Poor response on stimulation	Complete dryness of mouth No response on stimulation	Fibrosis
Spinal Cord	None	Mild L'Hermitte's syndrome	Severe L'Hermitte's syndrome	Objective neurological findings at or below cord level treated	Mono, para quadraplegia
Brain	None	Mild headache Slight lethargy	Moderate headache Great lethargy	Severe headaches Severe CNS dysfunction (partial loss of power or dyskinesia)	Seizures or paralysis Coma
Eye	None	Asymptomatic cataract Minor corneal ulceration or keratitis	Symptomatic cataract Moderate corneal ulceration Minor retinopathy or glaucoma	Severe keratitis Severe retinopathy or detachment Severe glaucoma	Panopthalmitis/ Blindness
Larynx	None	Hoarseness Slight arytenoid edema	Moderate arytenoid edema Chondritis	Severe edema Severe chondritis	Necrosis
Lung	None	Asymptomatic or mild symptoms (dry cough) Slight radiographic appearances	Moderate symptomatic fibrosis or pneumonitis (severe cough) Low grade fever Patchy radiographic appearances	Severe symptomatic fibrosis or pneumonitis Dense radiographic changes	Severe respiratory insufficiency Continuous oxygen Assisted ventilation
Heart	None	Asymptomatic or mild symptoms Transient T wave inversion & ST changes Sinus tachycardia >110 (at rest)	Moderate angina on effort Mild pericarditis Normal heart size Persistent abnormal T wave and ST changes Low ORS	Severe angina Pericardial effusion Constructive pericarditis Moderate heart failure Cardiac enlargement EKG abnormalities	Tamponade Severe heart failure Severe constrictive pericarditis
Oesophagus	None	Mild fibrosis	Unable to take solid	Severe fibrosis	Necrosis

	None	Slight difficulty in swallowing solids<br>No pain on swallowing	food normally<br>Swallowing semi-solid food<br>Dilation may be indicated	Able to swallow only liquids<br>May have pain on swallowing<br>Dilation required	Perforation<br>Fistula
Small / Large Intestine	None	Mild diarrhea<br>Mild cramping<br>Bowel movement 5 times daily<br>Slight rectal discharge or bleeding	Moderate diarrhea and colic<br>Bowel movement >5 times daily<br>Excessive rectal mucus or intermittent bleeding	Obstruction or bleeding<br>requiring surgery	Necrosis/ Perforation Fistula
Liver	None	Mild lassitude<br>Nausea, dyspepsia<br>Slightly abnormal liver function	Moderate symptoms<br>Some abnormal liver function tests<br>Serum albumin normal	Disabling hepatitic insufficiency<br>Liver function tests grossly abnormal<br>Low albumin<br>Edema or ascites	Necrosis/ Hepatic coma or encephalopathy
Kidney	None	Slight epithelial atrophy<br>Minor telangiectasia (microscopic hematuria)	Moderate frequency<br>Generalized telangiectasia<br>Intermittent macroscopic hematuria	Severe frequency and dysuria<br>Severe generalized telangiectasia (often with petechiae)<br>Frequent hematuria<br>Reduction in bladder capacity (<150 cc)	Necrosis/ Contracted bladder (capacity <100 cc)<br>Severe hemorrhagic cystitis
Bladder	None	Slight epithelial atrophy<br>Minor telangiectasia (microscopic hematuria)	Moderate frequency<br>Generalized telangiectasia<br>Intermittent macroscopic hematuria	Severe frequency and dysuria<br>Severe generalized telangiectasia (often with petechiae)<br>Frequent hematuria<br>Reduction in bladder capacity (<150 cc)	Necrosis/ Contracted bladder (capacity <100 cc)<br>Severe hemorrhagic cystitis
Bone	None	Asymptomatic<br>No growth retardation<br>Reduced bone density	Moderate pain or tenderness<br>Growth retardation<br>Irregular bone sclerosis	Severe pain or tenderness<br>Complete arrest of bone growth<br>Dense bone sclerosis	Necrosis/<br>Spontaneous fracture
Joint	None	Mild joint stiffness<br>Slight limitation of movement	Moderate stiffness<br>Intermittent or moderate joint pain<br>Moderate limitation of movement	Severe joint stiffness<br>Pain with severe limitation of movement	Necrosis/ Complete fixation

GUIDELINES from http://www.rtog.org/members/toxicity/late.html

All toxicities Grade 3, 4 or 5* must be verified by the Principal Investigator.

*ANY TOXICITY WHICH CAUSED DEATH IS GRADED 5.

APPENDIX XIII

RECIST criteria

Response Evaluation Criteria In Solid Tumours

Eligibility

Only patients with measurable disease at baseline should be included in protocols where objective tumor response is the primary endpoint.

Measurable disease - the presence of at least one measurable lesion. If the measurable disease is restricted to a solitary lesion, its neoplastic nature should be confirmed by cytology/histology.
Measurable lesions - lesions that can be accurately measured in at least one dimension with longest diameter ≥20 mm using conventional techniques or ≥10 mm with spiral CT scan.
Non-measurable lesions - all other lesions, including small lesions (longest diameter <20 mm with conventional techniques or <10 mm with spiral CT scan), i.e., bone lesions, leptomeningeal disease, ascites, pleural/pericardial effusion, inflammatory breast disease, lymphangitis cutis/pulmonis, cystic lesions, and also abdominal masses that are not confirmed and followed by imaging techniques; and.

- All measurements should be taken and recorded in metric notation, using a ruler or calipers. All baseline evaluations should be performed as closely as possible to the beginning of treatment and never more than 4 weeks before the beginning of the treatment.

The same method of assessment and the same technique should be used to characterize each identified and reported lesion at baseline and during follow-up.
Clinical lesions will only be considered measurable when they are superficial (e.g., skin nodules and palpable lymph nodes). For the case of skin lesions, documentation by color photography, including a ruler to estimate the size of the lesion, is recommended.

Methods of Measurement

CT and MRI are the best currently available and reproducible methods to measure target lesions selected for response assessment. Conventional CT and MRI should be performed with cuts of 10 mm or less in slice thickness contiguously. Spiral CT should be performed using a 5 mm contiguous reconstruction algorithm. This applies to tumours of the chest, abdomen and pelvis. Head and neck tumours and those of extremities usually require specific protocols.

Lesions on chest X-ray are acceptable as measurable lesions when they are clearly defined and surrounded by aerated lung. However, CT is preferable.

When the primary endpoint of the study is objective response evaluation, ultrasound (US) should not be used to measure tumour lesions. It is, however, a possible alternative to clinical measurements of superficial palpable lymph nodes, subcutaneous lesions and thyroid nodules. US might also be useful to confirm the complete disappearance of superficial lesions usually assessed by clinical examination.

The utilization of endoscopy and laparoscopy for objective tumour evaluation has not yet been fully and widely validated. Their uses in this specific context require sophisticated equipment and a high level of expertise that may only be available in some centres. Therefore, the utilization of such techniques for objective tumour response should be restricted to validation purposes in specialized centres. However, such techniques can be useful in confirming complete pathological response when biopsies are obtained.

Tumour markers alone cannot be used to assess response. If markers are initially above the upper normal limit, they must normalize for a patient to be considered in complete clinical response when all lesions have disappeared.

Cytology and histology can be used to differentiate between PR and CR in rare cases (e.g., after treatment to differentiate between residual benign lesions and residual malignant lesions in tumour types such as germ cell tumours).

Baseline documentation of “Target” and “Non-Target” lesions
All measurable lesions up to a maximum of five lesions per organ and 10 lesions in total, representative of all involved organs should be identified as target lesions and recorded and measured at baseline.
Target lesions should be selected on the basis of their size (lesions with the longest diameter) and their suitability for accurate repeated measurements (either by imaging techniques or clinically).
A sum of the longest diameter (LD) for all target lesions will be calculated and reported as the baseline sum LD. The baseline sum LD will be used as reference by which to characterize the objective tumor.
All other lesions (or sites of disease) should be identified as non-target lesions and should also be recorded at baseline. Measurements of these lesions are not required, but the presence or absence of each should be noted throughout follow-up.
Response Criteria

	Evaluation of target lesions
* Complete Response (CR):	Disappearance of all target lesions
* Partial Response (PR):	At least a 30% decrease in the sum of the LD of target lesions, taking as reference the baseline sum LD
* Progressive Disease (PD):	At least a 20% increase in the sum of the LD of target lesions, taking as reference the smallest sum LD recorded since the treatment started or the appearance of one or more new lesions
* Stable Disease (SD):	Neither sufficient shrinkage to qualify for PR nor sufficient increase to qualify for PD, taking as reference the smallest sum LD since the treatment started
	Evaluation of non-target lesions
* Complete Response (CR):	Disappearance of all non-target lesions and normalization of tumor marker level
* Incomplete Response/ Stable Disease (SD):	Persistence of one or more non-target lesion(s) or/and maintenance of tumor marker level above the normal limits
* Progressive Disease (PD):	Appearance of one or more new lesions and/or unequivocal progression of existing non-target lesions (1)

Although a clear progression of “non target” lesions only is exceptional, in such circumstances, the opinion of the treating physician should prevail and the progression status should be confirmed later on by the review panel (or study chair).

Evaluation of best overall response

The best overall response is the best response recorded from the start of the treatment until disease progression/recurrence (taking as reference for PD the smallest measurements recorded since the treatment started). In general, the patient's best response assignment will depend on the achievement of both measurement and confirmation criteria

Target lesions	Non-Target lesions	New Lesions	Overall response
CR	CR	No	CR
CR	Incomplete response/SD	No	PR
PR	Non-PD	No	PR
SD	Non-PD	No	SD
PD	Any	Yes or No	PD
Any	PD	Yes or No	PD
Any	Any	Yes	PD

Patients with a global deterioration of health status requiring discontinuation of treatment without objective evidence of disease progression at that time should be classified as having “symptomatic deterioration”. Every effort should be made to document the objective progression even after discontinuation of treatment.
In some circumstances it may be difficult to distinguish residual disease from normal tissue. When the evaluation of complete response depends on this determination, it is recommended that the residual lesion be investigated (fine needle aspirate/biopsy) to confirm the complete response status.

Confirmation

The main goal of confirmation of objective response is to avoid overestimating the response rate observed. In cases where confirmation of response is not feasible, it should be made clear when reporting the outcome of such studies that the responses are not confirmed.

To be assigned a status of PR or CR, changes in tumor measurements must be confirmed by repeat assessments that should be performed no less than 4 weeks after the criteria for response are first met. Longer intervals as determined by the study protocol may also be appropriate.

In the case of SD, follow-up measurements must have met the SD criteria at least once after study entry at a minimum interval (in general, not less than 6-8 weeks) that is defined in the study protocol

Duration of overall response

The duration of overall response is measured from the time measurement criteria are met for CR or PR (whichever status is recorded first) until the first date that recurrence or PD is objectively documented, taking as reference for PD the smallest measurements recorded since the treatment started.

Duration of stable disease

SD is measured from the start of the treatment until the criteria for disease progression are met, taking as reference the smallest measurements recorded since the treatment started.

The clinical relevance of the duration of SD varies for different tumor types and grades. Therefore, it is highly recommended that the protocol specify the minimal time interval required between two measurements for determination of SD. This time interval should take into account the expected clinical benefit that such a status may bring to the population under study.

Response review

For trials where the response rate is the primary endpoint it is strongly recommended that all responses be reviewed by an expert(s) independent of the study at the study's completion. Simultaneous review of the patients' files and radiological images is the best approach.

Reporting of results

All patients included in the study must be assessed for response to treatment, even if there are major protocol treatment deviations or if they are ineligible. Each patient will be assigned one of the following categories: 1) complete response, 2) partial response, 3) stable disease, 4) progressive disease, 5) early death from malignant disease, 6) early death from toxicity, 7) early death because of other cause, or 9) unknown (not assessable, insufficient data).

All of the patients who met the eligibility criteria should be included in the main analysis of the response rate. Patients in response categories 4-9 should be considered as failing to respond to treatment (disease progression). Thus, an incorrect treatment schedule or drug administration does not result in exclusion from the analysis of the response rate. Precise definitions for categories 4-9 will be protocol specific.

All conclusions should be based on all eligible patients.

Sub analyses may then be performed on the basis of a subset of patients, excluding those for whom major protocol deviations have been identified (e.g., early death due to other reasons, early discontinuation of treatment, major protocol violations, etc.). However, these sub analyses may not serve as the basis for drawing conclusions concerning treatment efficacy, and the reasons for excluding patients from the analysis should be clearly reported.

The 95% confidence intervals should be provided.

From: http://ctep.cancer.gov/forms/quickrcst.doc

Wales Cancer Trials Unit

Contact Details

6th Floor, Neuadd Meirionnydd,
Heath Park
Cardiff
CF14 4YS

General Telephone Number 029 2068 7500

Email: SCOPE1@WCTU.wales.nhs.uk

Visit our website – www.wctu.org.uk
