# Supplementary material for: Quality assurance of the SCOPE 1 trial in oesophageal radiotherapy
Source: Radiat Oncol. 2017 Nov 15;12:179. doi: 10.1186/s13014-017-0916-7 (PMC5688711; doi:10.1186/s13014-017-0916-7)
Supplement: Supplementary file 2 — SCOPE1 Trial Radiotherapy Procedures Document. (PDF 2300 kb) [file 13014_2017_916_MOESM2_ESM.pdf]

SCOPE 1

Radiotherapy Treatment Planning and Delivery

SCOPE 1: Study of Chemoradiotherapy in Oesophageal Cancer Plus or Minus Erbitux

A randomised phase II/III multi-centre clinical trial of definitive chemo-radiation, with or without Cetuximab, in carcinoma of the oesophagus

RADIOTHERAPY GUIDANCE and PROCEDURES
Version 3.0, 9th July 2008
This document should be read in conjunction with the SCOPE 1 protocol (EUDRACT No. 2006-002241-37, ISRCTN: 47718479)

SPONSOR: Velindre NHS Trust

Authorised by:

Name:	Dr Tom Crosby	Role:	Chief Investigator
Signature:	[signature]	Date:	15/7/8
Name:	Gareth Griffiths	Role:	Scientific Director WCTU
Signature:	[signature]	Date:	16/7/08

WCTU WALES CANCER TRIALS UNIT UNED YMCHWIL CANSER CYMRU
YMDDIRIEDOLAETH GIG FELINDRE
VELINDRE NHS TRUST
CANCER RESEARCH UK
NCRI National Cancer Research Institute

SCOPE 1 Radiotherapy Guidance and Procedures

Radiotherapy Treatment Planning and Delivery

Abbreviations and Glossary 3

1 Introduction 4

1.1 General Requirements 4

2 Patient Positioning and Computerised Tomography (CT) Planning Scan Acquisition 5

2.1 Timing of the Planning Scan 5
2.2 Treatment Position 5
2.3 System of Reference 5
2.4 Use of IV Contrast 5
2.5 Extent of the Scan 7

3 Organs at risk (OAR) 8

3.1 Lungs 8
3.2 Spinal Cord 8
3.3 Heart 8
3.4 Liver 8
3.5 Kidneys 8

4 Definition of Treatment Volumes 9

4.1 GTV Definition 9
4.2 CTV Definition 9
4.3 PTV Definition 11

5 Dose Volume Guidelines 12

5.1 PTV 12
5.2 Spinal Cord 12
5.3 Lungs 13
5.4 Heart 13
5.5 Liver 13
5.6 Kidneys 13

6 Pre-Treatment Verification 14

7 On- Treatment Verification 15

7.1 On-Treatment Verification and Adjustment of the Isocentre Position 15
7.2 On-Treatment Verification of Patient Outline 16

Appendix 1: Delineation of Heart Volume....18

Appendix 2: Suggested System to Reduce Posterior CTV Margin Where Required 21

Appendix 3: Planning and Optimisation of the Single-Phase External Beam Plan.........23

A3.1: Introduction.... 23
A3.2: Beam Orientations.... 23
A3.3: Positioning of Lateral / Oblique Beams 23
A3.4: Choice of Collimator Angle and Shielding Orientation for Lateral / Oblique Beams. 24
A3.5: Establishing the Contribution of Each Beam 25
A3.6: Coverage of the PTV 26
A3.7: Fine Tuning the Plan 26
A3.8: The Final Plan 26

Appendix 4: Problem Examples 28

A4.1: Introduction to Problem Examples.... 28
A4.2: Relative contribution of each beam.... 29
A4.3: Variations in lateral beam wedging 30
A4.4: Use of filler beams to lower hotspots within the PTV 31
A4.5: Use of opposing superior – inferior wedged beams to improve coverage of 95% isodose and dose homogeneity within the PTV.... 32

Abbreviations and Glossary

CT	Computerised tomography
CTV	Clinical Treatment Volume
DVH	Dose Volume Histogram
EUS	Endoscopic ultrasound
FSD	Focus to surface distance
GOJ	Gastro-oesophageal junction
GTV	Gross Tumour Volume
ICRU	International Commission on Radiation Units and Measurements
IRMER	The Ionising Radiation (Medical Exposure) Regulations
OAR	Organs at Risk
PET	Positron Emission Tomography
PRV	Planning Risk Volume
PTV	Planning Treatment Volume
RCR	The Royal College of Radiologists
TMG	Trial Management Group
WCTU	Wales Cancer Trials Unit

1 Introduction

This document describes the process for radiotherapy treatment planning of oesophageal cancer and has been developed for the purpose of the SCOPE 1 trial. The aim is to aid the delivery of high quality radiotherapy and to allow quality assurance procedures to be applied to ensure this is achieved. However, some aspects of the process are not explicitly defined and will vary according to the capabilities and characteristics of each centre and to some extent their local practice and experience. Guidelines for the delivery of concurrent chemotherapy are described elsewhere.

1.1 General Requirements

Conformal radiotherapy with a pixel based inhomogeneity correction is essential. The type of dose calculation algorithm to be used is not specified; the guidelines for Planning Treatment Volume (PTV) coverage are given later in section 5.1 and are based on use of a pencil beam algorithm. It is recognised however that these guidelines may not be realistic for other algorithms. Centres should assess dose coverage to PTV according to local experience in this case. Photon energy should be between 6MV and 10MV (but energies in excess of 10MV should only be used in exceptional cases due to secondary build-up depth). A combination of energies is permissible. Final calculation grid spacing of no greater than 0.3cm is recommended.

2 Patient Positioning and Computerised Tomography (CT) Planning Scan Acquisition

2.1 Timing of the Planning Scan

The planning scan should be performed within 2 weeks of starting the neo-adjuvant phase of chemotherapy AND within 6 weeks of the staging CT scan.

2.2 Treatment Position

Patients should be planned and treated in the supine position with both arms above their heads. Immobilisation of the patient using a 'chest board' which fixes the arm positions above the head is recommended and immobilisation of the legs with a device similar to a 'knee-fix' is also encouraged.

2.3 System of Reference

For the CT planning scan a suitable system of reference must be used. Three transversely aligned tattoos marked at the right, anterior and left surfaces of the patient (tattoos correspond to radio-opaque markers held in place for the duration of the CT planning scan) enable the patient to be correctly aligned for treatment. A single reference mark (for example the anterior mark) may be used to reference the isocentre of the external beam plan.

2.4 Use of IV Contrast

Use of IV contrast is recommended in line with RCR Recommendations[1]. Where contrast-enhanced CT planning scans are to be used for the dose calculation there may be an effect on the monitor unit calculation, which will not be representative of the treatment situation. The magnitude of this effect will vary between individual patients, scanning protocols and centres. There are three acceptable solutions:

1. Use of single contrast-enhanced scan only. This can be used if the centre is satisfied that there are no implications of using contrast, for example if it has performed a study to assess the dosimetric effects of using contrast.
2. Use of both contrasted-enhanced and non-contrast CT scans. The contrast-enhanced scan is used for target volume definition and fused with the non-contrast scan which is used for dose calculation.
3. Use of single contrast-enhanced scan and assignation of unit density to heavily contrasted areas.

---

[1]Board of the Faculty of Clinical Radiology, The Royal College of Radiologists (2005) *Standards For Iodinated Intravascular Contrast Agent Administration To Adult Patients* Royal College of Radiologists, London. http://www.rcr.as.uk

2.4.1 Additional issues to consider with the use of IV contrast enhanced radiotherapy planning scans

1. **Safety:** It is strongly recommended that each centre develops its own working instructions for the delivery of IV contrast for radiotherapy planning scans. It is also recommended that the RCR document 'Standards For Iodinated Intravascular Contrast Agent Administration To Adult Patients' is read and followed. The following should be considered:
    a. Patient identification:
        i. Ensure that the correct patient is scanned following the correct protocol.
        ii. Ensure consent form for this procedure is completed
    b. Allergic reaction to contrast:
        i. Awareness of features associated with increased risk of reaction: history of allergy or asthma. Ask if patient has had previous contrast-enhanced imaging
        ii. Awareness of medical support: a member of the medical team should be contactable throughout the duration of the scan and the emergency drugs trolley should be brought round to the scanner or be easily accessible.
    c. Effects of contrast in renal insufficiency; ensure recent creatinine is available and that patient is not dehydrated. Note that risks are increased in elderly patients, patients with cardiac failure, and diabetics (especially if taking oral metformin)
        i. If serum creatinine level is above 120μmol/l but less than 150micromol/l, a member of the medical team should be informed and contrast administered at their discretion.
        ii. If serum creatinine level is above 150μmol/l, a member of the medical team should be informed and contrast should not be administered, unless patient is on dialysis that will take place within 24hrs.

    If in doubt, the decision should be made by a member of the medical team and if necessary discussed with a member of the TMG. Any reactions should be included in the patient's notes, and reported to the WCTU.

2. **Practical procedure for IV contrast:**
    a. Insert cannula
    b. Position patient in radiotherapy position
    c. Select correct imaging protocol; consider requirement for pre and post- contrast image acquisition
    d. **Optimise image quality with IV contrast:** These are recommendations based on experience from three centres, who have kindly allowed us to review their clinical protocols: Mount Vernon Hospital, Royal Marsden NHS Trust, and Velindre NHS Trust. Centres need to be aware that these recommendations are from clinical experience with their own hardware and software, and that some degree of local development may be required.
        i. Type: Either Omnipaque or Visipaque can be used
        ii. Temperature: ensure contrast is brought to room temperature by placing in warm water

   iii. Volume: 100ml
   iv. Infusion rate: 2.5-3mls per sec. This may be reduced to as low as 1ml per sec, depending on cannula size
   v. Time between injection and CT: 35-40s

e. Remove cannula at completion of scan

2.5 Extent of the Scan

To enable accurate assessment of the dose to organs at risk the scan should extend superiorly to at least one CT slice above the apices of the lungs and inferiorly to the iliac crest (L2). Scans for upper third tumours may need to extend superiorly to the tragus. Slice thickness should be no greater than 0.5cm.

3 Organs at risk (OAR)

3.1 Lungs

The full extent of the right and left lungs are to be outlined, this should be done in such a way that the planning system will be able to calculate a combined lung Dose Volume Histogram (DVH).

3.2 Spinal Cord

The spinal cord should be outlined on slices which include or are within 2cm of the PTV in the superior and inferior directions. A Planning Risk Volume (PRV) for the cord is created to account for positioning error. The size of the margin added to the cord being commensurate with the accuracy of treatment delivery expected and as such, the tolerance level allowed in portal image verification on treatment. For example, Velindre Hospital applies a margin of 0.5cm isotropically to generate the spinal cord PRV and allows no more than 0.5cm movement of the isocentre on treatment before corrective action is taken. See section 7.1

3.3 Heart

The whole heart should be outlined to the extent of the pericardial sac (if visible). The major blood vessels (superior to the organ) and the inferior vena cava (towards the inferior extent of the heart) are excluded. Appendix 1 contains an example.

3.4 Liver

The whole liver should be outlined if the level of its superior edge overlaps with the level of the inferior extent of the PTV.

3.5 Kidneys

Each kidney should be outlined separately if the level of its superior edge overlaps with the level of the inferior extent of the PTV.

4 Definition of Treatment Volumes

Targets are defined following the principles of ICRU 50 and 62. The spinal cord and spinal cord PRV (section 3.2) should be outlined prior to definition of the treatment volumes. Dialogue with a specialist Upper GI radiologist regarding the target volume definition is encouraged.

4.1 GTV Definition

The Gross Tumour Volume (GTV) is the gross primary and nodal disease as defined on the planning CT scan with all available diagnostic information. This should include as a minimum: Endoscopic ultrasound (EUS) and contrast-enhanced CT. Some centres may have access to Positron Emission Tomography (PET) scans and their use is encouraged. In some cases the EUS will not define the full extent of the disease because the scope has failed to pass through the stricture, in such cases information is used from the original endoscopy and the diagnostic CT scan.

The GTV is localised on axial slices of the planning CT scan using the EUS to define a reference point (tracheal carina or the arch of the aorta) as well as the superior and inferior extent of the GTV. In principle the GTV should encompass the disease as defined on any of the above imaging modalities used (i.e. CT, EUS and/or PET), even if it’s only apparent on a single modality.

4.2 CTV Definition

The Clinical Treatment Volume (CTV) is defined differently in two clinical situations depending on the proximity of the GTV to the gastro-oesophageal junction (GOJ). Tumours that have a high risk of disease within the stomach have the potential to metastasise to lymph nodes that will only be included within the CTV if specifically outlined. Thus, two CTV definition protocols are defined below.

4.2.1 CTV Definition for a GTV which does not extend to within 2cm of the gastro-oesophageal junction.

The CTV will comprise the GTV plus a margin of up to 1cm laterally and 2cm superiorly and inferiorly (along the line of the oesophagus). It is created in three steps:

- Step 1: The GTV is copied and labelled ‘CTVA’ (so that the GTV is preserved as a separate structure) and extended by 2cm superiorly and inferiorly by manually drawing along the line of the oesophagus.

- Step 2: CTVA is copied and labelled ‘CTVB’. It is grown by a margin of 1cm in the right, left, anterior and posterior directions.

- Step 3: CTVB may then be adjusted as follows. Assessment of CTVB's proximity to the spinal cord PRV is made and where it is deemed that the spinal cord dose volume constraints will not be met, the posterior margin may be reduced. This reduction should be performed on a slice-by-slice basis and should be subject to a minimum CTVA to CTVB margin of 0.5cm. This is discussed in more detail in Appendix 2.

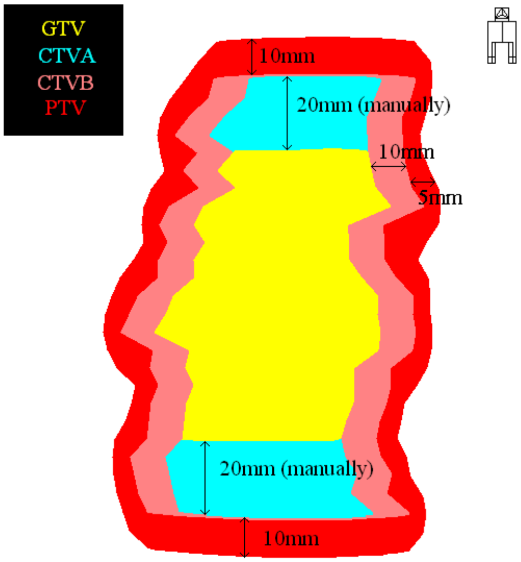

4.2.2 CTV Definition for a GTV which involves or comes within 2cm of the gastro-oesophageal junction.

The CTV will comprise the GTV plus a margin of up to 1cm laterally, 2cm superiorly (along the line of the oesophagus) and 2 cm inferiorly (this will include the mucosa of the stomach in the direction of the lymph node stations along the lesser curve including the para-cardial and left gastric lymph nodes). It is created in five steps:

- Step 1: The GTV is copied and labelled 'CTVA' (so that the GTV is preserved as a separate structure) and extended by 2cm superiorly by manual drawing along the line of the oesophagus.

- Step 2: CTVA is extended inferiorly as far as the GOJ making note of the length of extension from the GTV to this point. This step is not necessary if the GTV already extends to the level of the GOJ.

- Step 3: CTVA is copied and labelled 'CTVB'. This is grown with a margin of 1cm in the right, left, anterior and posterior directions.

- Step 4: CTVB is manually extended inferiorly such that the total inferior extension from GTV to CTVB is 2cm. This extension should aim to include the mucosa of the stomach,

in the direction of the lymph node stations along the lesser curve including the para-cardial and left gastric lymph nodes.

- Step 5: CTVB may then be adjusted as follows. Assessment of CTVB's proximity to the spinal cord PRV is made and where it is deemed that the spinal cord dose volume constraints will not be met, the posterior margin may be reduced. This reduction should be performed on a slice-by-slice basis and should be subject to a minimum CTVA – CTVB margin of 0.5cm. This is discussed in more detail in Appendix 2.

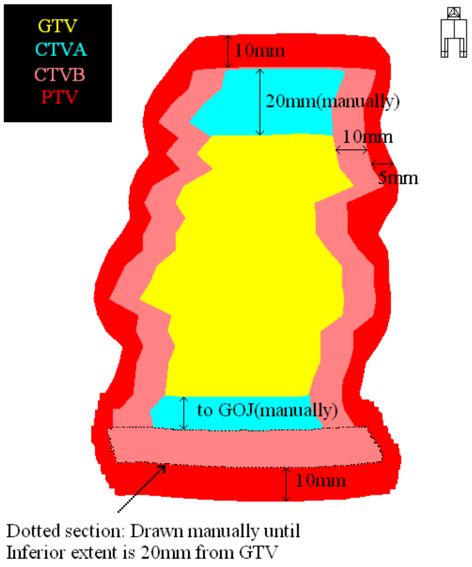

4.3 PTV Definition

CTVB is copied and labelled 'PTV'. It is created by the addition of the following margins:

Superiorly and inferiorly: 1.0cm
Laterally, anteriorly and posteriorly: 0.5cm (this margin is applied in all circumstances regardless of the proximity of the target to the spinal cord)

The maximum treatment field length is 17cm, i.e. maximum EUS disease length of primary tumour and lymph nodes is 10cm (assumes approximate 1cm extension from PTV to field length).

5 Dose Volume Guidelines

There follows a set of 'dose volume guidelines.' The guidelines for the PTV (section 5.1) should be taken as definitive (subject to the type of dose calculation algorithm used), however the aim of the remaining guidelines (sections 5.2 – 5.6) is to assist optimisation of the plan in consideration of all the organs at risk. These guidelines should all be achievable in the majority of cases and therefore allow for the plan to be tailored to the individual case such that the patients' associated co-morbidity may be considered and doses to organs at risk modified within the guidelines given.

Region of Interest / Organ at Risk	Dose Constraint
PTV	V95% (47.5Gy) > 99.0%
PTV	PTV min > 93% (46.5Gy)
DMAX	<107% (53.5Gy)
GTV	GTV min > 100% (50.0Gy)
Spinal Cord PRV	Cord Max <80% (40Gy)
Combined Lungs	V40% (V20Gy) <25%
Heart	V80% (V40Gy) < 30%
Liver	V60% (V30Gy) < 60%
Individual kidneys	V40% (V20Gy) <25%

5.1 PTV

The aim is to encompass the PTV with the 95% isodose with the best possible conformality of the 95% isodose to the PTV. In practice 99% of the PTV should be covered by the 95% isodose. The PTV maximum should be no more than 107% of the prescribed dose to the ICRU reference point – this maximum dose is determined in accordance with ICRU definitions whereby a region of dose is considered clinically meaningful if its minimum diameter exceeds 1.5cm. These requirements for the PTV are based on the use of a pencil beam algorithm – if any other algorithm is used centres should assess dose coverage to PTV according to local experience of best possible coverage achievable.

5.2 Spinal Cord

Dose to the spinal cord PRV should be increased to a level of around 38Gy and up to a maximum point dose of 40Gy. In practice this is achieved via a high posterior beam contribution and results in best possible reduction of doses to heart & lungs. No significant advantage is expected in attempting to reduce the dose to spinal cord below 38Gy, conversely increasing the dose to this safe level will give improved dose volume results for the remaining OARs.

5.3 Lungs

The volume of lung (right and left combined) receiving 20Gy should be less than 25% i.e. V20 < 25%. This level of dose may be achieved by judicious choice of gantry angle, optimised shielding and by limiting the percentage contribution of the lateral / lateral oblique beams.

5.4 Heart

The volume of heart receiving 40Gy should be less than 30% i.e.V40 < 30%. A proportion of the heart may overlap with the PTV – dose reduction to the remainder of the heart volume if required may be achieved by reducing the anterior beam contribution.

5.5 Liver

The volume of liver receiving 30Gy should be less than 60% i.e. V30 < 60%.

5.6 Kidneys

Volume of each kidney receiving 20Gy should be less than 25% i.e.V20 < 25%. Where this is not achieved the plan should aim to spare one kidney (subject to consideration of individual kidney function as demonstrated on a renogram) as far as possible and within the V20 < 25% limit. This is not expected to be a problem except in some with Siewert Type 3 tumours (which are not eligible for SCOPE 1).

6 Pre-Treatment Verification

Prior to treatment commencing, centres should follow their local protocols as regards pre-treatment verification; this may include a simulator check of the treatment, where any adjustments required should also be made according to local practice. Where simulator checks are performed, the single phase technique reduces this to one rather than two procedures.

An alternative system verifies positional accuracy on the first fraction of treatment. This may be done via the generation of 'dummy' isocentre fields (shown below) in the treatment planning system. These fields are prepared with a standard field size with zero collimator twist and are positioned at the anterior (gantry = 0°) and lateral (gantry = 90º or 270º) orientations to enable translation of any shifts seen in the comparative verification images into movements in the anterior-posterior, right–left and superior-inferior directions.

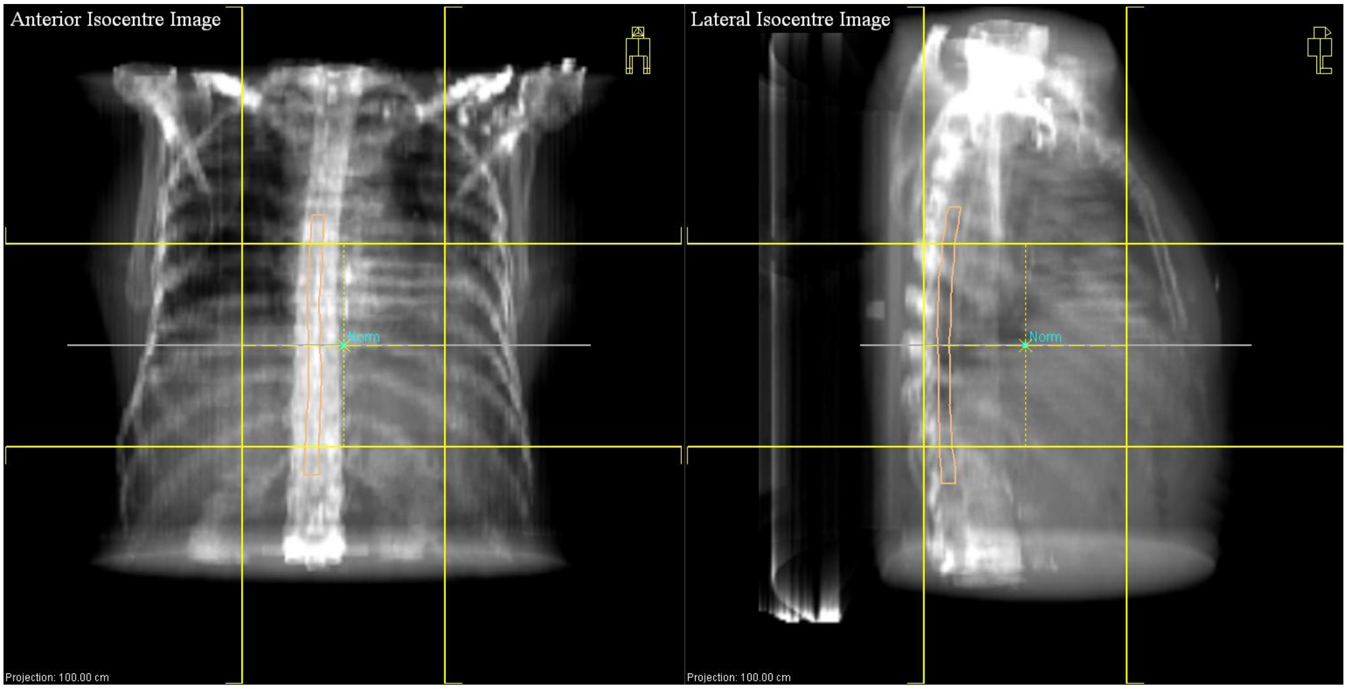

The 'dummy fields' are exported to the treatment 'record and verification' software and are used as 'reference images' with which 'on-treatment' portal images may be compared (see section 7). As such, the treatment is verified on set and a pre-treatment simulator check is not performed.

7 On-Treatment Verification

7.1 On-Treatment Verification and Adjustment of the Isocentre Position

Accuracy of delivery should be verified on the treatment set. This may be managed according to local protocols and in accordance with best practice. Our standard protocol for treatment positional verification specifies the collection of electronically acquired lateral and anterior portal isocentre images (or films where electronic means are not available) on the first three days of treatment and thereafter on a weekly basis.

Images are analysed via anatomical matching as shown in the images below: for the anterior image the vertebral bodies are outlined and matched, for the lateral image the process is repeated using the vertebral bodies and the sternum. The magnitude and direction of displacement between the 'reference' and 'on-treatment' images is then measured in the Sup-Inf, Ant-Post and Left-Right directions.

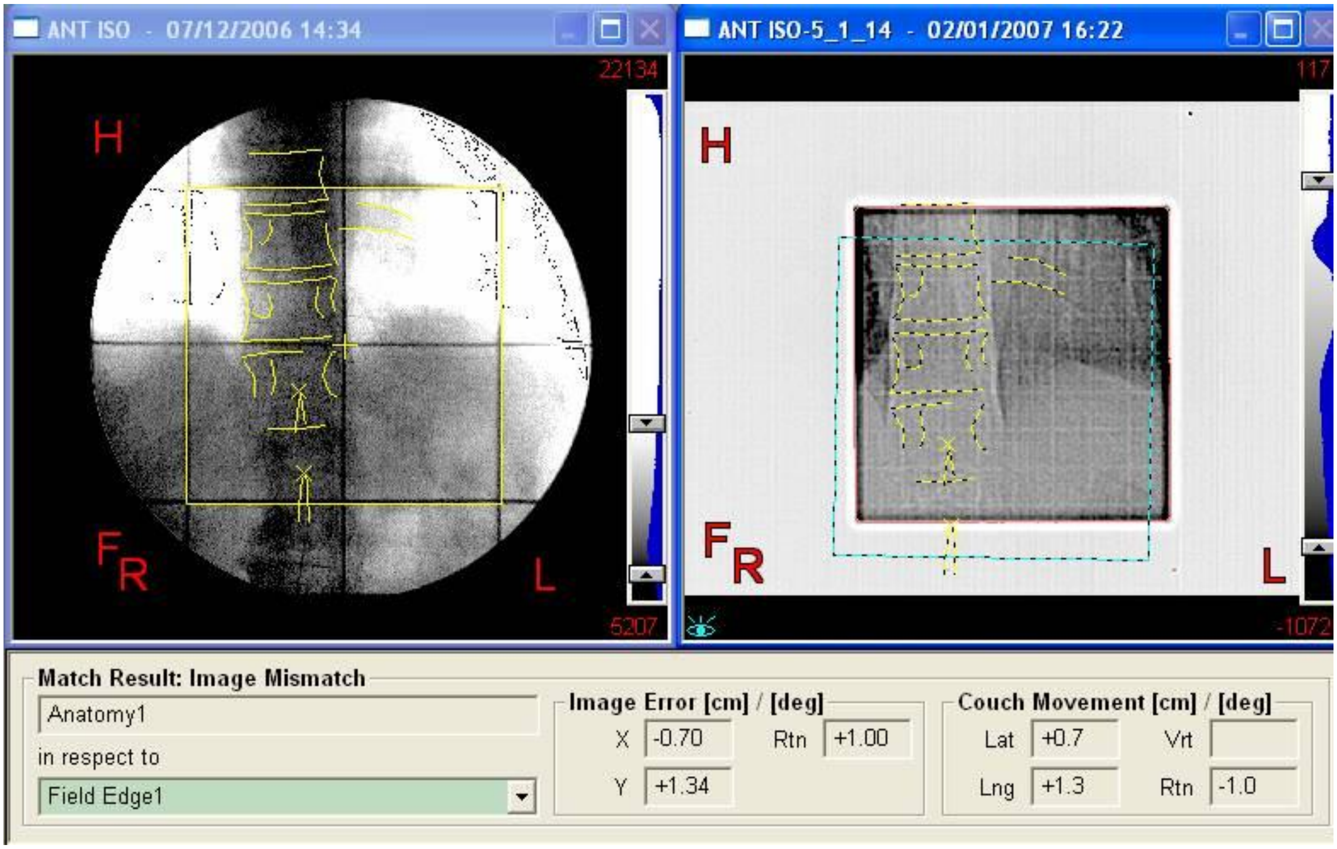

This analysis is performed and checked by suitably trained and authorised 'IRMER practitioners' For images which fall outside the accepted tolerance, action is determined based on the extent of the mismatch - three action levels are defined as follows:

- Level 1

  No action is required if a portal image is less than 0.5cm displaced from the reference image.

- Level 2

  If any given portal image is between 0.5 and 1.0cm displaced from the reference image then treatment may continue but set up is monitored by repeating portal images on each subsequent fraction until three sets have been acquired. At this point the average shift is calculated and the set up movements of the isocentre from the patient reference point are adapted accordingly. An off-line correction strategy is adopted i.e. a further set of images is taken on the next fraction of treatment and is analysed prior to the next day's treatment.

- Level 3

  If any given portal image is more than 1.0cm displaced from the reference image, then the treatment set up is reviewed prior to further treatment being delivered. This review may be a simulator or an on-set on-line correction strategy. i.e. prior to set up, new set up isocentre movements from the patient reference mark are calculated using the known displacements from the reference image and a further set of isocentre images must be taken and analysed before the treatment is delivered.

All changes are fully documented within the patient treatment record.

7.2 On-Treatment Verification of Patient Outline

Accuracy of the patient outline should be verified on set, as changes may have occurred since the CT Planning scan was acquired. This may be done according to local protocols and will depend on the technology available at each centre. Our standard protocol requires the measurement of FSD for each treatment field for the first three fractions of treatment. The readings should reflect a consistent treatment set up i.e. if set up instructions are changed at any point during the treatment, the measurements must be taken for the three fractions following the change. Where the average reading for any field is more than 0.5cm out of tolerance, the treatment sheet is referred to Physics and the need for monitor unit correction is assessed. However if any single measurement for any given beam is more than 1.5cm different from that planned, then the matter is investigated before further treatment is delivered.

Where the patient's plan is referred for possible monitor unit correction the following guidelines are applied: If any single beams' average FSD reading is more than 1.0cm different from the planned FSD, or if any two beams' average FSD is more than 0.5cm different from that planned, then a monitor unit correction is calculated, checked and applied. Large changes in FSD (>1.5cm) may be indicative of poor set up and are investigated further to confirm treatment accuracy before a monitor unit correction is applied.

Appendix 1: Delineation of Heart Volume

The whole heart is outlined to the extent of the pericardial sac (if visible). The major blood vessels (superior to the organ) and the inferior vena cava (towards the inferior extent of the heart) are excluded. The superior extent is often difficult to define and may be simplified by identification of the vessels superior to the heart. We use the point where the pulmonary trunk and the right pulmonary artery are seen as separate structures as indication of the superior extent of the heart. Shown below are alternate CT images for a scan taken at 0.3cm intervals.

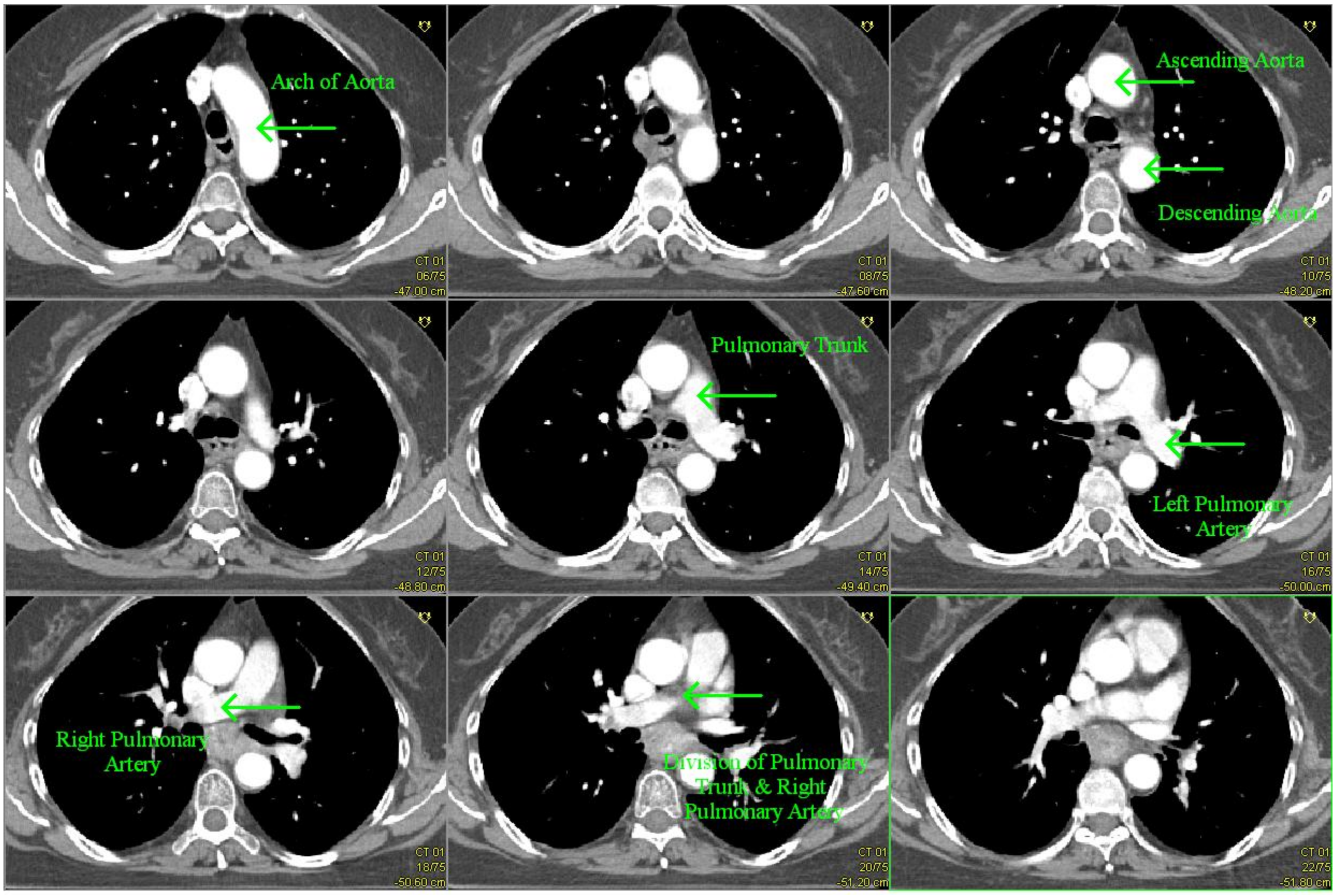

The definition of the heart is shown below on the same data set. The superior extent of the heart has been interpreted as the 1st section on which the right and left pulmonary arteries have separated. Throughout the heart is outlined to the extent of the pericardial sac. The inferior extent is less problematic to delineate as the organ appears well defined compared to the surrounding tissues in the abdomen. However, if possible, the inferior vena cava should be excluded.

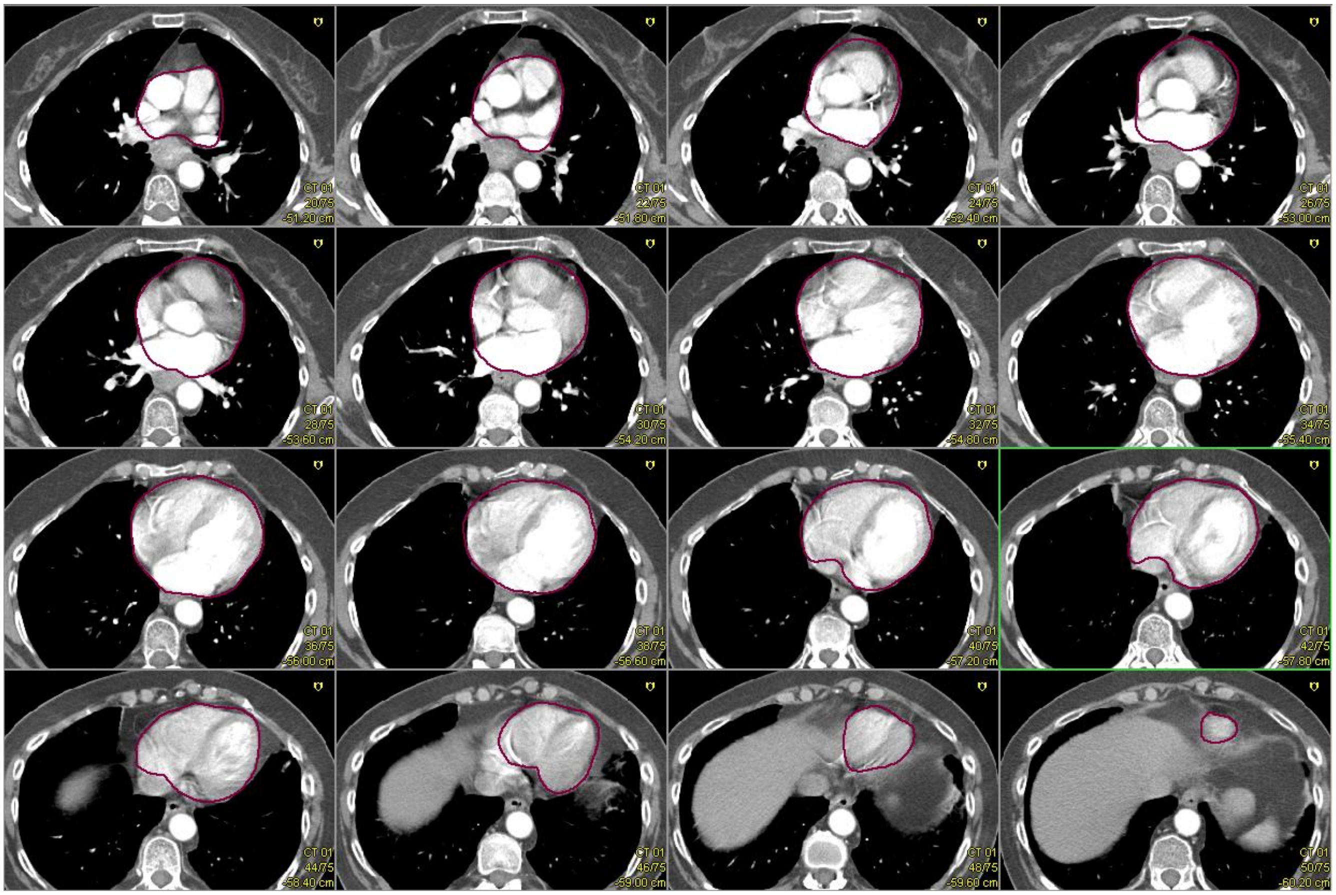

For reference the non-delineated CT data set is also provided below.

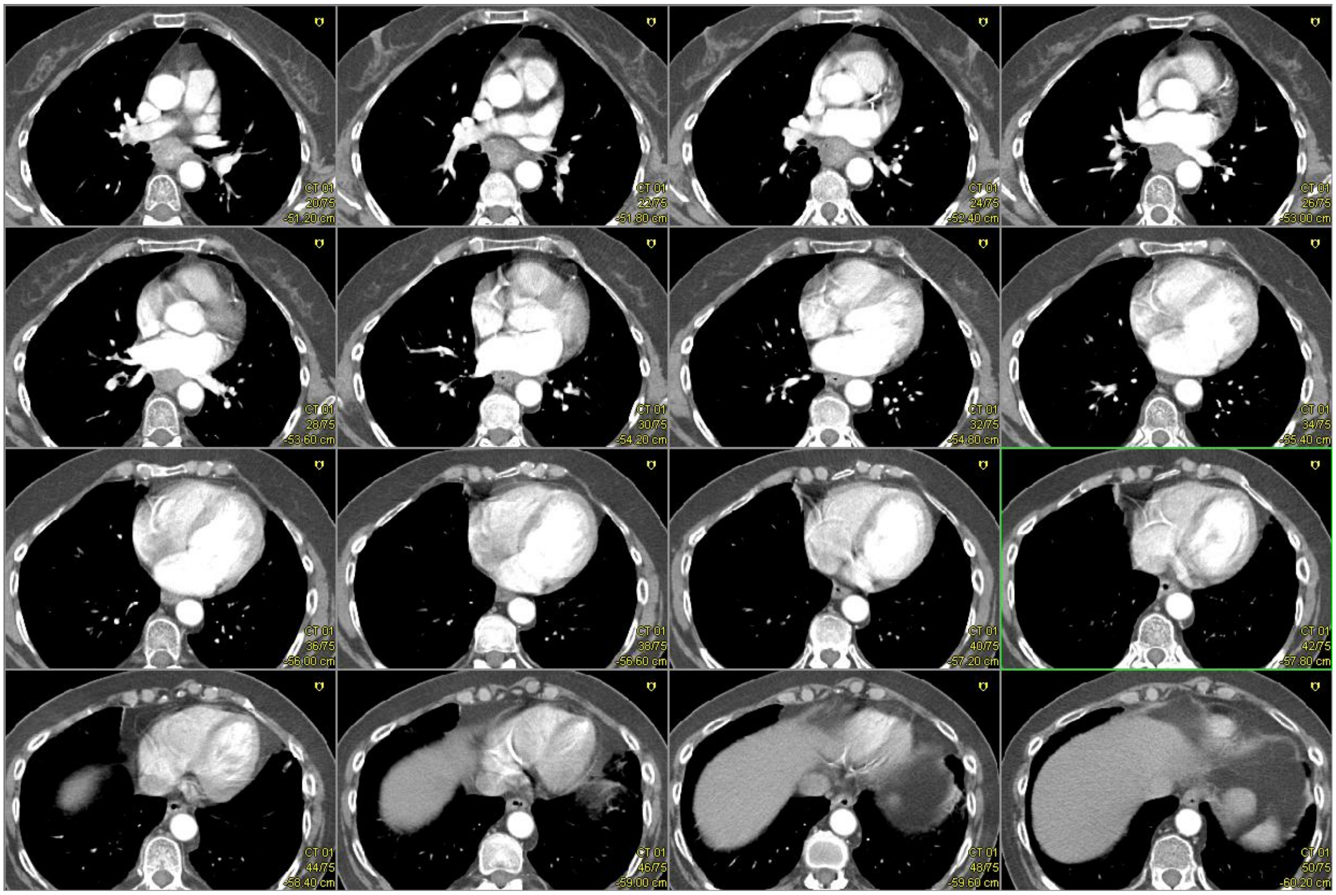

Appendix 2: Suggested System to Reduce Posterior CTV Margin, Where Required

Where the PTV is estimated to be so close to the spinal cord PRV that its dose volume constraints cannot be met, we recommend reducing the posterior extension of CTVA to CTVB (from a maximum of 1cm to a minimum of 0.5cm). This process may be performed according to local centres' discretion yet this example gives a system of adjustment which enables the clinician to delineate PTVs for which the dose volume constraints are able to be achieved.

The distance between CTVB and the spinal cord PRV that will allow the cord dose volume constraints to be met is estimated (NB an additional margin is added to CTVB during creation of the PTV). Practically this cannot be easily defined, but should be evaluated at a local level with a pragmatic approach. Once the optimal distance (for each centre) between CTVB and spinal cord PRV is established, it can be applied on an individual patient basis. This distance will then be maintained by adjusting the CTVA to CTVB posterior margin on a slice-by-slice basis as follows:

Following the creation of CTVB, the distance between its posterior edge and the anterior edge of the spinal cord, PRV should be measured. At Velindre, we have noted that if CTVB lies no closer than 1.3cm from the spinal cord PRV, at any point then the CTVA to CTVB margin is kept at 1cm. However, if CTVB lies <1.3cm from the spinal cord PRV then its posterior margin is reduced on a slice-by-slice basis to a minimum 0.5cm. We recommend creating two guide structures ('Min Post CTV' and 'Max Post CTV').
Min Post CTV = CTVA + 1.0cm right, left and anteriorly and a 0.5cm margin posteriorly.
Max Post CTV = CTVA + 1.0cm right, left, anteriorly and posteriorly (i.e. the original CTVB).
CTVB is then adjusted within the range of these guide structures on a slice-by-slice to maintain the 1.3cm distance. An example of this process is shown opposite.

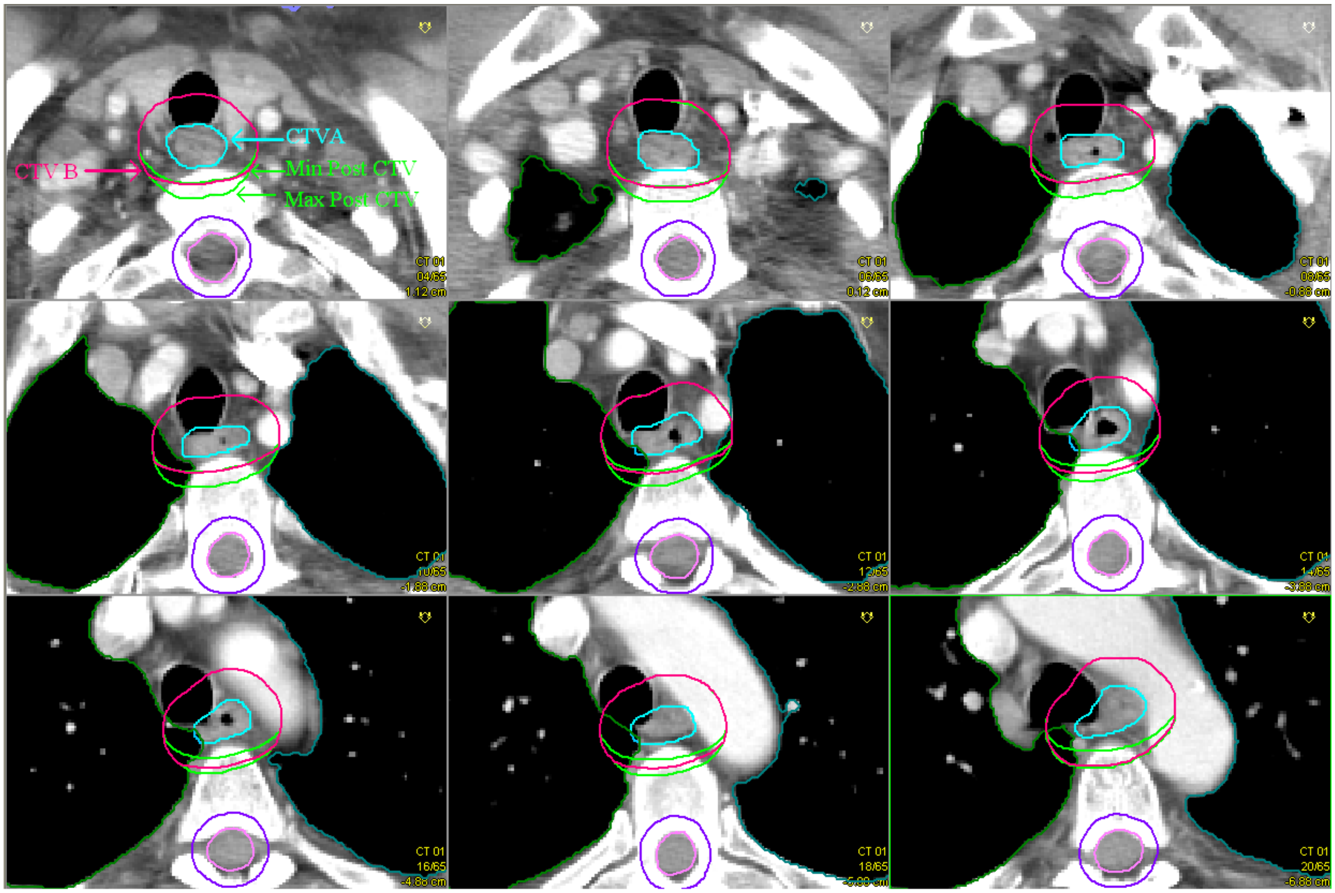
CTVA
CTV B
Min Post CTV
Max Post CTV

Appendix 3: Planning and Optimisation of the Single-Phase External Beam Plan

A3.1: Introduction

The purpose of this section is to offer guidance to centres who have not had working experience, of using a single phase technique and as such comprises a ‘walk-through’ example which is not intended to be prescriptive but aims to offer assistance if required. Single-phase planning had been demonstrated to offer significant cardiac dose reduction as compared to a two-phase approach (Ant-Post phase, followed by 3-field plan). Furthermore, the single-phase may offer a radiobiological advantage to organs at risk and a reduced resource requirement as compared with the two-phase approach.

A3.2: Beam Orientations

The exact number of beams and gantry angles are not explicitly defined – each patient will require an optimised arrangement. It is up to the individual centres to decide the beam arrangement. However, four primary beam orientations: anterior, posterior, right lateral (or lateral oblique) and left lateral (or lateral oblique), are usually satisfactory. This section describes how an unproblematic patient plan may be produced. Appendix 4 explores methods for planning more difficult cases.

A3.3: Positioning of Lateral / Oblique Beams

These beams should be positioned firstly to avoid the spinal cord PRV. The optimal gantry angle may be selected using the beams eye view to assess where the greatest gap between the Cord PRV and the PTV is achieved. Where possible, the gantry angle which gives the narrowest apparent size of the PTV should be considered in order to spare normal tissue.

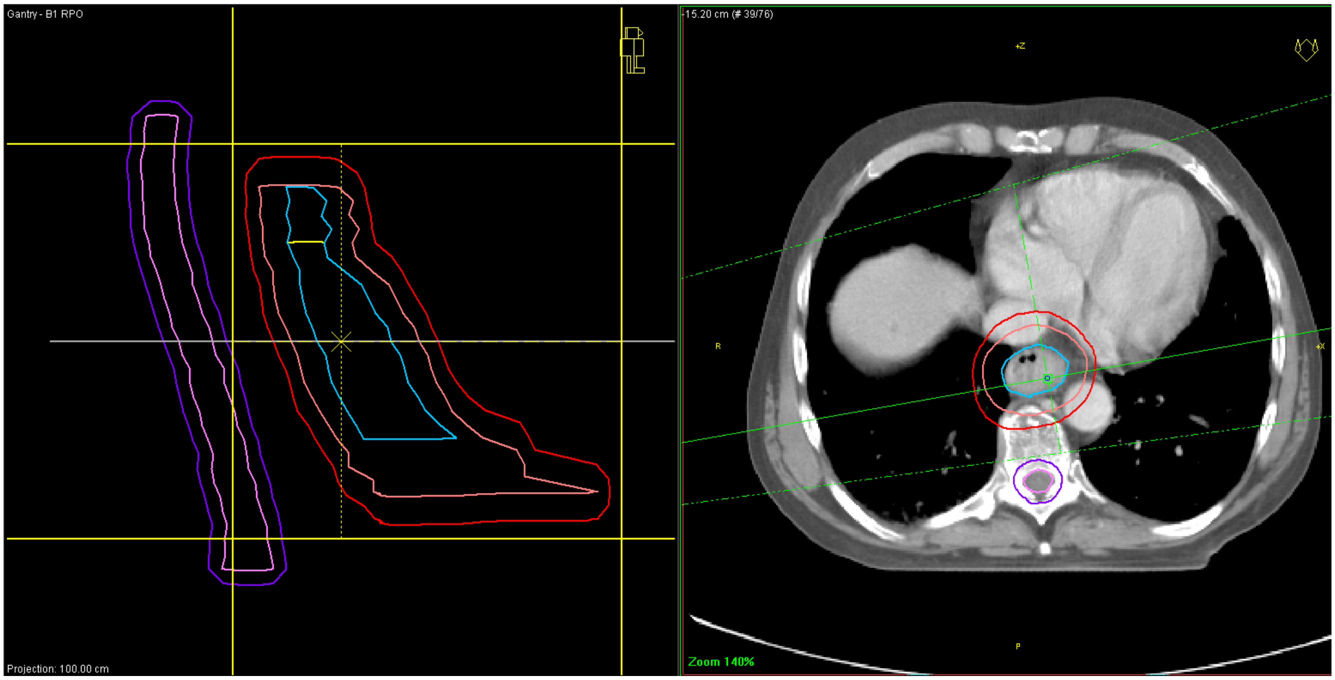

Care should be taken to avoid gantry angles which may cause beams to pass through the dense structural parts of the treatment couch panel. We have experienced such problems with the use of the Varian tennis racket panel as the presence of the posterior beam requires that the open part of the panel be used – this part of the panel contains side bars which have significant attenuation and should be avoided during treatment. This may be avoided if the elements of the couch panel are visualised in the planning system as shown below. Here the position and size of the side bars of the treatment couch panel have been measured and added to the patient data set (assumes the patient is positioned centrally on the treatment couch). With these in place, the limiting posterior extent of the lateral/oblique beams can easily be assessed and so problems avoided.

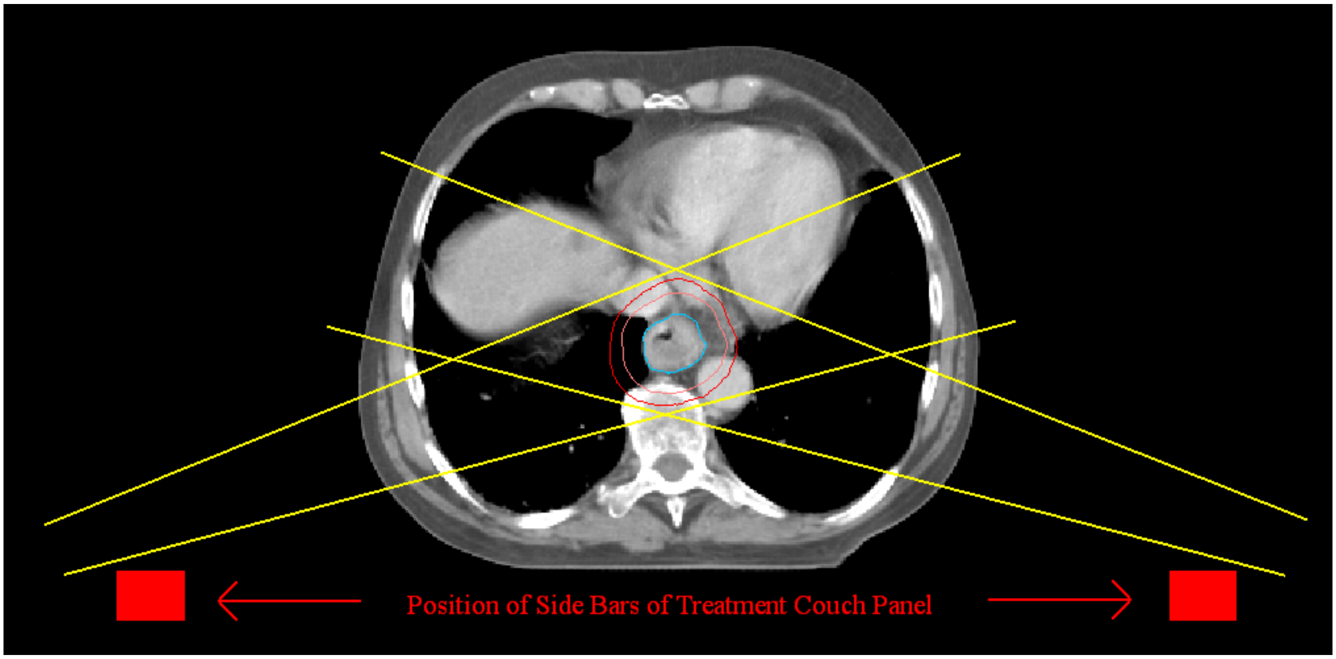

A3.4: Choice of Collimator Angle and Shielding Orientation for Lateral / Oblique Beams.

The usual 'curved' shape of the PTV from the lateral / oblique view will mean that the preferred collimator orientation for MLC shielding will often be around 0 degrees (that which enables the most conformal shielding considering the limitation of fixed MLC leaves and given their low resolution as compared to conformal shielding blocks). This position may be assessed as a starting point and approximate shielding added. For the single phase approach the lateral/oblique beams will frequently not require a wedge oriented in the ant-post direction which (if varian EDWs are used for example) would require the collimator angle to be rotated by 90 degrees. For other treatment delivery options, such as fixed or flying wedges etc. or conformal shielding blocks, this situation may not arise. However, it is undesirable to extend the treatment delivery process by their use if EDWs and MLCs are available. Where dynamic wedging is needed in the ant-post direction, the example in Appendix 4 (A4.2) shows how the loss of conformality to the PTV may be minimised.

A3.5: Establishing the Contribution of Each Beam

With the lateral / oblique beams in place, the anterior and posterior beams are added with approximate shielding and a crude calculation can then be done in order to assist in setting initial percentage contributions from each beam. At this point, the balance between the four beams can be adjusted so that the cord dose approaches the guideline of 38Gy (76% of the prescriptive dose) and that the remaining dose volume guidelines are met. The guidelines, if easily met will then allow for the plan to be tailored to the individual case such that the patient's associated co-morbidity may be considered and doses to organs at risk modified within the given guidelines. This is shown in the figures below and with reference to the dose volume guidelines given in section 5. For this example, the cord dose approaches the guideline as recommended, the lung dose volume guideline has been achieved and the heart and liver doses are considerably lower than the guideline level.

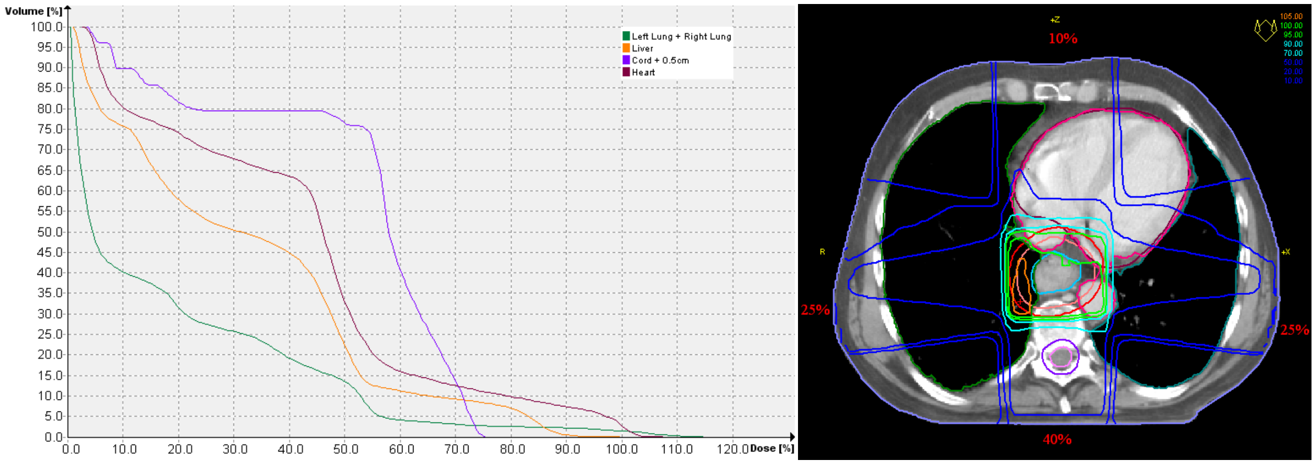

By way of example, if the patient were known to have significantly poor lung function we have the freedom within the process to increase the dose to the liver and heart in favour of the lung. This scenario is shown below.

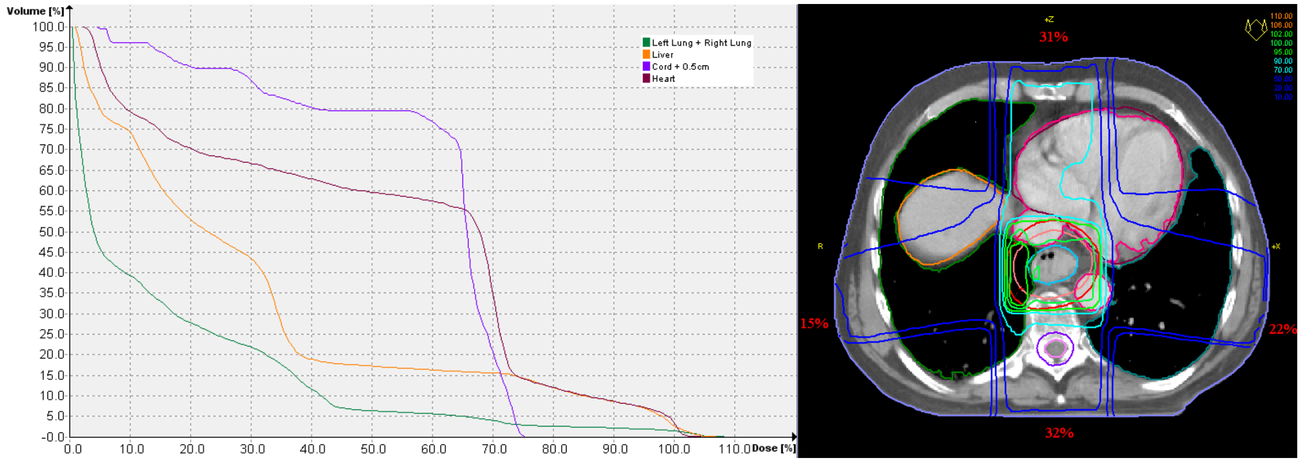

A3.6: Coverage of the PTV

PTV coverage and the level of dose homogeneity within the PTV is assessed. In order to achieve the dose-volume requirements for the PTV given in section 5.1 the inclusion of wedges (and in some cases additional beams) may be deemed appropriate at this point. Adjustment to the relative contributions of each beam may follow. The DVH should again be referenced to check acceptability of doses to OARs.

A3.7: Fine Tuning the Plan

At this point a more detailed calculation is carried out (grid spacing of no greater than 0.3cm is recommended) and assessment of the overall plan is made. Subsequently MLC positions may be adjusted in order to improve the conformality of the 95% isodose to the PTV, and any of the beam parameters previously established may be modified over a number of iterations in order to fully optimise the plan.

A3.8: The Final Plan

The final plan meets the dose volume requirements for the PTV and has also achieved a suitable balance of doses to normal tissue within the guidelines given. The plan has also taken into account the associated co-morbidities of the individual patient. The table below summarises the dose-volume information for this plan.

Region of Interest / Organ at Risk	Dose Constraint	Achieved
PTV	V95% (47.5Gy) > 99%	99.2%
PTV	PTV min > 93% (46.5Gy)	93.4%
DMAX	<107% (53.5Gy)	106%
Spinal Cord PRV	Cord Max <80% (40Gy)	75.1%
Combined Lungs	V40% (V20Gy) <25%	12%
Heart	V80% (V40Gy) < 30%	13%
Liver	V60% (V30Gy) < 60%	16%
Individual kidneys	V40% (V20Gy) <25%	N/A

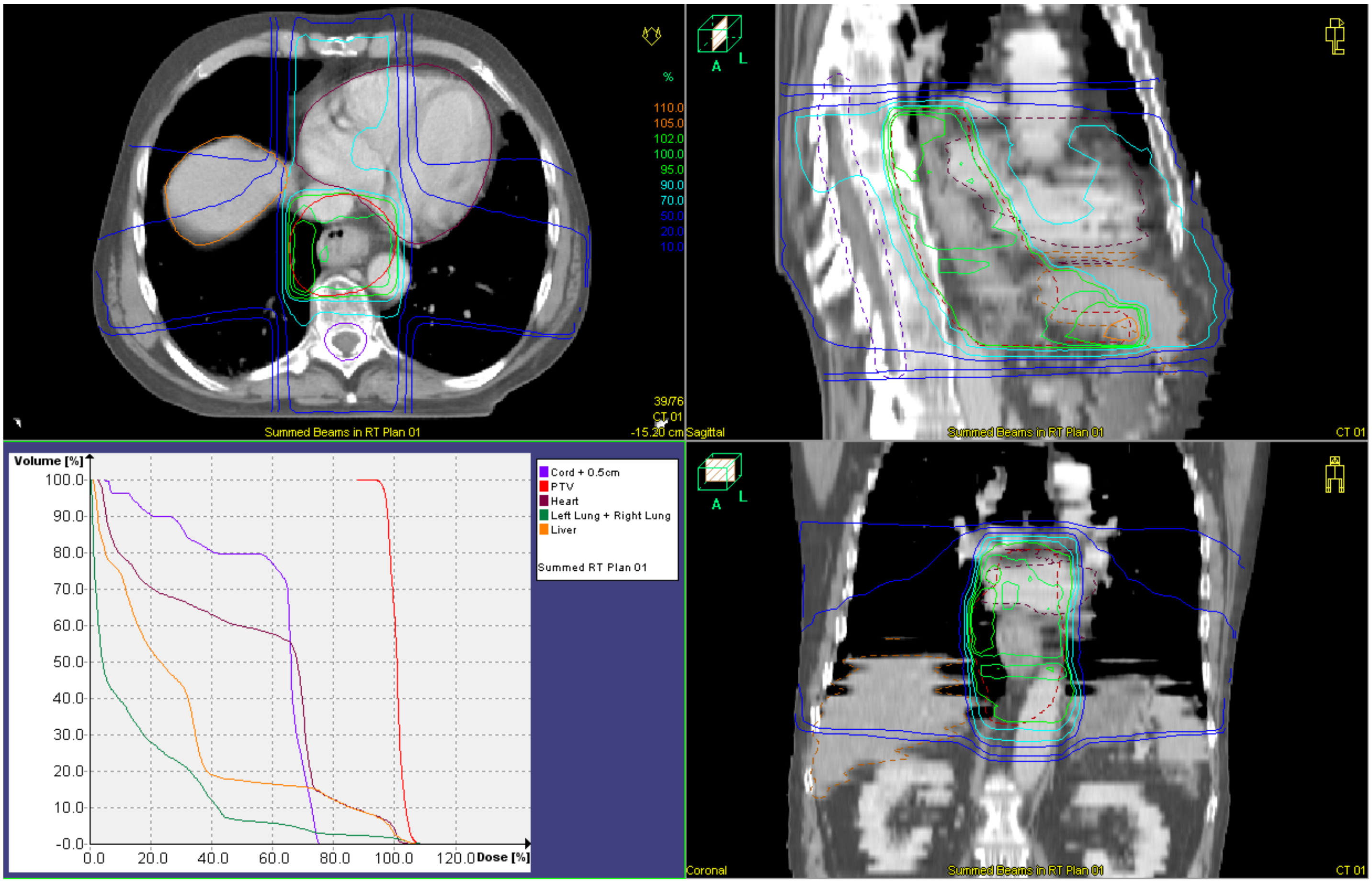
%
110.0
105.0
102.0
100.0
95.0
90.0
70.0
50.0
20.0
10.0
39/76
CT 01
-15.20 cm
Summed Beams in RT Plan 01
Sagittal
Summed Beams in RT Plan 01
CT 01
A
L
Volume [%]
100.0
90.0
80.0
70.0
60.0
50.0
40.0
30.0
20.0
10.0
-0.0
0.0
20.0
40.0
60.0
80.0
100.0
120.0
Dose [%]
Cord + 0.5cm
PTV
Heart
Left Lung + Right Lung
Liver
Summed RT Plan 01
Coronal
Summed Beams in RT Plan 01
CT 01

Appendix 4: Problem Examples

A4.1: Introduction to Problem Examples

This section is intended to provide assistance to centres who may have limited experience of single phase planning and as such, attempts to illustrate and address some issues which have been found to arise in our experience. In oesophageal planning the size of the treatment site, it's proximity to multiple OARs and the inhomogeneous nature of the surrounding tissues mean that difficulties in achieving an optimal plan are often experienced. Here follows a series of examples which show how specific problems may be overcome to produce good results.

A4.2: Relative contribution of each beam

This example illustrates how increasing the relative contribution of the posterior beam in favour of the anterior beam contribution will achieve improved heart & liver sparing with no change to the lung dose. The figures below show two plans whose parameters are identical in all but one respect – the relative contributions of the anterior and posterior beams. The plan on the left has anterior and posterior beam contributions of 38% and 27% respectively whilst the plan on the right has anterior and posterior contributions of 28% and 37%.

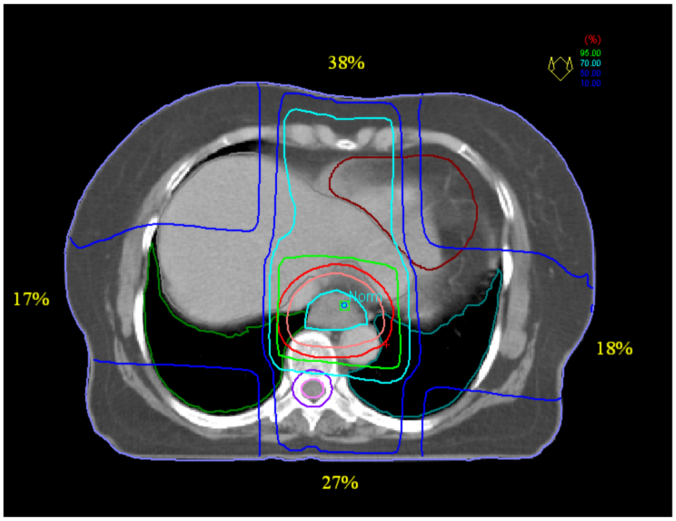

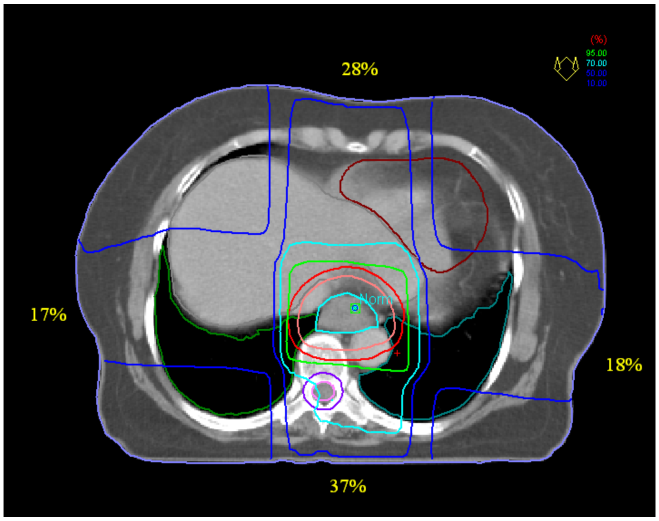

The dose volume histogram below shows a reduction in heart & liver dose, no change to the lung dose, and the cord dose remains within tolerance. In practice, increasing the cord dose to the tolerance level of 38Gy will result in the best possible sparing to the heart.

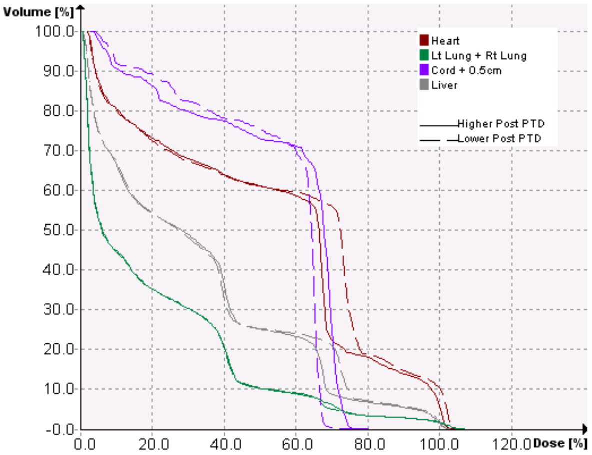

A4.3: Variations in lateral beam wedging

The suggested level of contribution of the posterior beam may be assisted by the use of 'anteriorly pointing' wedges on the lateral beams. However, where dynamic wedges are the preferred or are the only method of dose modulation available, their use may lead to reduced conformality of the MLCs to the PTV due to the need for the collimator to be positioned at 90° or 270°. Where EDW wedges are used, it may be possible to maintain conformality of the 95% isodose by increasing the wedge angle required on one of the lateral beams, and removing the wedge from the other. This action enables freedom of collimator angle and therefore optimal MLC positioning for one of the two lateral beams. The example below shows two plans – one having two anteriorly pointing 15 degree wedges on the lateral beams, and one having only one 30 degree wedge. The absence of the wedge on one of the lateral beams on the second plan has enabled more conformal MLC shielding and therefore improved the plan.

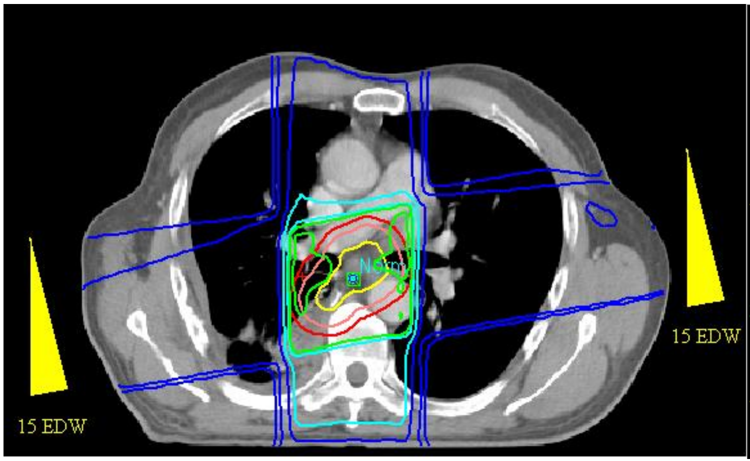

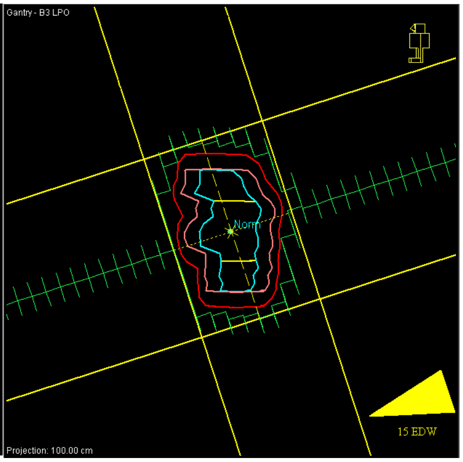

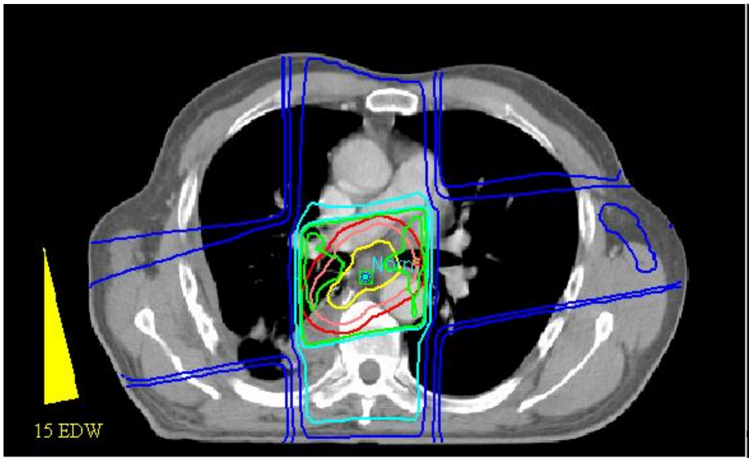

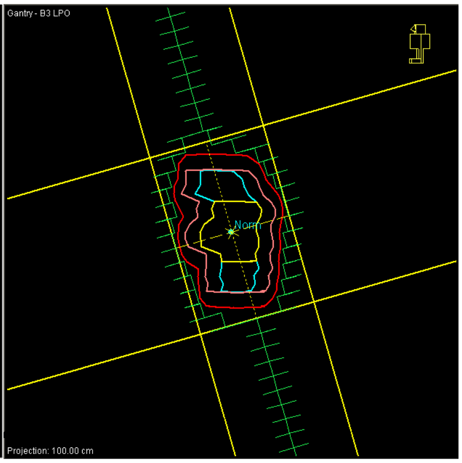

A4.4: Use of filler beams to lower hotspots within the PTV

The proximity of the oesophagus to lung tissue will often mean that difficulties are experienced in achieving a homogeneous dose throughout the PTV. The coronal sections below illustrate an example where hotspots seen in the lung tissue contained within the PTV have been reduced with the inclusion of a low weighted anterior ‘filler beam’ the ‘beams eye view’ for which is also shown below. In this case the filler beam delivers 5% of the tumour dose - the MLCs have been positioned in order to shield the hotspots and so improve the dose inhomogeneity within the PTV.

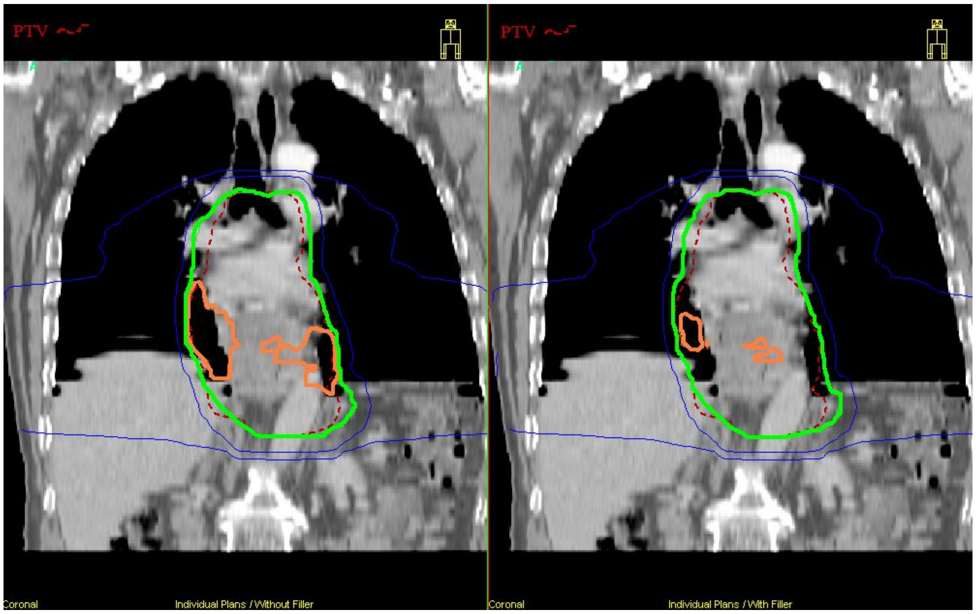

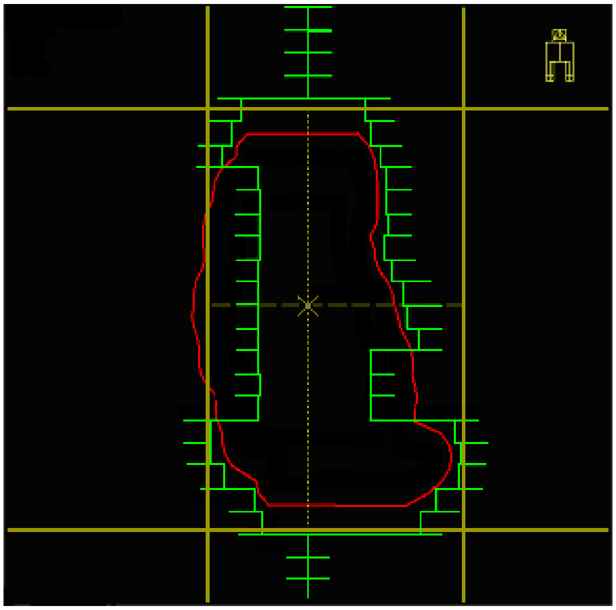

A4.5: Use of opposing superior – inferior wedged beams to improve coverage of 95% isodose and dose homogeneity within the PTV

Improvement in the coverage of the PTV with the 95% isodose may be seen by using a combination of two heavily wedged beams to replace a single un-wedged beam (usually the posterior or anterior beam). To illustrate this effect the image below shows the distribution from two 60 degree wedged beams (one pointing superiorly, and one pointing inferiorly). The important thing to note is that the isodoses at depth are around 10% higher at the superior and inferior extents of the beam than at the central axis, and so the combination will deliver more dose to both extremes of the PTV than can be delivered with a single unwedged beam. Furthermore, this combination allows a greater degree of freedom in plan optimisation to achieve dose homogeneity across the PTV as the two beams can be weighted as required to achieve the best balancing of the plan.

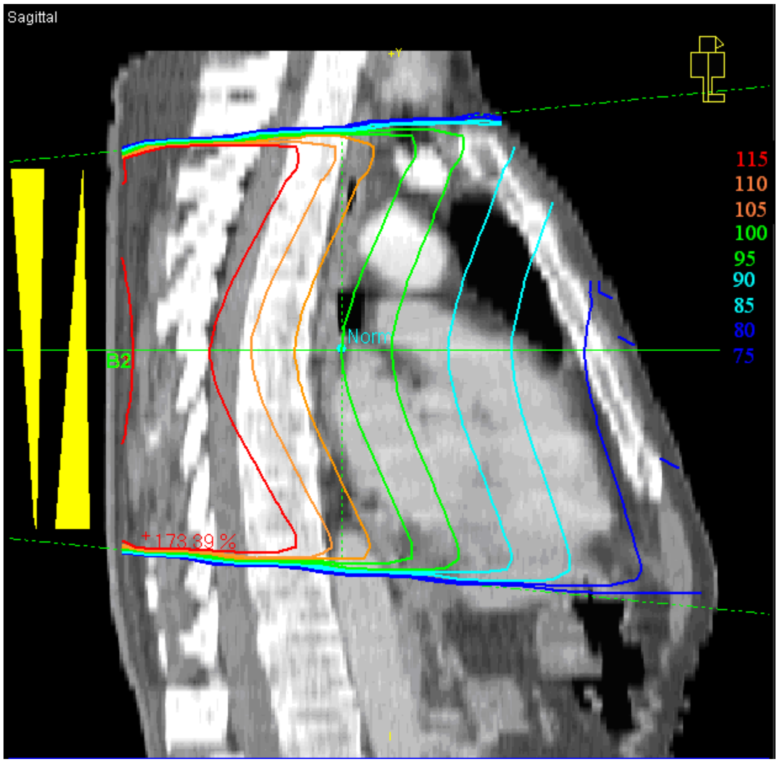

Authors

Dr T Crosby
Dr J Staffurth
Miss L Wills

Reviewers / Contributors

Dr A Crellin
Dr I Geh
Dr D Tait
Dr K Venables (NCRI Radiotherapy Clinical Studies Quality Assurance Group)

Wales Cancer Trials Unit

Contact Details

6th Floor, Neuadd Meirionnydd,
Heath Park
Cardiff
CF14 4YS

General Telephone Number 02920 687 500

Email: SCOPE1@WCTU.wales.nhs.uk

Visit our website – www.wctu.org.uk
