# Supplementary material for: Quality assurance of the SCOPE 1 trial in oesophageal radiotherapy
Source: Radiat Oncol. 2017 Nov 15;12:179. doi: 10.1186/s13014-017-0916-7 (PMC5688711; doi:10.1186/s13014-017-0916-7)
Supplement: Supplementary file 3 — SCOPE1 Trial Plan Assessment Form. (PDF 165 kb) [file 13014_2017_916_MOESM3_ESM.pdf]

Radiotherapy Plan Assessment Form

To be completed prior to start of Radiotherapy treatment. Fax to WCTU on 02920 687501

WCTU
WALES CANCER TRIALS UNIT
UNED YMCHWIL CANSER CYMRU

Patient Trial No. ☐☐☐☐☐☐ Patient Initials ☐☐☐ Patient Date of Birth ☐☐ ☐☐ ☐☐☐☐ (dd mm yyyy)

Treating Centre: ....

Date of Planning Scan: ☐☐ ☐☐ ☐☐ (dd mm yy) IV contrast solution used? Yes ☐ No ☐

TARGET VOLUME DELINEATION

Volume	Max	Patient
Disease length (EUS+CT±PET)	10 cm	
Outlined GTV length	10 cm	
CTVB length S-I	14 cm	
PTV length S-I	16 cm	
Anterior fields length S-I	17 cm	
GTV volume (cc)	NA	
CTVB volume (cc)	NA	
PTV volume (cc)	NA	

Target Margins	Protocol	Patient
GTV to CTVB S-I	2.0 cm	
GTV to CTVB Lat/AP*	1.0 cm	
CTV definition inferiorly (delete as appropriate)	OES / STOMACH / MIXED	
*CTVB post margin altered?	Yes ☐	No ☐
Extent CTVB modification, from		to
CTVB to PTV S-I	1.0 cm	
CTVB to PTV Lat/AP	0.5 cm	

PRESCRIPTION

Prescribed dose		Gy in		Fractions	N.B. Protocol prescription 50Gy in 25#

DOSE REPORTING - Organs at Risk

OAR	Level	Objective	Achieved	Comments	
Combined lungs	Volume receiving 20Gy	<25%		%	
Heart	Volume receiving 40Gy	<30%		%	
Liver	Volume receiving 30Gy	<60%		%	
Rt kidney	Volume receiving 20Gy	<25%		%	
Lt kidney	Volume receiving 20Gy	<25%		%	
Spinal Cord PRV	Volume receiving 40Gy	0%		%	
	Dose to 1cc	<40Gy		Gy	

ICRU defined maximum dose	Objective: Max 107%/ 53.5Gy	Achieved:		% / Gy

Completed by: Name ……………………… Signed ………………… ☐☐ ☐☐ ☐☐ (d mm yy)

WCTU USE ONLY: Data input on MACRO by: ________________________________ _____/_____/_______

Radiotherapy Plan Assessment Form

To be completed prior to start of Radiotherapy treatment. Fax to WCTU on 02920 687501

Patient Trial No. ☐☐☐☐☐☐ Patient Initials ☐☐☐ Patient Date of Birth ☐☐ ☐☐ ☐☐☐☐
dd mm yyyy

DOSE REPORTING - PTV

TPS:.. **Version:**..

Algorithm Used ....

Complete this section where **Type A algorithm** has been used (e.g. Pencil Beam, Fast Fourier Transform)

PTV	Objective	Achieved	Comments
Volume of PTV receiving 95% dose	≥99%	%	

Complete this section where **Type B Algorithm** has been used (e.g. Collapsed Cone, Multigrid Superposition, Anisotropic Analytical Algorithm, Monte Carlo)

% lung region overlap with PTV: …………….. % of PTV volume

Objective for PTV coverage by 95% dose for Type B algorithms may be calculated using the formula from the revised protocol:

V95% ≥ 99 – (0.4 x %PTV overlap)

PTV	Objective	Achieved	Comments
Volume of PTV receiving 95% dose	≥................. From formula	%	

Is there any deviation from the protocol? Yes ☐ No ☐
(If yes please discuss with trial team and comment on reason)

Comments:...

....

....

Planned: Name .... Signed .... ☐☐ ☐☐ ☐☐ (d mm yy)

Checked: Name .... Signed .... ☐☐ ☐☐ ☐☐ (d mm yy)

Accepted by treating **consultant**: Name .... Signed .... ☐☐ ☐☐ ☐☐ (d mm yy)

Completed by: Name ……………………… Signed ………………… ☐☐ ☐☐ ☐☐ (d mm yy)

WCTU USE ONLY: Data input on MACRO by: ___________________________________ _____/_____/_______
